# Sex-specific associations between sodium and potassium intake and overall and cause-specific mortality: a large prospective U.S. cohort study, systematic review, and updated meta-analysis of cohort studies

**DOI:** 10.1186/s12916-024-03350-x

**Published:** 2024-03-22

**Authors:** Lu Gan, Bin Zhao, Maki Inoue-Choi, Linda M. Liao, Barry I. Graubard, Stephanie J. Weinstein, Demetrius Albanes, Jiaqi Huang

**Affiliations:** 1 National Clinical Research Center for Metabolic Diseases, Metabolic Syndrome Research Center, Key Laboratory of Diabetes Immunology, Ministry of Education, and Department of Metabolism and Endocrinology, The Second Xiangya Hospital of Central South University; Xiangya School of Public Health, Central South University; CSU-Sinocare Research Center for Nutrition and Metabolic Health; Furong Laboratory, Changsha, Hunan 410011 China; 2 Division of Cancer Epidemiology and Genetics, Department of Health and Human Services, National Cancer Institute, NIH, Bethesda, MD USA

**Keywords:** Sodium and potassium intake, Sex-specific, Overall mortality, Cause-specific mortality, Multivariate analysis, Systematic review and meta-analysis

## Abstract

**Background:**

The impact of sodium intake on cardiovascular disease (CVD) health and mortality has been studied for decades, including the well-established association with blood pressure. However, non-linear patterns, dose–response associations, and sex differences in the relationship between sodium and potassium intakes and overall and cause-specific mortality remain to be elucidated and a comprehensive examination is lacking. Our study objective was to determine whether intake of sodium and potassium and the sodium–potassium ratio are associated with overall and cause-specific mortality in men and women.

**Methods:**

We conducted a prospective analysis of 237,036 men and 179,068 women in the National Institutes of Health-AARP Diet and Health Study. Multivariable-adjusted Cox proportional hazard regression models were utilized to calculate hazard ratios. A systematic review and meta-analysis of cohort studies was also conducted.

**Results:**

During 6,009,748 person-years of follow-up, there were 77,614 deaths, 49,297 among men and 28,317 among women. Adjusting for other risk factors, we found a significant positive association between higher sodium intake (≥ 2,000 mg/d) and increased overall and CVD mortality (overall mortality, fifth versus lowest quintile, men and women HRs = 1.06 and 1.10, P_nonlinearity_ < 0.0001; CVD mortality, fifth versus lowest quintile, HRs = 1.07 and 1.21, P_nonlinearity_ = 0.0002 and 0.01). Higher potassium intake and a lower sodium–potassium ratio were associated with a reduced mortality, with women showing stronger associations (overall mortality, fifth versus lowest quintile, HRs for potassium = 0.96 and 0.82, and HRs for the sodium–potassium ratio = 1.09 and 1.23, for men and women, respectively; P_nonlinearity_ < 0.05 and both P for interaction ≤ 0.0006). The overall mortality associations with intake of sodium, potassium and the sodium–potassium ratio were generally similar across population risk factor subgroups with the exception that the inverse potassium-mortality association was stronger in men with lower body mass index or fruit consumption (P_interaction_ < 0.0004). The updated meta-analysis of cohort studies based on 42 risk estimates, 2,085,904 participants, and 80,085 CVD events yielded very similar results (highest versus lowest sodium categories, pooled relative risk for CVD events = 1.13, 95% CI: 1.06–1.20; P_nonlinearity_ < 0.001).

**Conclusions:**

Our study demonstrates significant positive associations between daily sodium intake (within the range of sodium intake between 2,000 and 7,500 mg/d), the sodium–potassium ratio, and risk of CVD and overall mortality, with women having stronger sodium–potassium ratio-mortality associations than men, and with the meta-analysis providing compelling support for the CVD associations. These data may suggest decreasing sodium intake and increasing potassium intake as means to improve health and longevity, and our data pointing to a sex difference in the potassium-mortality and sodium–potassium ratio-mortality relationships provide additional evidence relevant to current dietary guidelines for the general adult population.

**Systematic review registration:**

PROSPERO Identifier: CRD42022331618.

**Supplementary Information:**

The online version contains supplementary material available at 10.1186/s12916-024-03350-x.

## Background

Sodium and potassium are essential elements for human health that play critical roles in maintaining homeostatic physiology related to intravascular volume, acid–base balance, cell membrane function, and immune modulation [1–3]. Evidence from randomized controlled trials and observational studies has accumulated regarding increased risk of hypertension associated with higher sodium and lower potassium intake, respectively [1, 4–6]. Even though relationships between blood pressure and risk of cardiovascular disease (CVD) and mortality are well-established [7–10], the precise role of sodium and potassium intake in overall and cause-specific mortality, and sex-specific associations remains uncertain. Growing evidence suggested strong positive linear associations based on prospective cohort studies, as well as meta-analyses that include cohort studies and controlled randomized trials [1, 11–16]. Also, multiple recent large cohort studies have demonstrated U-shaped or J-shaped associations between sodium intake and CVD and overall mortality [6, 17–20]. However, the Health ABC Study with 2,642 older adults ages 70 to 79 years showed that FFQ-based sodium intake was not associated with an increased risk of 10-year mortality [21]. Additionally, in the Hypertensive Cohort with 3,505 hypertensive patients, the authors reported no significant associations for the CVD outcomes with greater sodium intake that was estimated by 24-h urine excretion [22]. A comprehensive examination of the association between dietary sodium, potassium and the sodium–potassium intake ratio and mortality in a long-term follow-up has not yet been conducted, particularly with respect to cause-specific mortality, patterns and dose–response associations, and sex and race differences.

Most current dietary guidelines recommend reduced sodium intake (i.e., < 2,000–2,300 mg/day), with slightly different recommendations among the World Health Organization (WHO, < 2,000 mg/day), American Heart Association (< 2,300 mg/day in healthy adults and < 1,500 mg/day in high-risk individuals) and U.S. Dietary guidelines (< 2,300 mg/day). Average daily sodium intakes vary considerably between men and women, with a range of difference in daily intake being approximately 500–1,400 mg [11–13, 21]. However, sex differences in sodium intake have not been clearly represented in the dietary guidelines.

To this end, we examined whether dietary sodium and potassium are associated with mortality among men and women based on the National Institutes of Health (NIH)-AARP Diet and Health Study, a large U.S. cohort of more than 400,000 participants and 77,000 deaths occurring during 16 years of cohort observation. Additionally, we aimed to elucidate the sex-specific risk patterns and dose–response associations, the specific role of the sodium–potassium intake ratio, and cause-specific mortality, including death from CVD, heart disease, stroke, cancer, respiratory disease, injuries/accidents (as neutral controls) and other causes. To further investigate these findings, we conducted an updated meta-analysis of the association between sodium intake and risk of CVD and CVD mortality, which included the present study.

## Methods

### Study population

Details of the NIH-AARP Diet and Health Study have been documented previously [23]. From 1995 through 1996, a total of 617,119 participants, ages 50 to 71 years and enrolled from six states (California, Florida, Louisiana, New Jersey, North Carolina, and Pennsylvania) and two metropolitan areas (Atlanta, Georgia; and Detroit, Michigan), returned a baseline questionnaire regarding diet, lifestyle and demographic characteristics. Based on the data quality of dietary questionnaire responses, 566,398 men and women constituted the final baseline cohort. Of these, we excluded 15,760 individuals with proxy questionnaires, and 51,334 individuals with a prevalent cancer (excluding nonmelanoma skin cancer), 73,549 with heart disease or stroke, 997 with self-reported end-stage kidney disease, 4,253 individuals having only a death record for cancer, 4,372 individuals with an extreme daily caloric consumption, and 29 individuals with zero years of follow-up. After these exclusions, the final analytic cohort consisted of 416,104 participants comprising 237,036 men and 179,068 women. The Study was approved by the Special Studies Institutional Review Board of the U.S. National Cancer Institute, and all participants provided informed consent by completing and returning the questionnaires.

### Exposure assessment

In addition to questionnaires of baseline demographic and lifestyle characteristics, dietary information was collected using a self-administered National Cancer Institute Diet History Questionnaire (DHQ) [23]. The DHQ contained 124 food items, portion sizes, and frequency of intake over the past 12 months. Nutrient intake and food group consumptions were determined based on the U.S. Department of Agriculture 1994–1996 Continuing Survey of Food Intakes by Individuals food composition nutrient database, that reflected an accurate representation for food intake, nutrition and dietary habits of the general U.S. population [24]. The food frequency questionnaire used in the present study was calibrated by two nonconsecutive 24-h dietary recalls in a subgroup of study participants (*n* = 1,953) [25]. When comparing the DHQ with the 24-h recalls, the correlation coefficients of mineral intake ranged from 0.49 to 0.67 (all *P* values < 0.001) [25].

### Mortality assessment

Participants were followed from the date of the baseline questionnaire (1995–1996) that was returned until the date of death or December 31, 2011, whichever occurred first. Vital status of participants was collected through periodic linkage with the Social Security Administration Death Master File. Causes of death information was extracted by the linkage with the National Death Index Plus, and classified based on codes from the International Classification of Diseases, Ninth (ICD-9) and 10th (ICD-10) Revisions (Additional file 1: Table S1).

### Statistical analysis

Intakes of sodium and potassium and the sodium–potassium ratio were tabulated based on gender and a number of lifestyle and dietary factors. We used cause-specific Cox proportional hazards regression models with person-time as the underlying time metric to calculate hazard ratios (HRs) and their 95% confidence intervals (CIs) for the associations of dietary intakes of sodium, potassium and sodium–potassium ratio with risk of overall and cause-specific mortality, with risk estimates for men and women reported separately. We observed no violation of the proportional-hazards assumption, assessed by modeling the interaction of intervals of follow-up time with exposure categorical variables, and the significance of *P* values for interaction were determined through likelihood ratio tests. Multivariable analyses were adjusted for baseline factors including age, race or ethnic group, body mass index (BMI), alcohol consumption, smoking status (never, former, current or missing), physical activity, education, marital status, diabetes mellitus (yes vs. no), self-reported health status, vitamin supplement use (yes vs. no), and total energy intake. To evaluate more parsimonious models, we adjusted for age, BMI, alcohol consumption, smoking status, physical activity, race or ethnic group, education, self-reported health status, and total energy intake. For analysis of sodium intake, models were additionally adjusted for the Healthy Eating Index 2015 (HEI-2015) score, excluding the sodium component; for potassium intake and the sodium–potassium ratio, models were additionally adjusted for HEI-2015 components for sodium (potassium model only), seafood and plant protein, saturated fat, fatty acids and refined grains. The HEI-2015 is comprised of 13 dietary components and was originally created to evaluate adherence to an overall healthy dietary pattern based on U.S. Dietary Guidelines 2015–2020 (for details of HEI-2015 score criteria, see Additional file 1: Table S2) [26].

In our primary analyses, we used cubic-restricted splines with four-knots (selected at the 5^th^, 25^th^, 75^th^ and 95^th^ percentiles) to evaluate non-linearity of associations between dietary intakes of sodium, potassium and sodium–potassium ratio and each mortality endpoint, and HRs were based on Cox proportional hazard models. The reference values for the estimated hazard ratio curves for dietary sodium, potassium and sodium–potassium ratio were 2,300 mg (values recommended by dietary guideline), 2,044 mg (10% percentile cutoff) and 0.59 (10% percentile cutoff), respectively. In order to elucidate and quantify the HRs and their corresponding 95% CIs of the cubic-restricted splines, the exposure variables of sodium, potassium and sodium–potassium ratio were categorized based on the curves derived from the cubic-restricted splines. The adjusted absolute risk difference (ARD) was estimated for the highest category versus the referent category of the dietary exposure variables (i.e., the second lowest category for sodium, and the lowest category for potassium and the sodium–potassium ratio). The 95% CIs for ARD were computed based on 100 bootstrap samples. We found wide 95% CIs among individuals with an intake range of sodium lower than 2,000 mg/d, and a lower sodium intake may reflect poor intake of all nutrients; i.e., a sign of anorexia, sub-clinical illnesses, or unidentified comorbidity [27]. In order to compare our findings with previous studies [11], we therefore also categorized sodium (≥ 2,000 mg/d), potassium and sodium–potassium ratio (> 0.17 with sodium ≥ 2,000 mg/d) according to their quintile distribution to estimate the sex-stratified HRs and 95% CIs, with the lowest quintile serving as the reference category. We used likelihood ratio tests to assess the overall and non-linear statistical significance for the three exposures. To obtain relative risk estimates based on linear trend in the continuous value exposures, we estimated HRs and the corresponding 95% CIs for the associations between sodium intake, potassium intake, and sodium–potassium ratio (per standard deviation [SD] change in these variables) and overall mortality and cause-specific mortality by including these exposures as continuous variables in the Cox proportional hazard regression models.

Sex-stratified subgroup analyses of the dietary sodium, potassium, and sodium–potassium ratio associations with overall and cause-specific mortality were analyzed by race/ethnicity and the following characteristics defined a priori: age at baseline (< 60, 60 to < 65, or ≥ 65 years), race/ethnicity (Non-Hispanic white; Non-Hispanic black; and Hispanic, total minority racial group and other), cigarette smoking status (never, former, or current), diabetes history (no or yes), BMI (weight divided by height [kg/m^2^]; < 18.5, 18.5 to < 25, 25 to < 30, and ≥ 30), alcohol consumption (0 to < 1, 1 to < 3, and ≥ 3 drinks per day), consumption of fruits and vegetables (median split: low or high), supplemental vitamin use (no or yes), HEI-2015 score (excluding the sodium component, as low, median or high tertiles), self-reported health status (poor to fair, good, very good to excellent), and follow-up years (0 to < 5, 5 to < 10, and ≥ 10 years). Statistical significance of such possible effect modification was based on likelihood ratio tests that included multiplicative interaction terms for the exposure categorical variables and each selected stratified factor entered into the Cox proportional hazard regression multivariable-adjusted models and compared models with and without the interaction terms.

Sex-stratified sensitivity analyses were performed for overall and cause-specific mortality outcomes which: 1) only adjusted for age at baseline and race or ethnic group; 2) reduced bias from reverse causality by excluding the first five years of follow-up; 3) excluded participants with self-reported diabetes at study entry; and, 4) excluded participants reporting poor to fair health status or unknown health status at study entry; 5) additionally adjusted for dietary potassium intake for the sodium-mortality associations; 6) additionally adjusted for family income in regression models.

All analyses were performed using SAS software (version 9.4, SAS Institute Inc.) and R version 3.6.1 (R Foundation for Statistical Computing, Vienna, Austria). All reported *P* values are two-sided with the type I error rate of 0.05. To account for multiple outcome comparisons, statistical significance was based on a Bonferroni correction threshold set at 0.00093 for primary and subgroup analyses (0.05/[9 × 3 × 2], seven mortality endpoints [mortality from 1) any cause, 2) CVD, 3) heart disease, 4) stroke, 5) cancer, 6) respiratory disease, 7) infectious disease, 8) injury and accidents, and 9) other causes combined], three primary exposure variables of sodium, potassium, and sodium–potassium ratio, as well as two sexes of men and women), and at 0.00076 for subgroup interaction tests (0.05/[11 × 3 × 2], 11 selected factors), respectively.

### Systematic review and meta-analysis of associations between sodium intake and risk of CVD and CVD mortality

We conducted a systematic search and meta-analysis, including the present study data and published prospective cohort analyses, that investigated the associations between sodium intake and risk of CVD and CVD mortality in adult populations. We carried out the meta-analysis based on the Preferred Reporting Items for Systematic reviews and Meta-Analysis (PRISMA) [28], and our systematic review protocol has been registered in the PROSPERO database (CRD42022331618). We conducted a systematic search through 8 June 2023 of three online databases, including Web of Science, PubMed and Embase.

Two investigators (L.G. and J.H.) independently reviewed the relevant literature and extracted the following information: author and publication year; study population and cohort name; sample size; study location (country/region), years of outcome follow-up; baseline age range of participants; exposure assessment methods; sodium intake categories; outcome ascertainment methods; relative risks (RRs), 95% CIs, and covariables from fully adjusted models. If there were two studies using the same cohort/study population, and conducted similar analyses for the same CVD endpoint, we selected the larger study of longer follow-up and greater event numbers to include in our meta-analysis. Regarding sodium intake, we used the median or midpoint value for each category. If the upper bound for the highest category was not available, the upper bound was calculated by multiplying the lower bound by 1.75 [29, 30].

We calculated the pooled RRs of CVD risk and mortality comparing the highest versus lowest category of sodium intake using meta-analysis random-effects models. The total CVD risk (including CVD mortality) estimate from each study was used when available. When the study did not report total CVD events, a fixed-effects model meta-analysis was performed to pool risk estimates for CVD death, coronary heart disease, and stroke [31], or CVD incidence and CVD death [32]. Where the study provided data for men and women separately, we treated them as distinct studies. The Cochran Q-test and the I^2^ statistic were used to examine between-study heterogeneity, along with removing one study at a time from the meta-analysis to identify which contributed to heterogeneity in the pooled RR estimate. We also performed subgroup meta-analyses stratified by sex, number of participants, duration years of follow-up, number of events, geographical location, risk of bias, and dietary assessment method. In the meta-analysis, we assessed the possible nonlinear dose–response association using cubic restricted spline regression with three knots at the 5th, 50th, and 95th percentiles of the sodium intake distribution [33]. Individual participating studies with at least 3 exposure categories were included in the final dose–response meta-analysis.

Egger tests and funnel plots were conducted to determine potential publication bias. The Newcastle–Ottawa Scale was used to examine possible biases for eligible studies. Stata version 16.0 was used to perform statistical analyses for meta-analysis.

## Results

### Dietary sodium, potassium, sodium–potassium ratio and lifestyle factors

Table 1 provides baseline characteristics of the cohort according to sex and categories of estimated dietary intakes of sodium, potassium and sodium–potassium ratio. The median daily intakes of sodium and potassium were 2,812 mg and 3,364 mg for men, and 2,192 mg and 2,916 mg for women, respectively. Participants with greater sodium intake in both sexes were more likely to have diabetes, higher BMI and intake of total energy, be physically active, be current smokers or report poor or fair health, and to have lower educational level, lower HEI-2015 score, and higher intake of fiber, fruit and vegetable.

**Table 1:** Baseline characteristics of cohort participants according to categories of sodium and potassium intake and the sodium–potassium ratio^a, b^

	**Categories of sodium intake (≥ 2,000 mg/d)**			**Categories of potassium intake**			**Categories of sodium–potassium ratio (sodium ≥ 2,000 mg/d)**		
**Men**	Q1	Q3	Q5	Q1	Q3	Q5	Q1	Q3	Q5
Sodium, mean (SD), mg/d	2183.53 (103.91)	2975.93 (137.31)	5090.90 (1155.20)	1720.94 (553.04)	2751.73 (688.07)	4433.94 (1449.23)	2875.65 (807.78)	3333.08 (1050.83)	3968.53 (1469.07)
Potassium, mean (SD), mg/d	2883.49 (752.28)	3571.61 (857.46)	5308.32 (1518.88)	1835.57 (345.52)	3177.71 (173.46)	5533.11 (1233.87)	4807.60 (1475.10)	3889.58 (1224.29)	3340.47 (1244.06)
Sodium–potassium ratio	0.81 (0.20)	0.88 (0.20)	1.00 (0.22)	0.95 (0.28)	0.87 (0.21)	0.81 (0.21)	0.61 (0.07)	0.86 (0.03)	1.20 (0.16)
Age, years	62.01 (5.37)	61.79 (5.36)	61.13 (5.38)	61.89 (5.41)	61.88 (5.37)	61.42 (5.37)	61.98 (5.34)	61.74 (5.38)	61.18 (5.42)
Body mass index, kg/m^2^	26.83 (4.12)	27.10 (4.10)	27.96 (4.82)	27.05 (4.24)	27.06 (4.17)	27.55 (4.56)	26.87 (4.12)	27.22 (4.24)	27.74 (4.70)
Non-Hispanic white, %	92.65	94.09	92.15	87.11	93.64	92.43	92.80	93.89	91.59
Vigorous physical activity (≥ 5/week), %	19.12	21.44	24.52	14.82	19.51	27.19	27.52	21.57	18.28
Education (college or postgraduate), %	48.29	47.74	40.73	42.81	47.71	44.13	48.86	47.66	40.28
Married, %	84.98	86.46	84.97	81.85	86.37	84.10	83.01	86.84	85.75
Family history of cancer, %	47.29	48.02	47.19	45.22	47.49	47.09	47.34	48.18	47.18
History of diabetes mellitus, %	7.09	7.79	11.05	7.96	7.73	9.35	6.69	8.09	10.83
Current smoker, %	9.37	10.23	13.64	9.89	10.33	12.38	9.86	10.51	12.89
Alcoholic drinks (> 3), %	10.33	11.34	12.98	7.94	10.70	13.90	13.24	11.60	10.19
Energy intake, mean (SD), kcal	1566.10 (408.14)	2018.93 (455.63)	3164.33 (802.01)	1182.47 (410.64)	1843.97 (459.44)	2964.36 (877.25)	2231.49 (808.95)	2194.76 (739.77)	2383.74 (884.76)
Fiber intake, mean (SD), g/1000 kcal	15.98 (5.79)	20.40 (6.80)	31.46 (11.85)	10.44 (3.78)	18.16 (4.96)	31.47 (11.51)	25.54 (11.40)	22.52 (9.50)	20.72 (9.14)
Foods, servings, mean (SD)									
Fruit	2.62 (2.06)	2.95 (2.27)	3.84 (3.14)	1.39 (0.96)	2.51 (1.46)	4.88 (3.51)	5.36 (3.84)	2.97 (1.86)	1.87 (1.35)
Vegetables	2.95 (1.34)	3.94 (1.66)	6.59 (3.21)	1.94 (0.95)	3.50 (1.37)	6.35 (3.18)	4.67 (2.76)	4.54 (2.45)	4.22 (2.36)
HEI-2015 score, mean (SD)	67.81 (9.59)	66.89 (9.47)	64.10 (9.63)	63.13 (9.98)	66.64 (9.51)	68.48 (9.36)	72.66 (8.57)	67.63 (8.12)	58.74 (8.76)
Vitamin supplement use, %	52.32	52.45	53.04	47.24	52.18	55.41	57.02	53.27	48.29
Self-reported health (poor or fair), %	6.13	6.62	9.27	8.35	6.57	7.71	1.32	1.22	1.29
**Women**	Q1	Q3	Q5	Q1	Q3	Q5	Q1	Q3	Q5
Sodium, mean (SD), mg/d	2176.00 (103.90)	2964.10 (136.30)	4920.17 (962.51)	1507.61 (504.09)	2412.59 (637.80)	3830.29 (1264.42)	2695.21 (679.75)	3027.15 (910.96)	3393.82 (1198.34)
Potassium, mean (SD), mg/d	2963.95 (770.26)	3700.75 (926.38)	5450.09 (1547.95)	1773.89 (374.16)	3167.83 (174.64)	5443.99 (1162.98)	4556.40 (1309.31)	3537.26 (1059.38)	2862.71 (1036.60)
Sodium–potassium ratio	0.78 (0.20)	0.85 (0.21)	0.95 (0.22)	0.86 (0.26)	0.76 (0.20)	0.71 (0.20)	0.60 (0.08)	0.86 (0.03)	1.20 (0.16)
Age, years	61.72 (5.39)	61.48 (5.41)	61.16 (5.39)	61.58 (5.46)	61.68 (5.37)	61.58 (5.40)	61.88 (5.39)	61.48 (5.36)	60.92 (5.38)
Body mass index, kg/m^2^	26.46 (5.69)	27.15 (6.23)	28.43 (7.14)	26.74 (5.94)	26.60 (5.76)	26.99 (6.31)	26.10 (5.59)	27.18 (6.12)	28.56 (7.06)
Non-Hispanic white, %	91.49	91.10	84.00	87.44	91.49	85.79	89.33	91.25	87.42
Vigorous physical activity (≥ 5/week), %	16.52	16.49	18.33	12.38	16.67	21.95	22.17	16.06	11.78
Education (college or postgraduate), %	32.58	31.32	25.65	28.11	32.11	29.85	33.37	31.00	25.21
Married, %	46.41	48.48	44.44	41.47	47.28	43.58	44.40	48.68	46.60
Family history of cancer, %	51.60	52.07	48.53	50.75	51.57	49.55	50.77	51.59	50.68
Diabetes mellitus, %	5.35	7.02	9.96	5.85	5.90	6.80	4.85	6.72	10.17
Current smoker, %	13.40	13.96	15.57	14.64	13.99	14.10	13.43	13.37	16.21
Alcoholic drinks (> 3), %	2.78	2.67	2.99	2.67	2.74	2.79	2.57	2.89	3.43
Energy intake, mean (SD), kcal	1468.76 (277.63)	1908.73 (341.90)	2945.21 (648.15)	1013.50 (337.23)	1591.82 (387.03)	2515.19 (690.06)	1918.86 (559.70)	1896.48 (587.48)	2004.83 (709.16)
Fiber intake, mean (SD), g/1000 kcal	16.68 (5.52)	21.60 (6.90)	33.52 (12.44)	10.14 (3.75)	18.00 (4.94)	31.12 (11.29)	24.64 (10.24)	21.32 (8.77)	18.48 (8.01)
Foods, servings, mean (SD)									
Fruit	2.84 (2.07)	3.33 (2.48)	4.62 (3.53)	1.50 (1.00)	2.89 (1.51)	5.83 (3.75)	5.23 (3.50)	2.91 (1.70)	1.73 (1.24)
Vegetables	3.52 (1.57)	4.71 (2.03)	7.84 (4.00)	2.11 (1.06)	3.84 (1.58)	6.97 (3.64)	5.26 (2.98)	4.77 (2.62)	4.07 (2.30)
HEI-2015 score, mean (SD)	69.29 (9.15)	68.52 (9.16)	66.40 (9.18)	65.44 (9.81)	69.81 (8.88)	71.52 (8.35)	73.77 (7.65)	68.29 (7.84)	59.61 (8.71)
Vitamin supplement use, %	61.63	61.92	61.15	56.69	62.08	64.65	65.19	62.14	55.72
Prior or current postmenopausal hormone therapy, %	55.38	53.44	47.44	54.24	55.04	49.28	52.50	54.90	51.25
Self-reported health (poor or fair), %	8.59	10.15	14.98	10.67	8.86	10.53	60.77	53.92	43.17

^a^Dietary data and alcohol consumption shown per day

^b^All exposures are associated with dietary intakes of sodium, potassium and sodium–potassium ratio, with P values < 0.0001 (except for the associations between vitamin supplement use and sodium intake among men and women, *P* value > 0.05; alcoholic drinks (> 3) and potassium intake among women, *P* value > 0.05; family history of cancer and sodium–potassium ratio among women, *P* value > 0.05; family history of cancer and sodium intake among men, *P* value = 0.037; age and potassium intake among women, *P* value = 0.0021; and family history of cancer and sodium–potassium ratio among men, *P* value = 0.035). *P* values are calculated based on ANOVA tests for continuous variables, and Chi-Square tests for categorical variables, respectively

### Dietary sodium and overall and cause-specific mortality

Participants in this cohort had a median age of 62 years (interquartile range [IQR]: 57–66) at baseline and a median follow-up of 15.5 years (IQR: 15.5–15.8). During a total of 6,009,748 person-years of follow-up, there were 77,614 deaths (49,297 men and 28,317 women), including 22,228 from CVD (14,647 men and 7,581 women), of which 17,901 were from heart disease (12,178 men and 5,723 women) and 3,748 from stroke (2,153 men and 1,595 women), 28,099 from cancer (17,816 men and 10,283 women), 5,606 from respiratory disease (3,135 men and 2,471 women), 2,923 from infectious disease (1,796 men and 1,127 women), 2,614 from injuries and accidents (1,878 men and 736 women), and 16,144 from other causes combined (10,025 men and 6,119 women).

**Fig. 1:**
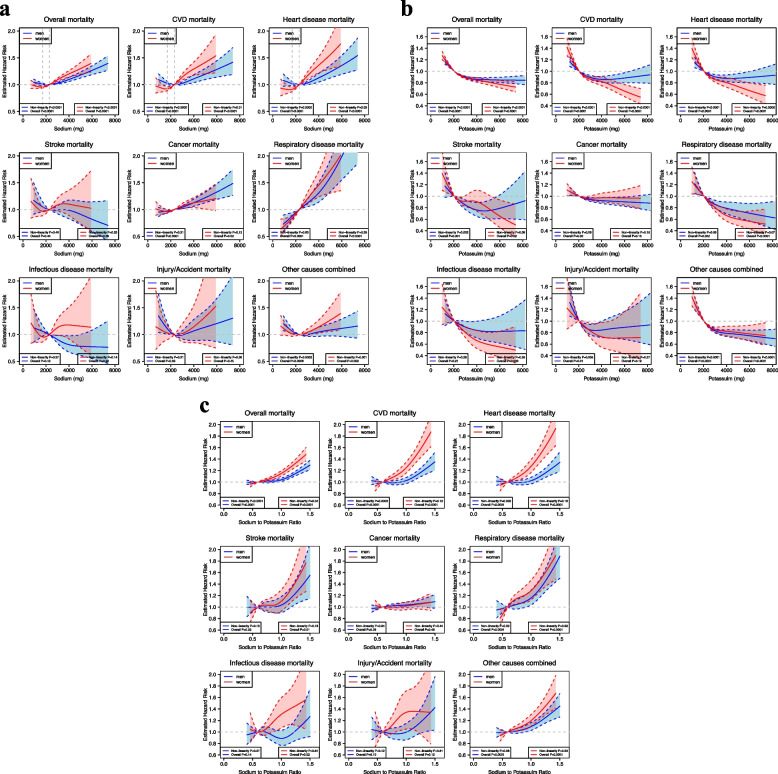
**a** Sex Stratified Associations Between Sodium Intake and Overall and Cause-specific Mortality in Multivariable-Adjusted Cubic Spline Regression Models. Analyses were adjusted for age at baseline, BMI, alcohol consumption, smoking status (never, former, current or missing), physical activity, race or ethnic group, education, marital status, diabetes (yes vs. no), health status, vitamin supplement use, total energy intake, and the Healthy Eating Index 2015 (HEI-2015) score excluding the sodium component. For women, the risk estimates were additionally adjusted for postmenopausal hormone therapy (yes vs. no). The solid line denotes the HR of overall mortality according to sodium intake with a four-knot cubic spline selected at the 5th, 25th, 75th, and 95th percentiles of intake, dashed lines and shaded areas represent the 95% confidence intervals, blue indicates men and red indicates women. **b** Sex Stratified Associations Between Potassium Intake and Overall and Cause-specific Mortality in Multivariable-Adjusted Cubic Spline Regression Models. Analyses were adjusted for age at baseline, BMI, alcohol consumption, smoking status (never, former, current or missing), physical activity, race or ethnic group, education, marital status, diabetes (yes vs. no), health status, vitamin supplement use, total energy intake, and the Healthy Eating Index 2015 (HEI-2015) score components of sodium, seafood and plant protein, saturated fat, fatty acids and refined grains. For women, the risk estimates were additionally adjusted for postmenopausal hormone therapy (yes vs. no). The solid line denotes the HR of overall mortality according to potassium intake with a four-knot cubic spline selected at the 5th, 25th, 75th, and 95th percentiles of intake, dashed lines and shaded areas represent the 95% confidence intervals, blue indicates men and red indicates women. **c** Sex Stratified Associations Between Sodium–Potassium Ratio and Overall and Cause-specific Mortality in Multivariable-Adjusted Cubic Spline Regression Models. Analyses were adjusted for age at baseline, BMI, alcohol consumption, smoking status (never, former, current or missing), physical activity, race or ethnic group, education, marital status, diabetes (yes vs. no), health status, vitamin supplement use, total energy intake, and the Healthy Eating Index 2015 (HEI-2015) score components of seafood and plant protein, saturated fat, fatty acids and refined grains. For women, the risk estimates were additionally adjusted for postmenopausal hormone therapy (yes vs. no). The solid line denotes the HR of overall mortality according to sodium–potassium ratio with a four-knot cubic spline selected at the 5th, 25th, 75th, and 95th percentiles of intake, dashed lines and shaded areas represent the 95% confidence intervals, blue indicates men and red indicates women

Figure 1a demonstrates the multivariable-adjusted associations between dietary sodium and mortality, with the HRs and corresponding 95% CIs of the cubic-restricted splines shown in the supplemental data (Additional file 1: Tables S3 and S4). Our results show substantially the same associations based on minimally adjusted and parsimoniously adjusted models (Additional file 1: Figures S1 and S4). Table 2 and supplemental data (Additional file 1: Table S5) present the estimated HRs and their 95% CIs based on quintiles, per 500 mg or per SD change in sodium intake for the associations with the risk of overall mortality and cause-specific mortality. We found statistically significant non-linear associations between sodium intake and overall mortality in men and women (P_nonlinearity_ < 0.0001), with the nadir of mortality occurring at intake levels of approximately 2,300 mg for men and 1,700 mg for women (Fig. 1a). Among participants with a daily sodium intake of 2,000 mg and above, a higher intake of sodium was associated with an increased risk of overall mortality for both men and women (fifth versus first quintile, HR for men = 1.06, 95% CI: 1.02, 1.09; HR for women = 1.10, 95% CI: 1.04, 1.16; both P_trend_ ≤ 0.0002; Table 2). With the daily intake range of 2,000- < 3,000 mg as the reference level for men (category 2), an intake of ≥ 5,000 mg sodium (category 5) was associated with a 12% increased risk of overall mortality (HR = 1.12, 95% CIs: 1.06, 1.18) with adjusted ARDs at 16 years of follow-up of 0.84% (95% CI, 0.40% to 1.37%) (Additional file 1: Table S3). An intake of < 2,000 mg (category 1) was also associated with a 4% increased mortality (HR = 1.04, 95% CI: 1.01, 1.06). When compared with the category 2 reference level in women of 1,400- < 2,200 mg, an intake of ≥ 4,000 mg sodium (category 5) was associated with a 14% increased risk of overall mortality (HR = 1.14, 95% CI: 1.06, 1.23), with adjusted ARDs at 16 years of follow-up of 0.73% (95% CI: 0.20% to 1.24%) (Additional file 1: Table S4).

**Table 2:** Risk of overall and cause-specific mortality associated with daily sodium intake in the NIH-AARP study^a^

Mortality cause	Sodium intake (≥ 2,000 mg/d)						*P* value (Non-linear)^b^	*P* for interaction^b^
	**Quintile 1 (*n* = 58,686)**	**Quintile 2 (*n* = 58,687)**	**Quintile 3 (*n* = 58,687)**	**Quintile 4 (*n* = 58,687)**	**Quintile 5 (*n* = 58,686)**	***P* for trend (linear)**^**b**^		
	**HR (95% CI)**	**HR (95% CI)**	**HR (95% CI)**	**HR (95% CI)**	**HR (95% CI)**			
**Overall mortality**								
Men	1.00	0.96 (0.93, 0.99)	0.97 (0.94, 1.01)	1.00 (0.96, 1.03)	1.06 (1.02, 1.09)	0.0002^***^	< 0.0001^***^	0.017^*^
Women	1.00	0.99 (0.94, 1.04)	0.97 (0.92, 1.02)	1.04 (0.99, 1.10)	1.10 (1.04, 1.16)	< 0.0001^***^	< 0.0001^***^	
**CVD**								
Men	1.00	0.91 (0.86, 0.97)	0.94 (0.88, 1.00)	0.99 (0.93, 1.05)	1.07 (1.00, 1.14)	0.001^**^	0.0002^***^	0.055
Women	1.00	1.00 (0.90, 1.11)	0.97 (0.88, 1.08)	1.08 (0.97, 1.20)	1.21 (1.09, 1.34)	< 0.0001^***^	0.01^*^	
**Heart disease**								
Men	1.00	0.93 (0.87, 0.99)	0.95 (0.89, 1.02)	1.02 (0.95, 1.09)	1.08 (1.01, 1.15)	0.001^**^	0.0002^***^	0.019^*^
Women	1.00	1.02 (0.90, 1.15)	1.02 (0.90, 1.14)	1.12 (1.00, 1.27)	1.27 (1.13, 1.43)	< 0.0001^***^	0.03^*^	
**Stroke**								
Men	1.00	0.83 (0.71, 0.98)	0.87 (0.74, 1.02)	0.87 (0.74, 1.02)	1.02 (0.87, 1.20)	0.59	0.46	0.60
Women	1.00	0.96 (0.77. 1.19)	0.88 (0.70, 1.10)	1.02 (0.82, 1.27)	1.06 (0.85, 1.33)	0.36	0.25	
**Cancer**								
Men	1.00	1.00 (0.95, 1.05)	1.02 (0.96, 1.07)	1.04 (0.98, 1.10)	1.06 (1.00, 1.12)	0.024^*^	0.31	0.56
Women	1.00	0.92 (0.85, 1.00)	1.03 (0.94, 1.12)	1.02 (0.93, 1.11)	1.00 (0.92, 1.10)	0.27	0.12	
**Respiratory disease**								
Men	1.00	1.06 (0.94, 1.20)	1.01 (0.89, 1.15)	1.01 (0.89, 1.16)	1.13 (0.99, 1.30)	0.20	0.65	0.055
Women	1.00	1.13 (0.96, 1.33)	1.00 (0.84, 1.19)	1.06 (0.89, 1.26)	1.18 (0.99, 1.41)	0.19	0.39	
**Infectious disease**								
Men	1.00	0.97 (0.82, 1.14)	0.81 (0.68, 0.97)	0.85 (0.71, 1.01)	0.95 (0.79, 1.13)	0.25	0.37	0.23
Women	1.00	0.99 (0.77, 1.28)	0.91 (0.70, 1.18)	1.03 (0.79, 1.33)	1.01 (0.77, 1.32)	0.83	0.14	
**Injury and accidents**								
Men	1.00	1.04 (0.89, 1.22)	0.92 (0.77, 1.09)	1.00 (0.84, 1.18)	0.99 (0.83, 1.18)	0.75	0.01^*^	0.13
Women	1.00	1.13 (0.82, 1.54)	0.93 (0.67, 1.29)	0.99 (0.71, 1.38)	1.14 (0.82, 1.59)	0.69	0.30	
**Other causes**								
Men	1.00	0.93 (0.86, 1.00)	0.99 (0.92, 1.07)	0.95 (0.88, 1.02)	1.05 (0.97, 1.13)	0.12	0.0002^***^	0.98
Women	1.00	1.00 (0.89, 1.12)	0.86 (0.76, 0.97)	1.02 (0.91, 1.14)	1.09 (0.97, 1.23)	0.053	0.001^**^	

^a^Multivariable analyses were adjusted for age at baseline, BMI, alcohol consumption, smoking status (never, former, current or missing), physical activity, race or ethnic group, education, marital status, diabetes (yes vs. no), health status, vitamin supplement use, total energy intake, and Healthy Eating Index 2015 (HEI-2015) score excluding the sodium component. *P* for trend (linear) was calculated based on statistical significance of the coefficient of the category variable (the ordinal value of the quintile). *P* value (Non-linear) was derived from Fig. 1a. *P* for interaction was calculated based on the likelihood test to evaluate the statistical significance of the cross-product term (quintiles of sodium and sex) entered into the Cox proportional hazard regression

^b^^*^p < 0.05, ^**^p < 0.01, ^***^p < 0.00093 (the Bonferroni corrected threshold)

Although with wider confidence intervals, there was also clear evidence of non-linear associations of dietary sodium intake with risk of mortality from CVD and heart disease in men (both P_nonlinearity_ = 0.0002), and mortality from other causes combined in both men and women (P_nonlinearity_ ≤ 0.001) (Fig. 1a). In men, the HRs for CVD mortality was 1.07 (95% CI: 1.00, 1.14) in the fifth versus the lowest quintile; 1.08 (95% CI: 1.01, 1.15) for heart disease mortality (Table 2). Evidence to suggest possible nonlinearity was observed between dietary sodium and mortality from CVD, heart disease mortality in women (P_nonlinearity_ = 0.01 and 0.03, respectively; all *P* value < 0.0001; Fig. 1a). For CVD and heart disease mortality in women, the HRs were 1.21 (95% CI: 1.09, 1.34) and 1.27 (95% CI: 1.13, 1.43) for sodium intake in the fifth category versus the first quintile, respectively (Table 2).

### Dietary potassium and overall and cause-specific mortality

From the multivariable-adjusted models, the associations between dietary potassium and mortality outcomes are demonstrated in Fig. 1b, and minimally adjusted and parsimoniously adjusted associations are presented in the supplemental data (Additional file 1: Figures S2 and S5). The supplemental data showed the HRs and their 95% CIs of the multivariable-adjusted cubic-restricted splines (Additional file 1: Tables S3 and S4). Table 3 and the supplemental data (Additional file 1: Table S6) present the estimated HRs and their corresponding 95% CIs based on quintiles, per 500 mg or per SD change in potassium intake for the associations with the risk of overall mortality and cause-specific mortality. Restricted cubic splines showed statistically significant curvilinear inverse associations between potassium intake and overall mortality in men and women (P_nonlinearity_ < 0.0001). The overall mortality association for men was initially steeper and plateaued at approximately 4000 mg/d, while the association for women appeared more linear (Fig. 1b). In both men and women, participants in quintiles 2–5 experienced a significantly lower risk of overall mortality than those in the lowest quintile, and a stronger inverse association was noted in women (fifth versus first quintile, HR for men = 0.96, 95% CI: 0.92, 0.99; HR for women = 0.82, 95% CI: 0.79, 0.86; all P_trend_ ≤ 0.01; *P* for interaction = 0.0006) (Table 3). Adjusted ARDs at 16 years of follow-up were -0.39% (95% CI: -0.65% to -0.14%) and -0.49% (95% CI: -0.75% to -0.48%) for men and women, respectively (fifth versus first category) (Additional file 1: Tables S3 and S4).

**Table 3:** Risk of overall and cause-specific mortality associated with daily potassium intake in the NIH-AARP study^a^

Mortality cause	Potassium intake						*P* value (Non-linear) ^b^	*P* for interaction^b^
	**Quintile 1 (*n* = 83,220)**	**Quintile 2 (*n* = 83,221)**	**Quintile 3 (*n* = 83,221)**	**Quintile 4 (*n* = 83,221)**	**Quintile 5 (*n* = 83,221)**	***P* for trend (linear) **^**b**^		
	**HR (95% CI)**	**HR (95% CI)**	**HR (95% CI)**	**HR (95% CI)**	**HR (95% CI)**			
**Overall mortality**								
Men	1.00	0.93 (0.90, 0.95)	0.92 (0.89, 0.94)	0.94 (0.91, 0.96)	0.96 (0.92, 0.99)	0.01^*^	< 0.0001^***^	0.0006^***^
Women	1.00	0.89 (0.86, 0.93)	0.86 (0.82, 0.89)	0.86 (0.82, 0.89)	0.82 (0.79, 0.86)	< 0.0001^***^	< 0.0001^***^	
**CVD**								
Men	1.00	0.94 (0.89, 0.98)	0.95 (0.90, 1.01)	0.97 (0.91, 1.03)	1.02 (0.95, 1.09)	0.53	< 0.0001^***^	0.0006^***^
Women	1.00	0.87 (0.80, 0.94)	0.82 (0.75, 0.89)	0.82 (0.76, 0.89)	0.79 (0.72, 0.87)	< 0.0001^***^	< 0.0001^***^	
**Heart disease**								
Men	1.00	0.94 (0.89, 0.99)	0.95 (0.90, 1.01)	0.97 (0.91, 1.04)	1.03 (0.96, 1.10)	0.41	< 0.0001^***^	0.0028^**^
Women	1.00	0.87 (0.79, 0.95)	0.82 (0.75, 0.90)	0.81 (0.74, 0.90)	0.80 (0.73, 0.89)	0.0001^***^	0.0006^***^	
**Stroke**								
Men	1.00	0.92 (0.81, 1.04)	0.95 (0.83, 1.09)	0.93 (0.80, 1.08)	0.92 (0.78, 1.09)	0.43	0.002^**^	0.30
Women	1.00	0.84 (0.70, 1.00)	0.80 (0.67, 0.96)	0.85 (0.71, 1.02)	0.79 (0.65, 0.96)	0.074	0.09	
**Cancer**								
Men	1.00	0.96 (0.92, 1.00)	0.93 (0.88, 0.97)	0.95 (0.90, 1.00)	0.96 (0.90, 1.02)	0.07	0.06	0.64
Women	1.00	0.95 (0.88, 1.02)	0.94 (0.88, 1.01)	0.94 (0.88, 1.01)	0.93 (0.86, 1.01)	0.15	0.10	
**Respiratory disease**								
Men	1.00	0.83 (0.75, 0.92)	0.83 (0.75, 0.93)	0.89 (0.79, 1.01)	0.82 (0.71, 0.94)	0.01	0.09	0.43
Women	1.00	0.84 (0.74, 0.96)	0.70 (0.62, 0.81)	0.76 (0.66, 0.87)	0.64 (0.55, 0.75)	< 0.0001^***^	0.07	
**Infectious disease**								
Men	1.00	0.98 (0.86, 1.13)	0.94 (0.80, 1.09)	1.05 (0.89, 1.24)	1.01 (0.84, 1.22)	0.68	0.26	0.51
Women	1.00	0.92 (0.75, 1.14)	0.92 (0.75, 1.14)	0.91 (0.73, 1.12)	0.77 (0.61, 0.97)	0.04^*^	0.35	
**Injury and accidents**								
Men	1.00	0.94 (0.82, 1.08)	0.89 (0.77, 1.03)	0.88 (0.75, 1.03)	0.85 (0.71, 1.03)	0.06	0.005^**^	0.91
Women	1.00	0.87 (0.67, 1.13)	0.89 (0.68, 1.16)	0.71 (0.54, 0.94)	0.77 (0.58, 1.03)	0.04^*^	0.27	
**Other causes**								
Men	1.00	0.88 (0.83, 0.94)	0.87 (0.82, 0.93)	0.88 (0.82, 0.94)	0.92 (0.85, 0.99)	0.03^*^	< 0.0001^***^	0.045^*^
Women	1.00	0.86 (0.79, 0.95)	0.83 (0.75, 0.90)	0.81 (0.74, 0.89)	0.79 (0.72, 0.88)	< 0.0001^***^	< 0.0001^***^	

^a^Multivariable analyses were adjusted for age at baseline, BMI, alcohol consumption, smoking status (never, former, current or missing), physical activity, race or ethnic group, education, marital status, diabetes (yes vs. no), health status, vitamin supplement use, total energy intake and Healthy Eating Index 2015 (HEI-2015 components for sodium, seafood and plant protein, saturated fat, fatty acids and refined grains). *P* for trend (linear) was calculated based on statistical significance of the coefficient of the category variable (the ordinal value of the quintile). *P* value (Non-linear) was derived from Fig. 1b. *P* for interaction was calculated based on the likelihood test to evaluate the statistical significance of the cross-product term (quintiles of potassium and sex) entered into the Cox proportional hazard regression

^b^^*^p < 0.05, ^**^p < 0.01, ^***^p < 0.00093 (the Bonferroni corrected threshold)

For mortality from heart disease, and other causes combined, similar patterns of non-linear associations of potassium intake were observed for both men and women, but with wider confidence intervals (P_nonlinearity_ ≤ 0.0006; Fig. 1b). Higher intakes of dietary potassium were associated with a greater significant risk reduction for mortality from CVD and heart disease for women (fifth versus first quintile, HR for CVD mortality = 0.79, 95% CI: 0.72, 0.87; HR for heart disease mortality = 0.80, 95% CI: 0.73, 0.89), but not for men (fifth versus first quintile, HR for CVD mortality = 1.02, 95% CI: 0.95, 1.09; HR for heart disease mortality = 1.03, 95% CI: 0.96, 1.10) (P for interaction = 0.0028; Table 3).

A U-shaped association of potassium intake with stroke mortality was suggested for men, with a minimum risk found at approximately 4,000 mg/d (P_nonlinearity_ = 0.002, Fig. 1b). There were linear inverse associations for respiratory disease mortality in men and women and infectious disease mortality in women (all *P*-values ≤ 0.04). Participants in quintiles 2–5 had a lower risk of respiratory disease mortality than those in the lowest quintile (fifth versus first quintile, HR for men = 0.82, 95% CI: 0.71, 0.94; HR for women = 0.64, 95% CI: 0.55, 0.75; Table 3). Higher potassium intake was associated with a lower risk of infectious disease mortality for women (fifth versus first quintile, HR = 0.77, 95% CI: 0.61, 0.97), but not for men (HR = 1.01, 95% CI: 0.84, 1.22) (Table 3).

### Dietary sodium–potassium ratio and overall and cause-specific mortality

Figure 1c shows the dose–response multivariable-adjusted associations between sodium–potassium ratio and mortality outcomes, and the HRs and their corresponding 95% CIs of the cubic-restricted splines are shown in the supplemental data (Additional file 1: Tables S3 and S4); minimally adjusted and parsimoniously adjusted associations are reported in Additional file 1: Figures S3 and S6. Table 4 and the supplemental data (Additional file 1: Table S7) present the estimated HRs and their corresponding 95% CIs based on quintiles and per SD change in sodium–potassium ratio for the associations with the risk of overall mortality and cause-specific mortality. Among men, curvilinear associations for the dietary sodium–potassium ratio were observed for overall and CVD mortality (P_nonlinearity_ values ≤ 0.0003), and heart disease mortality (P_nonlinearity_ = 0.002). With the lowest quintile as the reference group, only the fifth quintile of the sodium–potassium ratio showed significantly increased risk of overall and CVD mortality for men (HR for overall mortality = 1.09, 95% CI: 1.05, 1.13; HR for CVD mortality = 1.08, 95% CI: 1.00, 1.15; both P_trend_ < 0.05) (Table 5).

**Table 4:** Risk of overall and cause-specific mortality associated with daily intake of the sodium–potassium ratio in the NIH-AARP study^a^

Mortality cause	Sodium–potassium ratio (sodium ≥ 2,000 mg/d)						*P* value (Non-linear)^b^	*P* for interaction^b^
	**Quintile 1 (*n* = 58,686)**	**Quintile 2 (*n* = 58,687)**	**Quintile 3 (*n* = 58,687)**	**Quintile 4 (*n* = 58,687)**	**Quintile 5 (*n* = 58,686)**	***P* for trend (linear)**^b^		
	**HR (95% CI)**	**HR (95% CI)**	**HR (95% CI)**	**HR (95% CI)**	**HR (95% CI)**			
**Overall mortality**								
Men	1.00	1.01 (0.97, 1.04)	1.00 (0.96, 1.03)	1.01 (0.97, 1.04)	1.09 (1.05, 1.13)	< 0.0001^***^	< 0.0001^***^	< 0.0001^***^
Women	1.00	1.03 (0.98, 1.07)	1.07 (1.02, 1.13)	1.11 (1.05, 1.17)	1.23 (1.16, 1.31)	< 0.0001^***^	0.04^*^	
**CVD**								
Men	1.00	0.95 (0.89, 1.01)	0.97 (0.91, 1.03)	0.94 (0.88, 1.01)	1.08 (1.00, 1.15)	0.048^*^	0.0003^***^	< 0.0001^***^
Women	1.00	1.00 (0.92, 1.10)	1.16 (1.05, 1.28)	1.14 (1.02, 1.26)	1.34 (1.19, 1.50)	< 0.0001^***^	0.12	
**Heart disease**								
Men	1.00	0.97 (0.90, 1.03)	0.97 (0.91, 1.04)	0.96 (0.89, 1.03)	1.07 (0.99, 1.15)	0.11	0.002^**^	< 0.0001^***^
Women	1.00	1.03 (0.93, 1.15)	1.14 (1.01, 1.27)	1.19 (1.05, 1.34)	1.38 (1.22, 1.58)	< 0.0001^***^	0.16	
**Stroke**								
Men	1.00	0.87 (0.74, 1.03)	0.99 (0.84, 1.16)	0.87 (0.73, 1.03)	1.12 (0.94, 1.33)	0.25	0.13	0.27
Women	1.00	0.87 (0.72, 1.07)	1.20 (0.98, 1.47)	1.00 (0.79, 1.26)	1.21 (0.94, 1.55)	0.09	0.19	
**Cancer**								
Men	1.00	1.06 (1.00, 1.12)	1.05 (0.99, 1.11)	1.05 (0.99, 1.12)	1.07 (1.00, 1.13)	0.13	0.94	0.71
Women	1.00	1.01 (0.93, 1.09)	1.01 (0.93, 1.10)	1.03 (0.94, 1.13)	1.09 (0.99, 1.21)	0.10	0.44	
**Respiratory disease**								
Men	1.00	0.86 (0.75, 0.99)	0.91 (0.79, 1.05)	0.98 (0.85, 1.12)	1.13 (0.98, 1.30)	0.009^**^	0.09	0.17
Women	1.00	1.18 (1.00, 1.39)	1.19 (1.00, 1.42)	1.38 (1.15, 1.66)	1.50 (1.24, 1.83)	< 0.0001^***^	0.32	
**Infectious disease**								
Men	1.00	1.06 (0.89, 1.27)	0.99 (0.82, 1.18)	0.89 (0.73, 1.07)	0.94 (0.77, 1.15)	0.17	0.07	0.07
Women	1.00	1.14 (0.90, 1.44)	1.04 (0.80, 1.35)	1.29 (0.99, 1.69)	1.46 (1.09, 1.96)	0.01^*^	0.61	
**Injury and accidents**								
Men	1.00	1.09 (0.92, 1.29)	0.94 (0.78, 1.12)	1.10 (0.92, 1.32)	1.09 (0.90, 1.32)	0.40	0.12	0.29
Women	1.00	1.29 (0.98, 1.70)	1.15 (0.84, 1.58)	1.30 (0.93, 1.81)	1.60 (1.11, 2.31)	0.03^*^	0.61	
**Other causes**								
Men	1.00	1.03 (0.96, 1.11)	0.99 (0.92, 1.08)	1.04 (0.96, 1.12)	1.18 (1.08, 1.28)	0.0004^***^	0.06	0.09
Women	1.00	0.98 (0.89, 1.09)	1.03 (0.93, 1.15)	1.08 (0.96, 1.22)	1.18 (1.04, 1.34)	0.006^**^	0.33	

^a^Multivariable analyses were adjusted for age at baseline, BMI, alcohol consumption, smoking status (never, former, current or missing), physical activity, race or ethnic group, education, marital status, diabetes (yes vs. no), health status, vitamin supplement use, total energy intake, and Healthy Eating Index 2015 (HEI-2015 components for seafood and plant protein, saturated fat, fatty acids and refined grains). P for trend (linear) was calculated based on statistical significance of the coefficient of the category variable (the ordinal value of the quintile). *P* value (Non-linear) was derived from Fig. 1c. *P* for interaction was calculated based on the likelihood test to evaluate the statistical significance of the cross-product term (quintiles of sodium–potassium ratio and sex) entered into the Cox proportional hazard regression

^b^^*^p < 0.05, ^**^p < 0.01, ^***^p < 0.00093 (the Bonferroni corrected threshold)

**Table 5:** Risk of overall mortality associated with intakes of sodium, potassium and sodium–potassium ratio stratified across racial/ethnic groups among men and women^a^

	**Men**			**Women**		
	**All events/person-years**	**HR (95% CI)**	***P* value for interaction**	**All events/person-years**	**HR (95% CI)**	***P* value for interaction**
**Sodium intake (≥ 2,000 mg/d)**						
Non-Hispanic white	7,438/474,338	1.05 (1.01, 1.09)		3,509/303,931	1.09 (1.03, 1.15)	
Hispanic, total minority racial and other ethnic group	333/24,343	1.14 (0.96, 1.35)	0.092	179/16,156	1.21 (0.95, 1.54)	0.59
Non-Hispanic black	194/10,311	1.21 (0.99, 1.47)	0.42	181/12,695	1.16 (0.95, 1.43)	0.56
**Potassium intake**						
Non-Hispanic white	6,209/427,105	0.96 (0.93, 1.00)		6,997/681,326	0.81 (0.77, 0.85)	
Hispanic, total minority racial and other ethnic group	349/25,824	0.88 (0.75, 1.03)	0.64	411/38,593	0.92 (0.74, 1.13)	0.017
Non-Hispanic black	163/10,240	0.87 (0.71, 1.07)	0.99	310/27,828	0.99 (0.82, 1.19)	0.11
**Sodium–potassium ratio (sodium ≥ 2,000 mg/d)**						
Non-Hispanic white	8,691/551,192	1.09 (1.04, 1.13)		2,672/205,047	1.27 (1.19, 1.35)	
Hispanic, total minority racial and other ethnic group	371/27,948	1.15 (0.96, 1.39)	0.89	116/11,957	1.11 (0.83, 1.47)	0.25
Non-Hispanic black	396/22,678	1.39 (1.11, 1.75)	0.52	250/17,448	1.02 (0.81, 1.29)	0.067

^a^Hazard ratios (HRs) and their 95% confidence intervals (CIs) for mortality comparing the highest versus the lowest quintile. Multivariable analyses were adjusted for age at baseline, BMI, alcohol consumption, smoking status (never, former, current or missing), physical activity, education, marital status, diabetes (yes vs. no), health status, vitamin supplement use, and total energy intake. For sodium intake, models were additionally adjusted for Healthy Eating Index 2015 (HEI-2015) score excluding the sodium component; for potassium intake and the sodium–potassium ratio, models were additionally adjusted for HEI-2015 components for sodium (potassium model only), seafood and plant protein, saturated fat, fatty acids and refined grains. For women, the risk estimates were additionally adjusted for postmenopausal hormone therapy (yes vs. no). *P* for interaction was examined by the likelihood ratio test, entering the cross-product term of exposure factors (categories) and the stratification variables (Racial/Ethnic groups), all as ordinal variables, to the Cox proportional hazard regression model

We observed linear associations of dietary sodium–potassium ratio with overall mortality in women (fifth versus first quintile, HR = 1.23, 95% CI: 1.16, 1.31), and with mortality from respiratory disease in women (fifth versus first quintile, HR = 1.50, 95% CI: 1.24, 1.83) and other causes combined in men and women (Table 4). We also found that the linear associations of the sodium–potassium ratio were steeper for CVD and heart disease mortality in women than in men (fifth versus first quintile, HR for CVD mortality = 1.34, 95% CI: 1.19, 1.50; HR for heart disease mortality = 1.38, 95% CI: 1.22, 1.58) (both P_trend_ < 0.0001; both *P* for interaction < 0.0001; Table 4).

### Dietary sodium, potassium, sodium–potassium ratio and overall mortality in cohort subgroups

**Fig. 2:**
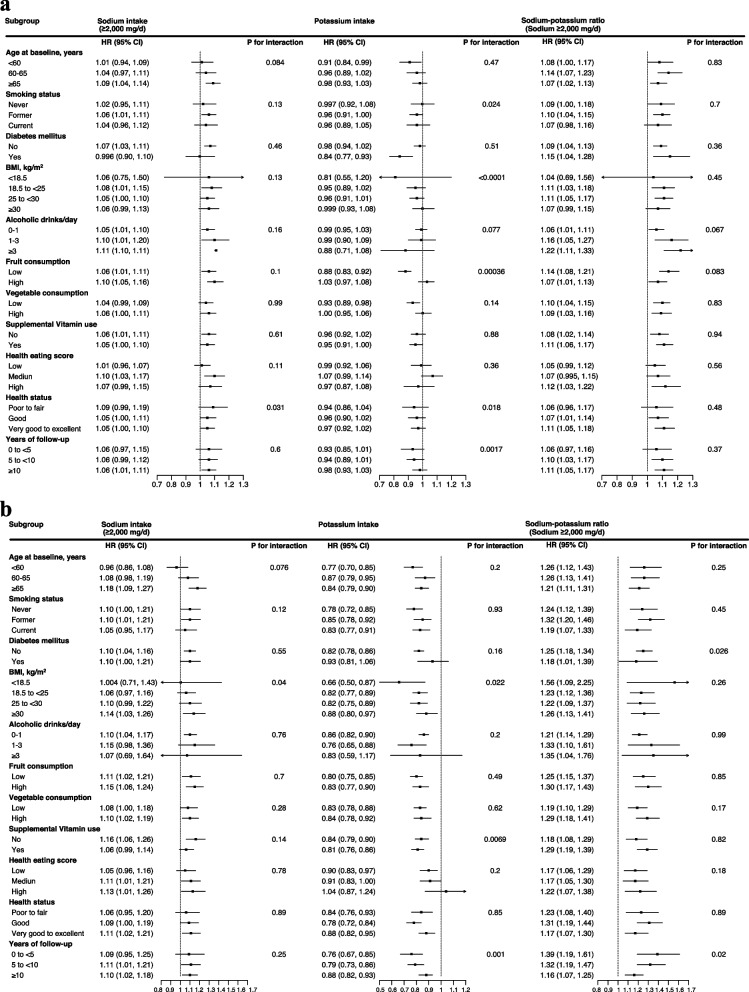
**a** Associations Between Intakes of Sodium, Potassium, Sodium–Potassium Ratio and Overall and Cause-specific Mortality in Multivariable-Adjusted Models Stratified by Selected Characteristics Among Men. Hazard ratios (HRs) and their 95% confidence intervals (CIs) for overall mortality comparing the highest versus the lowest quintile. Multivariable analyses were adjusted for age at baseline, BMI, alcohol consumption, smoking status (never, former, current or missing), physical activity, race or ethnic group, education, marital status, diabetes (yes vs. no), health status, vitamin supplement use, and total energy intake. For sodium intake, models were further adjusted for Healthy Eating Index 2015 (HEI-2015) score excluding the sodium component; for potassium intake and the sodium–potassium ratio, models were additionally adjusted for HEI-2015 components for sodium (potassium model only), seafood and plant protein, saturated fat, fatty acids and refined grains. *P* for interaction was examined by the likelihood ratio test, entering the cross-product term of exposure factors (categories) and the stratification variables (categorized as shown), all as ordinal variables, to the Cox proportional hazard regression model. **b** Associations Between Intakes of Sodium, Potassium, Sodium–Potassium Ratio and Overall and Cause-specific Mortality in Multivariable-Adjusted Models Stratified by Selected Characteristics Among Women. Hazard ratios (HRs) and their 95% confidence intervals (CIs) for overall mortality comparing the highest versus the lowest quintile. Multivariable analyses were adjusted for age at baseline, BMI, alcohol consumption, smoking status (never, former, current or missing), physical activity, race or ethnic group, education, marital status, diabetes (yes vs. no), health status, postmenopausal hormone therapy (yes vs. no), vitamin supplement use, and total energy intake. For sodium intake, models were additionally adjusted for Healthy Eating Index 2015 (HEI-2015) score excluding the sodium component; for potassium intake and the sodium–potassium ratio, models were additionally adjusted for HEI-2015 components for sodium (potassium model only), seafood and plant protein, saturated fat, fatty acids and refined grains. *P* for interaction was examined by the likelihood ratio test, entering the cross-product term of exposure factors (categories) and the stratification variables (categorized as shown), all as ordinal variables, to the Cox proportional hazard regression model

Based on the multivariable-adjusted models in men and women, Fig. 2a, b provides findings from stratified analyses of cohort subgroups. The overall mortality associations with dietary intake of sodium, potassium and sodium–potassium ratio were generally similar across population subgroups of age at baseline, smoking status, diabetes, alcohol intake, vegetable consumption, supplemental vitamin use, HEI-2015, self-reported health status and years of follow-up. However, the association between potassium intake and overall mortality was stronger among men with lower BMI (*P* for interaction < 0.0001, Fig. 2a). Additionally, the association between potassium intake and overall mortality in men was modified by fruit consumption, with the association being significant in lower fruit consumers (fifth versus first category, HR = 0.88, 95% CI: 0.83, 0.92), but not in high fruit consumers (HR = 1.03, 95% CI: 0.97, 1.08) (*P* for interaction = 0.00036). Regarding sex differences, the associations between potassium intake, the sodium–potassium ratio and risk of overall and CVD mortality were statistically significantly different (*P* for interaction ≤ 0.0006 for overall and CVD mortality, Tables 3 and 4).

In the stratified analyses for both men and women, the associations between intakes of sodium, potassium and sodium–potassium ratio and risk of overall mortality were not statistically significantly different across racial/ethnic groups (Table 5). Compared with the referent (lowest quintile) category, a higher intake of daily sodium was associated with a 5% and 14% increased risk of overall mortality in men, and a 9% and 21% increased risk in women, among non-Hispanic white individuals and the combined racial/ethnic participants, respectively; although stronger associations were seen for the combined racial/ethnic group, they did not reach a statistically significant threshold (P_interaction_ = 0.092 and 0.59, respectively). We observed stronger potassium-mortality associations among non-Hispanic white participants in women, with a 19% and 8% decreased risk (fifth versus first quintile) in the non-Hispanic white participants than the combined racial/ethnic group (P_interaction_ = 0.017, respectively). Participants in a higher category of sodium–potassium intake ratio experienced a 9% and 15% increased risk of overall mortality in men, and a 27% and 11% increased risk in women (fifth versus first quintile), among the non-Hispanic white individuals and the combined racial/ethnic participants, respectively (P_interaction_ = 0.89 and 0.25, respectively).

### Dietary sodium, potassium, sodium–potassium ratio and overall mortality in sensitivity analyses

The primary findings of associations between dietary intake of sodium, potassium, sodium–potassium ratio and risk of overall and cause-specific mortality did not change materially in the sensitivity analyses which excluded the first five years of follow-up (Additional file 1: Figures S7-S9), or participants who reported a history of diabetes at study entry (Additional file 1: Figures S10-S12), or participants who reported poor to fair health status or unknown health status at study entry (Additional file 1: Figures S13-S15), or additionally adjusted for family income or dietary potassium intake in the sodium-mortality models (Additional file 1: Figures S16-S19).

### Systematic review and meta-analysis

Overall, our initial search yielded 3,318 articles after removing duplicate studies. After evaluating the titles and abstracts, 93 articles were reviewed in full, and 33 studies (34 with the current study) were included in the meta-analysis (Additional file 1: Tables S8-S10). The supplemental data depicts the study selection process (Additional file 1: Figure S20), and illustrates characteristics of the individual studies included in the meta-analysis (Additional file 1: Table S10). Based on the Newcastle–Ottawa quality assessment scale, 16 studies (including the current study) reached a score of at least seven, reflecting a low risk of bias (Additional file 1: Table S11).

**Fig. 3:**
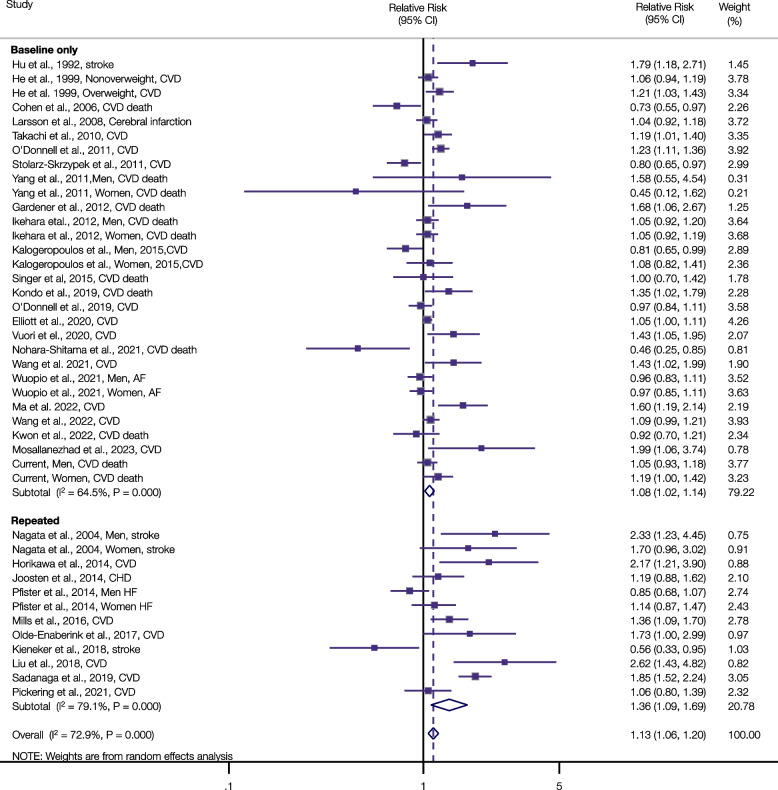
Association of Sodium Intake with Cardiovascular Disease Risk for the Highest Versus Lowest Intake Category Using Random-Effects Meta-Analysis According to Baseline Only and Repeated Assessment of Sodium Intake (*P* for interaction = 0.027). Squares represent study-specific relative risks, with their areas being proportional to the specific-study weight in the meta-analysis. Horizontal lines denote 95% Cis. I^2^ refers to the proportion of heterogeneity among studies. Abbreviations: AF = atrial fibrillation; CHD = coronary heart disease; CI = confidence interval; CVD = cardiovascular disease; HF = heart failure

A total of 34 studies of sodium intake and risk of incident CVD and CVD mortality comprised a total of 42 risk estimates, 2,085,904 participants (dietary questionnaires: 1,014,282 participants; urinary measurement: 1,071,622) and 80,085 CVD events (dietary questionnaires: 44,530 participants; urinary measurement: 35,555). The pooled RR (95% CI) was 1.13 (1.06, 1.20) for the highest versus lowest category of sodium intake. We found no indication of publication bias for the association between sodium intake and risk of CVD (including CVD mortality) (*P* value for Egger’s test = 0.10, Additional file 1: Fig. S21), but observed considerable between-study heterogeneity (I^2^ = 72.9%, Fig. 3), although no single study was overly influential in this regard (Additional file 1: Fig. S22). In the meta-analysis of predefined subgroups, there were no statistically significant interactions, with the exception of dietary assessment (P_interaction_ = 0.027), where the positive association between sodium intake and CVD risk was stronger among studies with repeated assessments of sodium intake in the follow-up period (pooled RR = 1.36, 95% CI: 1.09, 1.69) than among those with only one baseline assessment estimate of sodium intake (pooled RR = 1.08, 95% CI: 1.02, 1.14) (Fig. 3 and Additional file 1: Table S12). Additionally, all subgroup data were provided in greater than 10 studies/risk estimates in accordance with the Cochrane recommendations (https://training.cochrane.org/handbook/current/chapter-10), with the exception of sex-specific analyses: 9 risk estimates for men and 7 risk estimates for women.

**Fig. 4:**
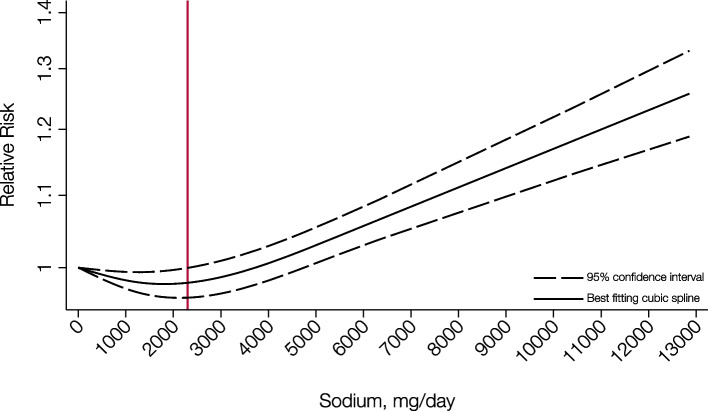
Association Between Sodium Intake and Risk of Cardiovascular Disease Using a Cubic-Restricted Spline Model in the Dose–Response Meta-Analysis Based on 29 Published Studies. The solid line represents the relative risk of cardiovascular disease according to sodium intake, and the dashed lines denote the 95% confidence intervals (P_nonlinearity_ < 0.001). The spline was computed on the basis of three knots selected at 5^th^, 50^th^, 95^th^ percentiles of sodium intake, and the red line depicts the sodium intake of 2,300 mg/day. The analysis included 2,056,043 participants from 29 cohort studies with 66,526 CVD events

In the meta-analysis of 29 cohort studies with 2,056,043 participants and 66,526 CVD events, we conducted a 3-knot restricted cubic spline regression analysis which yielded similar findings, with the nadir of CVD risk (including CVD mortality) in the daily sodium intake range of 1,700 mg to 2,300 mg, and a striking CVD risk excess for individuals with sodium intake greater than 5,000 mg/day (P_nonlinearity_ < 0.001, Fig. 4).

## Discussion

We investigated sex-specific associations between dietary intake of sodium and potassium, the sodium–potassium ratio, and the risk of overall and cause-specific mortality in a large U.S. prospective cohort study followed for an average of 15.5 years. Participants with higher sodium intake, a higher sodium–potassium ratio, or lower potassium intake experienced significantly increased risk of CVD and overall mortality, with women having stronger potassium-mortality and sodium–potassium ratio-mortality associations than men. Findings from the updated meta-analysis of 34 prospective cohort studies provided additional evidence that participants with higher sodium intake had significantly increased risk of CVD incidence and CVD mortality. However, we found some evidence of heterogeneity between studies (I^2^ = 72.9%), likely due to dietary assessment frequency, whereby studies with repeated sodium measurements showed a stronger positive sodium-CVD association than studies with only a baseline measurement. The meta-analysis found similar summary RRs of the sodium-CVD associations regardless of whether sodium intake was estimated from dietary questionnaires or urinary measurements.

### Previous studies

Randomized controlled trials have demonstrated effects of sodium intake on increased blood pressure, as well as associations between high blood pressure and risk of CVD incidence and CVD mortality [1, 7–10, 34, 35]. However, findings from prior studies of the associations between sodium intake and overall and cause-specific mortality (including CVD) remain largely conflicting. In the large international cohort of the PURE Study that included more than 100,000 participants with measurements of morning fasting urine samples, after a median follow-up period of 8.2 years the investigators showed a J-shaped association between sodium intake and risk of mortality and CVD events, with the lowest risk observed for a moderate sodium intake of 3–5 g/day along with greater potassium intake [18]. In the U.K. Biobank study of more than 390,000 participants with estimated 24-h urinary sodium excretion and a median duration of 5.9 years of observation, the investigators showed no significant association between sodium intake and CVD events, but found a J-shaped relationship with all-cause mortality [6].

In the present prospective cohort with more than 400,000 men and women in the U.S., we observed positive associations between higher sodium intake and increased risk of overall mortality and CVD mortality, with the findings pointing to possible linear associations for cancer and respiratory disease mortality. In the most recent prospective cohort study among 10,709 participants in which sodium excretion was measured using repeated 24-h urine samples (known to be a more accurate estimate of sodium intake), the investigators showed that higher intake was significantly linearly associated with an increased risk of CVD within a range of daily sodium intake between 2,000 and 6,000 mg [11], findings essentially consistent with the present data showing a linear positive CVD mortality association in the same intake range. In addition, our updated meta-analysis showed that sodium-CVD associations remain stable with similar summary RRs in the studies with sodium calculated from dietary questionnaire or those with urinary measurements. It is reassuring that lines of evidence from sodium intake and 24-h sodium excretion studies show similar results; we therefore suggest these various studies should be taken into account for the development of sodium intake guidelines. Although there appeared to be a J-shaped association between sodium intake and mortality risk, we cannot draw a firm conclusion regarding the elevated risk between lower sodium intake and mortality, given the wide 95% confidence intervals for intake < 2,000 mg/d. Additionally, we need to interpret this finding cautiously, as a lower sodium intake may reflect poor intakes of all nutrients; i.e., a sign of anorexia, sub-clinical illness, or unidentified comorbidity [27]. Further studies are warranted to examine the extremely low sodium intake-mortality risk association.

Previous data on sodium intake and mortality focusing on sex differences are sparse, and results from our study suggest that associations between sodium intake and overall and CVD mortality appear stronger for women than for men, with the risk patterns being consistent with data from previous prospective cohort studies [11, 36]. At present, it has been suggested that individuals “consume less than 2,300 mg per day of sodium (based on the Tolerable Upper Intake Level for individuals ages 14 years and older)” according to the dietary recommendations from the Scientific Report of the 2020 Dietary Guidelines Advisory Committee [37]. However, even with the significant difference in sodium intake between men and women, sex-specific risk was not taken into account. The present findings of substantially stronger sodium- and sodium–potassium ratio-mortality associations in women compared with men should be reexamined in other prospective studies, and if replicated, may provide additional evidence relevant to health-related dietary sodium guidelines.

### Plausible biological mechanisms

Accumulated evidence and plausible physiological mechanisms have supported a causal association between higher sodium intake and increased mortality, including the adverse consequences of increased sodium load on multiple organs and tissues through direct and indirect blood pressure-mediated effects, and relevant interconnected pathways involving oxidative stress, inflammatory and immune responses, and gut microbiota [38, 39]. Findings from numerous randomized trials and prospective cohort studies have demonstrated a relationship between higher sodium intake and elevated blood pressure [1, 6], and the long-term pressure load from elevated systolic blood pressure can result in damage to multiple organs and tissues including blood vessels, and the heart, brain and kidneys. Sodium intake can affect endothelial function via oxidative stress and inflammation, and evidence from in vivo studies showed that high sodium intake may influence microenvironmental changes in endothelial cells and tissues, and increasing their stiffness through generation of pro-oxidant enzymes and oxygen free radicals, and decreasing nitric oxide (NO) production [38, 40]. Sodium concentrations may also affect the renin–angiotensin–aldosterone system (RAAS), a critical regulator of blood pressure to control body fluid homeostasis [41], and activation of the RAAS has been reported to be related to an increased risk of CVD morbidity and mortality [41]. High levels of sodium can also interfere with homeostasis of innate and adaptive immune systems [38, 42]. Findings from cell culture experiments demonstrate that increased sodium chloride concentrations can lead to alterations in immune profiles, including promoting activation of proinflammatory M1 macrophages and Th17 cells and inhibiting the differentiation of anti-inflammatory M2 macrophages and Treg cells [41]. Th17 cells have been demonstrated in atherosclerotic plaques and shown to exert proinflammatory effects by secreting substantial quantities of the proinflammatory cytokine interleukin 17A (IL-17A), and by promoting recruitment of pathogenic macrophages [41]; IL-17A has also been found to lead to endothelial dysfunction by activating RhoA/Rho-kinase and reducing NO bioavailability [43, 44]. In addition, previous studies have shown that sodium concentrations can affect gut microbiota, such as altering the composition of intestinal flora (i.e., the *Lactobacillus* population), and in turn exert a regulatory influence in inflammation, infection and cardiovascular disease [38, 39, 42].

We also observed that a higher sodium–potassium intake ratio was associated with an increased risk of overall and CVD mortality, which is consistent with findings from previous meta-analyses and cohort studies [11–13, 36]. Potassium is an important nutrient and the most abundant intracellular cation that plays critical roles in supporting cell functions including homeostasis [45], and has been proposed as having antihypertensive effects through different mechanisms. A high potassium diet associated with increased plasma potassium could lead to endothelial-dependent vasodilation by hyperpolarizing endothelial cells through sodium pump stimulation and opening of potassium channels. Additionally, potassium may enhance sodium excretion, decrease sensitivity to catecholamine-related vasoconstriction, and inhibit inflammation and oxidative stress [45, 46]. It has been reported that the sodium–potassium ratio derived from dietary composition may have a role in the development and progression of salt-induced hypertension [46]. Evidence from animal studies shows that increased dietary potassium intake can neutralize the adverse influence of a high sodium diet, lower blood pressure and extend the life span of rats fed a high sodium diet [46]. Yet, detailed biological mechanisms of the favorable effects of a high potassium diet are still not fully understood and require additional investigation and elucidation.

### Strengths and limitations of the study

This study has several strengths. The large sample size, number of events and long-term follow-up provided sufficient power to disentangle moderate associations between dietary sodium and potassium intake and risk of mortality, permitted assessment of associations at the lower and upper ends of the sodium distribution, and enabled our evaluation of sex-specific and cause-specific associations as well as effect modification by risk factors. Some limitations in the present study also deserve mention. First, although 24-h urinary sodium excretion is considered the most reliable estimation of sodium intake, in the present NIH-AARP Study analysis, intake was assessed through a food frequency questionnaire which entails inherent random measurement error that may result in underreported dietary sodium intake [47], and could lead to underestimation of the observed mortality associations (due to nondifferential exposure misclassification) and influence risk estimates toward the null. Supporting this, our updated meta-analysis showed that the positive sodium-CVD association was significantly stronger in studies which included repeated sodium measurements (pooled RR = 1.36) as compared with those with a single baseline measurement (pooled RR = 1.08). It is of note, however, that the updated meta-analysis provides data showing the consistency of findings from studies with sodium intake calculated from dietary questionnaires, spot urinary measurements or timed urinary measurements (pooled RRs = 1.11, 1.09 and 1.17 respectively); however, the potential influence of the different assessment approaches may remain and should not be discounted. Also, owing to the sparse publication data regarding sodium-cause-specific mortality associations, we have only focused on the CVD-related outcomes in the present meta-analysis. Further meta-analyses on the associations between sodium intake and other outcomes should be evaluated with additional accumulating data in the future. Second, the sodium and potassium intake data were not updated over time, and the baseline data may not be able to capture potential intake or composition changes during the follow-up period. We did conduct a sensitivity analysis stratified by follow-up time, and the sodium- and potassium-mortality risk estimates were stable across the follow-up periods of 0 to < 5 years, 5 to < 10 years, and 10 + , which implies temporal consistency of the associations. Third, given the nature of observational design, we cannot completely preclude the possibility of residual confounding from measured or unmeasured factors, which may have influenced the associations examined. We were, however, able to account and adjust for a wide range of important risk characteristics, and our findings were essentially unchanged. Fourth, using the median or midpoint value for each category as the proxy measure in the dose–response meta-analysis may inadequately reflect the true value of dietary sodium intake, thus potentially leading to bias in the dose relationship. Although NIH-AARP Diet and Health Study includes 416,104 US men and women from six states and two metropolitan areas, it is important to note that the NIH-AARP Study may not necessarily be representative of the general US population given the target population and voluntary participation.

## Conclusion

Our data show significant positive associations of higher sodium intake (within the range of sodium intake between 2,000 and 7,500 mg/d), a higher sodium–potassium ratio, or lower potassium intake, with risk of CVD and overall mortality, and women showed stronger mortality associations for potassium and the sodium–potassium ratio. Findings from the systematic review and updated meta-analysis provide additional compelling support for the association between higher sodium intake and increased risk of CVD. Our data may support decreasing sodium intake, and increasing potassium intake, as means to improve health and longevity. Given the current recommendations, our data pointing to a sex difference in the potassium-mortality and sodium–potassium-ratio-mortality relationships provide additional evidence relevant to dietary guidelines.

### Supplementary Information

**Additional file 1: Table S1.** The Underlying Causes of Death Based on the Ninth and Tenth Revisions of International Classification of Diseases (ICD-9, and ICD-10). **Table S2.** Criteria for Healthy Eating Index-2015 Score. **Table S3.** Risk of Overall and Cause-Specific Mortality Associated with Daily Intakes of Sodium and Potassium and the Sodium-Potassium Ratio Among 237,036 Men. **Table S4.** Risk of Overall and Cause-Specific Mortality Associated with Intakes of Sodium, Potassium and Sodium-Potassium Ratio Among 179,068 Women. **Table S5.** Risk of Overall and Cause-Specific Mortality Associated with per 500 mg or per 1-SD Sodium Intake Among 237,036 Men and 179,068 Women. **Table S6.** Risk of Overall and Cause-Specific Mortality Associated with per 500 mg or per 1-SD Potassium Intake Among 237,036 Men and 179,068 Women. **Table S7.** Risk of Overall and Cause-Specific Mortality Associated with per 1-SD Sodium-Potassium Ratio Among 237,036 Men and 179,068 Women. **Table S8.** Search Strategy for Systematic Review. **Table S9.** Studies included in the meta-analysis. **Table S10.** Study Characteristics in the Meta-Analysis. **Table S11.** Assessment of Risk of Study Bias Based on the Newcastle-Ottawa Scale. **Table S12.** Prespecified Subgroup Meta-Analysis Using Random-Effects Models for the Association Between Sodium Intake and Risk of Cardiovascular Disease, with the Comparison of Highest Versus Lowest Categories of Sodium Intake. **Fig. S1.** Sex Stratified Associations Between Sodium Intake and Overall and Cause-specific Mortality in Minimally Adjusted Cubic Spline Regression Models. **Fig. S2.** Sex Stratified Associations Between Potassium Intake and Overall and Cause-specific Mortality in Minimally Adjusted Cubic Spline Regression Models. **Fig. S3.** Sex Stratified Associations Between Intake of Sodium-Potassium Ratio and Overall and Cause-specific Mortality in Minimally Adjusted Cubic Spline Regression Models. **Fig. S4.** Sex Stratified Associations Between Sodium Intake and Overall and Cause-specific Mortality in Parsimoniously Adjusted Cubic Spline Regression Models. **Fig. S5.** Sex Stratified Associations Between Potassium Intake and Overall and Cause-specific Mortality in Parsimoniously Adjusted Cubic Spline Regression Models. **Fig. S6.** Sex Stratified Associations Between Intake of Sodium-Potassium Ratio and Overall and Cause-specific Mortality in Parsimoniously Adjusted Cubic Spline Regression Models. **Fig. S7.** Sex Stratified Associations Between Sodium Intake and Overall and Cause-specific Mortality in Multivariable-Adjusted Cubic Spline Regression Models, Excluding the Initial 5 Years of Follow-up. **Fig. S8.** Sex Stratified Associations Between Potassium Intake and Overall and Cause-specific Mortality in Multivariable-Adjusted Cubic Spline Regression Models, Excluding the Initial 5 Years of Follow-up. **Fig. S9.** Sex Stratified Associations Between Sodium-Potassium Ratio and Overall and Cause-specific Mortality in Multivariable-Adjusted Cubic Spline Regression Models, Excluding the Initial 5 Years of Follow-up. **Fig. S10.** Sex Stratified Associations Between Sodium Intake and Overall and Cause-specific Mortality in Multivariable-Adjusted Cubic Spline Regression Models, Excluding the Participants Reporting a History of Diabetes at Baseline. **Fig. S11.** Sex Stratified Associations Between Potassium Intake and Overall and Cause-specific Mortality in Multivariable-Adjusted Cubic Spline Regression Models, Excluding the Participants Reporting a History of Diabetes at Baseline. **Fig. S12.** Sex Stratified Associations Between Sodium-Potassium Ratio and Overall and Cause-specific Mortality in Multivariable-Adjusted Cubic Spline Regression Models, Excluding the Participants Reporting a History of Diabetes at Baseline. **Fig. S13.** Sex-Stratified Associations Between Sodium Intake and Overall and Cause-specific Mortality in Multivariable-Adjusted Cubic Spline Regression Models, Excluding Participants Reporting Poor to Fair Health Status or Unknown Health Status. **Fig. S14.** Sex-Stratified Associations Between Potassium Intake and Overall and Cause-specific Mortality in Multivariable-Adjusted Cubic Spline Regression Models, Excluding Participants Reporting Poor to Fair Health Status or Unknown Health Status. **Fig. S15.** Sex-Stratified Associations Between Sodium-Potassium Ratio and Overall and Cause-specific Mortality in Multivariable-Adjusted Cubic Spline Regression Models, Excluding Participants Reporting Poor to Fair Health Status or Unknown Health Status. **Fig. S16.** Sex-Stratified Associations Between Sodium Intake and Overall and Cause-specific Mortality in Multivariable-Adjusted Cubic Spline Regression Models, Additionally Adjusted for Family Income. **Fig. S17.** Sex Stratified Associations Between Potassium Intake and Overall and Cause-specific Mortality in Multivariable-Adjusted Cubic Spline Regression Models, Additionally Adjusted for Family Income. **Fig. S18.** Sex Stratified Associations Between Sodium-Potassium Ratio and Overall and Cause-specific Mortality in Multivariable-Adjusted Cubic Spline Regression Models, Additionally Adjusted for Family Income. **Fig. S19.** Sex-Stratified Associations Between Sodium Intake and Overall and Cause-specific Mortality in Multivariable-Adjusted Cubic Spline Regression Models, Additionally Adjusted for Dietary Potassium Intake. **Fig. S20.** Flow Chat for Study Selection Strategy in the Meta-Analysis. **Fig. S21.** Funnel Plot for Assessment of Publication Bias for the Association Between Sodium Intake and Risk of Cardiovascular Disease (Including Death Due to Cardiovascular Disease). **Fig. S22.** Influence Analysis Using Forest Plot for the Meta-Analysis on Associations Between Sodium Intake and Risk of CVD (Including CVD Mortality).

## Data Availability

The NIH-AARP Diet and Health Study was developed by the Nutritional Epidemiology Branch of the National Cancer Institute (NCI), a component of the National Institutes of Health, to improve our understanding of the relationship between diet and health. (Website: https://dietandhealth.cancer.gov/resource/) Data requests are subject to approval by the study review committees.

## Abbreviations

ARD: Absolute risk difference
BMI: Body mass index
CI: Confidence interval
CVD: Cardiovascular disease
DHQ: Diet History Questionnaire
HEI-2015: Healthy Eating Index 2015
HR: Hazard ratio
ICD: International Classification of Diseases
IL-17A: Interleukin 17A
IQR: Interquartile range
NIH-AARP: National Institutes of Health-American Association of Retired Persons
PRISMA: Preferred Reporting Items for Systematic reviews and Meta-Analysis
RAAS: Renin–angiotensin–aldosterone system
RR: Relative Risk
SD: Standard deviation
