# Supplementary material for: Sex-specific associations between sodium and potassium intake and overall and cause-specific mortality: a large prospective U.S. cohort study, systematic review, and updated meta-analysis of cohort studies
Source: BMC Med. 2024 Mar 22;22:132. doi: 10.1186/s12916-024-03350-x (PMC10960470; doi:10.1186/s12916-024-03350-x)
Supplement: Supplementary file 1 — Additional file 1: Table S1. The Underlying Causes of Death Based on the Ninth and Tenth Revisions of International Classification of Diseases (ICD-9, and ICD-10). Table S2. Criteria for Healthy Eating Index-2015 Score. Table S3. Risk of Overall and Cause-Specific Mortality Associated with Daily Intakes of Sodium and Potassium and the Sodium-Potassium Ratio Among 237,036 Men. Table S4. Risk of Overall and Cause-Specific Mortality Associated with Intakes of Sodium, Potassium and Sodium-Potassium Ratio Among 179,068 Women. Table S5. Risk of Overall and Cause-Specific Mortality Associated with per 500 mg or per 1-SD Sodium Intake Among 237,036 Men and 179,068 Women. Table S6. Risk of Overall and Cause-Specific Mortality Associated with per 500 mg or per 1-SD Potassium Intake Among 237,036 Men and 179,068 Women. Table S7. Risk of Overall and Cause-Specific Mortality Associated with per 1-SD Sodium-Potassium Ratio Among 237,036 Men and 179,068 Women. Table S8. Search Strategy for Systematic Review. Table S9. Studies included in the meta-analysis. Table S10. Study Characteristics in the Meta-Analysis. Table S11. Assessment of Risk of Study Bias Based on the Newcastle-Ottawa Scale. Table S12. Prespecified Subgroup Meta-Analysis Using Random-Effects Models for the Association Between Sodium Intake and Risk of Cardiovascular Disease, with the Comparison of Highest Versus Lowest Categories of Sodium Intake. Fig. S1. Sex Stratified Associations Between Sodium Intake and Overall and Cause-specific Mortality in Minimally Adjusted Cubic Spline Regression Models. Fig. S2. Sex Stratified Associations Between Potassium Intake and Overall and Cause-specific Mortality in Minimally Adjusted Cubic Spline Regression Models. Fig. S3. Sex Stratified Associations Between Intake of Sodium-Potassium Ratio and Overall and Cause-specific Mortality in Minimally Adjusted Cubic Spline Regression Models. Fig. S4. Sex Stratified Associations Between Sodium Intake and Overall and Cause-specific Morta [file 12916_2024_3350_MOESM1_ESM.docx]

**Table S1**. The Underlying Causes of Death Based on the Ninth and Tenth Revisions of International Classification of Diseases (ICD-9, and ICD-10).

| **Underlying causes of death** | **ICD-9** | **ICD-10** |
| --- | --- | --- |
| **CVD** | 390-459 | I00-I99 |
| **Heart disease** | 390-398, 401-404, 410-429 and 440-448 | I00-I13, I20-I51, and I70-I78 |
| **Stroke** | 430-438 | I60-I69 |
| **Cancer** | 140-239 | C00-C97 and D00-D48 |
| **Respiratory disease** | 480-487 and 490-496 | J10-J18 and J40-J47 |
| **Infectious disease** | 001-139 | A00-B99 |
| **Injuries and accidents** | 800-978 | V01-X59, Y85-Y86, U03, X60-X84, Y87.0, U01-U02, X85-Y09, Y35, Y87.1, and Y89.0 |

**Table S2**. Criteria for Healthy Eating Index-2015 Score

| **Dietary component** | **Maximum score** | **Standard for maximum score** | **Minimum score** | **Standard for minimum score** |
| --- | --- | --- | --- | --- |
| Total Fruits | 5 | ≥0.8 cup equivalents/1,000 kcal | 0 | No fruit |
| Whole Fruits | 5 | ≥0.4 cup equivalents/1,000 kcal | 0 | No whole fruit |
| Total Vegetables | 5 | ≥1.1 cup equivalents/1,000 kcal | 0 | No vegetables |
| Greens and Beans | 5 | ≥0.2 cup equivalents/1,000 kcal | 0 | No dark green vegetables or beans and peas |
| Whole grains | 10 | ≥1.5 oz equivalents/1,000 kcal | 0 | No whole grains |
| Dairy | 10 | ≥1.3 cup equivalents/1,000 kcal | 0 | No dairy |
| Total protein foods | 5 | ≥2.5 oz equivalents/1,000 kcal | 0 | No protein foods |
| Seafood and plant proteins | 5 | ≥0.8 cup equivalents/1,000 kcal | 0 | No seafood or plant proteins |
| Fatty acids | 10 | (Polyunsaturated fatty acids + Monounsaturated fatty acids)/Saturated fatty acids ≥2.5 | 0 | (Polyunsaturated fatty acids + Monounsaturated fatty acids)/Saturated fatty acids ≤1.2 |
| Refined grains | 10 | ≤1.8 oz equivalents/1,000 kcal | 0 | ≥4.3 oz equivalents/1,000 kcal |
| Sodium | 10 | ≤1.1 g/1,000 kcal | 0 | ≥2.0 g/1,000 kcal |
| Added sugars | 10 | ≤6.5% of energy | 0 | ≥26% of energy |
| Saturated fats | 10 | ≤8% of energy | 0 | ≥16% of energy |

**Table S3.** Risk of Overall and Cause-Specific Mortality Associated with Daily Intakes of Sodium and Potassium and the Sodium-Potassium Ratio Among 237,036 Men ^a^

| **Sex, Intake and Causes** | **Category 1** | **Category 2** | **Category 3** | **Category 4** | **Category 5** | **ARD, %**  **(95% CI) ^b^** | **P for trend (linear) ^c^** | **P value (Non-linear) ^c^** |
| --- | --- | --- | --- | --- | --- | --- | --- | --- |
|  | **HR (95% CI)** | **HR (95% CI)** | **HR (95% CI)** | **HR (95% CI)** | **HR (95% CI)** |  |  |  |
| **Sodium intake, mg (range)** | <2,000  (n=48,830) | 2,000 to <3,000  (n=84,825) | 3,000 to <4,000  (n=59,371) | 4,000 to <5,000  (n=26,232) | >5,000  (n=17,778) |  |  |  |
| Overall mortality | 1.04 (1.01, 1.06) | 1.00 | 1.00 (0.98, 1.03) | 1.05 (1.01, 1.09) | 1.12 (1.06, 1.18) | 0.84 (0.40, 1.37) | 0.02^*^ | <0.0001^***^ |
| CVD | 1.06 (1.00, 1.11) | 1.00 | 1.00 (0.95, 1.05) | 1.07 (0.99, 1.14) | 1.11 (1.00, 1.22) | 0.41 (0.06, 0.81) | 0.32 | 0.0002^***^ |
| Heart disease | 1.04 (0.98, 1.10) | 1.00 | 1.00 (0.95, 1.06) | 1.07 (0.99, 1.15) | 1.15 (1.03, 1.28) | 0.41 (0.07, 0.78) | 0.11 | 0.0002^***^ |
| Stroke | 1.13 (1.00, 1.29) | 1.00 | 0.98 (0.86, 1.11) | 1.02 (0.85, 1.22) | 0.82 (0.62, 1.08) | -0.04 (-0.19, 0.13) | 0.15 | 0.46 |
| Cancer | 1.00 (0.95, 1.05) | 1.00 | 0.99 (0.95, 1.04) | 1.06 (0.99, 1.13) | 1.14 (1.04, 1.25) | 0.49 (0.12, 0.91) | 0.04^*^ | 0.31 |
| Respiratory disease | 0.87 (0.78, 0.98) | 1.00 | 1.04 (0.94, 1.15) | 1.15 (0.99, 1.33) | 1.43 (1.16, 1.76) | 0.43 (0.24, 0.65) | 0.0005^***^ | 0.65 |
| Infectious disease | 1.10 (0.96, 1.27) | 1.00 | 0.98 (0.86, 1.12) | 0.82 (0.67, 1.01) | 0.85 (0.63, 1.13) | -0.15 (-0.29, 0.02) | 0.07 | 0.37 |
| Injury and accidents | 1.16 (1.01, 1.33) | 1.00 | 1.02 (0.90, 1.17) | 0.86 (0.71, 1.05) | 1.06 (0.81, 1.40) | 0 (-0.12, 0.13) | 0.35 | 0.01^*^ |
| Other causes | 1.09 (1.03, 1.16) | 1.00 | 1.02 (0.96, 1.08) | 1.06 (0.97, 1.25) | 1.10 (0.97, 1.25) | 0.02 (-0.26, 0.30) | 0.70 | 0.0002^***^ |
| **Potassium intake, mg (range)** | <2,100  (n=26,349) | 2,100 to <2,528  (n=26,389) | 2,528 to <2,874  (n=26,318) | 2,874 to <3,200  (n=26,361) | >3,200  (n=131,619) |  |  |  |
| Overall mortality | 1.00 | 0.90 (0.87, 0.94) | 0.87 (0.84, 0.91) | 0.86 (0.83, 0.89) | 0.84 (0.81, 0.87) | -0.39 (-0.65, -0.14) | <0.0001^***^ | <0.0001^***^ |
| CVD | 1.00 | 0.87 (0.81, 0.93) | 0.87 (0.81, 0.93) | 0.84 (0.79, 0.91) | 0.83 (0.77, 0.88) | -0.03 (-0.20, 0.14) | <0.0001^***^ | <0.0001^***^ |
| Heart disease | 1.00 | 0.88 (0.82, 0.95) | 0.88 (0.82, 0.95) | 0.86 (0.79, 0.93) | 0.84 (0.78, 0.90) | 0 (-0.16, 0.15) | <0.0001^***^ | <0.0001^***^ |
| Stroke | 1.00 | 0.83 (0.70, 0.99) | 0.88 (0.73, 1.04) | 0.79 (0.66, 0.95) | 0.77 (0.65, 0.91) | -0.05 (-0.13, 0.03) | 0.009^**^ | 0.002^**^ |
| Cancer | 1.00 | 0.96 (0.90, 1.02) | 0.92 (0.87, 0.99) | 0.91 (0.86, 0.97) | 0.92 (0.86, 0.97) | -0.20 (-0.41, -0.02) | 0.01^*^ | 0.06 |
| Respiratory disease | 1.00 | 0.92 (0.80, 1.06) | 0.92 (0.79, 1.07) | 0.84 (0.72, 0.98) | 0.85 (0.74, 0.98) | -0.07 (-0.18, 0.05) | 0.04^*^ | 0.09 |
| Infectious disease | 1.00 | 0.94 (0.78, 1.15) | 1.06 (0.87, 1.28) | 0.92 (0.75, 1.13) | 0.88 (0.73, 1.06) | 0 (-0.07, 0.08) | 0.09 | 0.26 |
| Injury and accidents | 1.00 | 0.90 (0.74, 1.09) | 0.76 (0.62, 0.93) | 0.84 (0.69, 1.02) | 0.76 (0.63, 0.91) | -0.02 (-0.10, 0.05) | 0.006^**^ | 0.005^**^ |
| Other causes | 1.00 | 0.84 (0.78, 0.91) | 0.77 (0.71, 0.84) | 0.80 (0.73, 0.87) | 0.76 (0.70, 0.82) | -0.22 (-0.38, -0.09) | <0.0001^***^ | <0.0001^***^ |
| **Sodium-potassium ratio (range)** | <0.8  (n=97,934) | 0.8 to <0.88  (n=35,411) | 0.88 to <0.97  (n=35,265) | 0.97 to <1.09  (n=33,787) | >1.09  (n=34,639) |  |  |  |
| Overall mortality | 1.00 | 1.01 (0.98, 1.04) | 1.01 (0.99, 1.04) | 1.02 (0.99, 1.06) | 1.13 (1.09, 1.16) | 0.61 (0.42, 0.83) | <0.0001^***^ | <0.0001^***^ |
| CVD | 1.00 | 1.02 (0.97, 1.08) | 0.98 (0.93, 1.04) | 1.02 (0.96, 1.07) | 1.15 (1.09, 1.22) | 0.27 (0.15, 0.42) | <0.0001^***^ | 0.0003^***^ |
| Heart disease | 1.00 | 1.02 (0.96, 1.08) | 0.98 (0.93, 1.04) | 1.01 (0.95, 1.07) | 1.14 (1.07, 1.21) | 0.21 (0.09, 0.33) | 0.001^**^ | 0.002^**^ |
| Stroke | 1.00 | 1.05 (0.92, 1.19) | 1.02 (0.89, 1.16) | 1.01 (0.87, 1.17) | 1.25 (1.08,1.45) | 0.09 (0.02, 0.16) | 0.02^*^ | 0.13 |
| Cancer | 1.00 | 1.00 (0.96, 1.05) | 1.01 (0.96, 1.06) | 1.03 (0.98, 1.09) | 1.05 (1.00, 1.11) | 0.11 (-0.03, 0.26) | 0.04^*^ | 0.94 |
| Respiratory disease | 1.00 | 1.03 (0.92, 1.15) | 1.10 (0.98, 1.23) | 1.11 (0.99, 1.24) | 1.34 (1.19, 1.50) | 0.17 (0.10, 0.24) | <0.0001^***^ | 0.09 |
| Infectious disease | 1.00 | 0.95 (0.82, 1.09) | 0.92 (0.79, 1.07) | 0.84 (0.72, 0.99) | 0.95 (0.81, 1.12) | -0.02 (-0.08, 0.04) | 0.22 | 0.07 |
| Injury and accidents | 1.00 | 0.94 (0.82, 1.09) | 1.02 (0.88, 1.18) | 1.01 (0.86, 1.17) | 1.14 (0.97, 1.34) | 0.04 (-0.01, 0.11) | 0.17 | 0.12 |
| Other causes | 1.00 | 1.01 (0.95, 1.08) | 1.06 (1.00, 1.13) | 1.04 (0.97, 1.11) | 1.20 (1.12, 1.28) | 0.26 (0.17, 0.39) | <0.0001^***^ | 0.06 |

^a^ Hazard ratios (HRs) and their 95% confidence intervals (CIs) for mortality comparing the highest category with the referent (referent for sodium: the second lowest category, and referent for potassium and sodium-potassium: the lowest category). All categories, including the second category as the referent group for sodium, were set based on the data from cubic-restricted splines. Multivariable analyses were adjusted for age at baseline, BMI, alcohol consumption, smoking status (never, former, current or missing), physical activity, race or ethnic group, education, marital status, diabetes (yes vs. no), health status, vitamin supplement use, and total energy intake. For sodium intake, models were additionally adjusted for Healthy Eating Index 2015 (HEI-2015) score excluding the sodium component; for potassium intake and the sodium-potassium ratio, models were additionally adjusted for HEI-2015 components for sodium (potassium model only), seafood and plant protein, saturated fat, fatty acids and refined grains. P for trend was calculated based on statistical significance of the coefficient of the category variable (median value assigned for each category).

^b^ Adjusted absolute risk differences (ARD) were estimated based on hazard ratios of overall and cause-specific mortality for a difference between highest categories and referent categories of sodium, potassium and sodium-potassium ratio intakes during the follow-up of 16 years. The 95% CIs were computed based on 100 bootstrap samples.

^c^ ^*^p < 0.05, ^**^p < 0.01, ^***^p < 0.00093 (the Bonferroni corrected threshold).

**Table S4.** Risk of Overall and Cause-Specific Mortality Associated with Intakes of Sodium, Potassium and Sodium-Potassium Ratio Among 179,068 Women ^a^

| **Sex, Intake and Cause** | **Category 1** | **Category 2** | **Category 3** | **Category 4** | **Category 5** | **ARD, %**  **(95% CI) ^b^** | **P for trend (linear) ^c^** | **P value (Non-linear) ^c^** |
| --- | --- | --- | --- | --- | --- | --- | --- | --- |
|  | **HR (95% CI)** | **HR (95% CI)** | **HR (95% CI)** | **HR (95% CI)** | **HR (95% CI)** |  |  |  |
| **Sodium intake, mg (range)** | <1,400  (n=27,026) | 1,400 to <2,200  (n=63,146) | 2,200 to <3,000  (n=49,747) | 3,000 to <4,000  (n=26,404) | >4,000  (n=12,745) |  |  |  |
| Overall mortality | 1.07 (1.03, 1.11) | 1.00 | 1.02 (0.98, 1.05) | 1.09 (1.04, 1.14) | 1.14 (1.06, 1.23) | 0.73 (0.20, 1.24) | 0.02^*^ | <0.0001^***^ |
| CVD | 1.08 (1.00, 1.16) | 1.00 | 1.05 (0.98, 1.12) | 1.14 (1.04, 1.25) | 1.29 (1.11, 1.49) | 0.56 (0.19, 0.96) | 0.01^*^ | 0.01^*^ |
| Heart disease | 1.05 (0.96, 1.14) | 1.00 | 1.03 (0.96, 1.11) | 1.18 (1.06, 1.32) | 1.35 (1.14, 1.59) | 0.58 (0.25, 0.95) | 0.03^*^ | 0.03^*^ |
| Stroke | 1.18 (1.01, 1.39) | 1.00 | 1.04 (0.90, 1.20) | 1.00 (0.81, 1.23) | 1.10 (0.79, 1.51) | 0.01 (-0.13, 0.16) | 0.81 | 0.25 |
| Cancer | 1.06 (1.00, 1.13) | 1.00 | 1.02 (0.96, 1.08) | 1.08 (1.00, 1.18) | 1.10 (0.96, 1.25) | 0.09 (-0.30, 0.48) | 0.30 | 0.12 |
| Respiratory disease | 0.95 (0.83, 1.09) | 1.00 | 1.05 (0.94, 1.18) | 1.14 (0.97, 1.34) | 1.19 (0.92, 1.53) | 0.32 (0.13, 0.53) | 0.10 | 0.39 |
| Infectious disease | 1.23 (1.02, 1.49) | 1.00 | 1.00 (0.84, 1.19) | 1.06 (0.84, 1.35) | 0.92 (0.63, 1.35) | -0.03 (-0.17, 0.10) | 0.47 | 0.14 |
| Injury and accidents | 0.91 (0.71, 1.17) | 1.00 | 1.13 (0.92, 1.38) | 1.02 (0.75, 1.39) | 1.46 (0.92, 2.32) | 0 (-0.11, 0.11) | 0.18 | 0.30 |
| Other causes | 1.11 (1.02, 1.20) | 1.00 | 0.96 (0.89, 1.03) | 1.02 (0.92, 1.13) | 1.03 (0.88, 1.21) | 0.05 (-0.28, 0.34) | 0.68 | 0.001^**^ |
| **Potassium intake, mg (range)** | <2,087  (n=35,813) | 2,087 to <2,645  (n=35,815) | 2,645 to <3,210  (n=35,813) | 3,210 to <4,000  (n=35,814) | >4,000  (n=35,813) |  |  |  |
| Overall mortality | 1.00 | 0.91 (0.88, 0.94) | 0.84 (0.81, 0.88) | 0.83 (0.80, 0.87) | 0.81 (0.76, 0.86) | -0.49 (-0.75, -0.48) | <0.0001^***^ | <0.0001^***^ |
| CVD | 1.00 | 0.83 (0.77, 0.89) | 0.82 (0.75, 0.88) | 0.83 (0.76, 0.91) | 0.75 (0.67, 0.85) | -0.04 (-0.18, -0.03) | <0.0001^***^ | <0.0001^***^ |
| Heart disease | 1.00 | 0.84 (0.77, 0.92) | 0.82 (0.75, 0.90) | 0.81 (0.74, 0.90) | 0.77 (0.67, 0.87) | 0 (-0.15, 0.02) | 0.0003^***^ | 0.0006^***^ |
| Stroke | 1.00 | 0.83 (0.70, 0.97) | 0.82 (0.69, 0.97) | 0.90 (0.75, 1.09) | 0.76 (0.59, 0.98) | -0.06 (-0.17, 0.01) | 0.13 | 0.09 |
| Cancer | 1.00 | 0.95 (0.89, 1.01) | 0.91 (0.85, 0.98) | 0.90 (0.83, 0.97) | 0.90 (0.81, 0.99) | -0.26 (-0.51, -0.14) | 0.03^*^ | 0.10 |
| Respiratory disease | 1.00 | 0.96 (0.85, 1.09) | 0.83 (0.72, 0.95) | 0.73 (0.63, 0.85) | 0.68 (0.56, 0.84) | -0.09 (-0.20, 0.07) | <0.0001^***^ | 0.07 |
| Infectious disease | 1.00 | 0.97 (0.81, 1.17) | 0.76 (0.62, 0.94) | 0.81 (0.65, 1.01) | 0.71 (0.53, 0.96) | 0 (-0.11, 0.09) | 0.01^*^ | 0.35 |
| Injury and accidents | 1.00 | 0.98 (0.77, 1.24) | 0.82 (0.63, 1.06) | 0.78 (0.59, 1.04) | 0.73 (0.50, 1.05) | -0.02 (-0.07, -0.01) | 0.05 | 0.27 |
| Other causes | 1.00 | 0.92 (0.85, 1.00) | 0.78 (0.72, 0.86) | 0.80 (0.73, 0.88) | 0.83 (0.73, 0.94) | -0.27 (-0.56, -0.14) | 0.001^**^ | <0.0001^***^ |
| **Sodium-potassium ratio (range)** | <0.8  (n=102,986) | 0.8 to <0.88  (n=23,665) | 0.88 to <0.97  (n=20,195) | 0.97 to <1.09  (n=16,488) | >1.09  (n=15,734) |  |  |  |
| Overall mortality | 1.00 | 1.04 (1.00, 1.08) | 1.07 (1.02, 1.11) | 1.12 (1.07, 1.17) | 1.24 (1.19, 1.30) | 0.64 (0.44, 0.87) | <0.0001^***^ | 0.04^*^ |
| CVD | 1.00 | 1.10 (1.02, 1.18) | 1.12 (1.03, 1.21) | 1.18 (1.08, 1.28) | 1.42 (1.31, 1.55) | 0.29 (0.15, 0.45) | <0.0001^***^ | 0.12 |
| Heart disease | 1.00 | 1.07 (0.99, 1.17) | 1.14 (1.04, 1.24) | 1.21 (1.10, 1.33) | 1.46 (1.33, 1.61) | 0.22 (0.10, 0.35) | <0.0001^***^ | 0.16 |
| Stroke | 1.00 | 1.13 (0.96, 1.31) | 1.05 (0.88, 1.25) | 1.08 (0.89, 1.31) | 1.29 (1.06, 1.57) | 0.09 (0.02, 0.17) | 0.02^*^ | 0.19 |
| Cancer | 1.00 | 0.99 (0.93, 1.05) | 1.03 (0.96, 1.10) | 1.06 (0.98, 1.14) | 1.06 (0.98, 1.15) | 0.11 (-0.03, 0.28) | 0.09 | 0.44 |
| Respiratory disease | 1.00 | 1.04 (0.92, 1.19) | 1.11 (0.97, 1.27) | 1.23 (1.06, 1.41) | 1.35 (1.17, 1.56) | 0.18 (0.11, 0.26) | <0.0001^***^ | 0.32 |
| Infectious disease | 1.00 | 1.07 (0.88, 1.29) | 1.12 (0.92, 1.38) | 1.11 (0.89, 1.39) | 1.34 (1.07, 1.67) | -0.02 (-0.09, 0.04) | 0.02^*^ | 0.61 |
| Injury and accidents | 1.00 | 1.04 (0.82, 1.31) | 1.32 (1.03, 1.67) | 1.32 (1.01, 1.73) | 1.19 (0.88, 1.61) | 0.05 (-0.01, 0.12) | 0.05 | 0.61 |
| Other causes | 1.00 | 1.08 (1.00, 1.17) | 1.01 (0.93, 1.11) | 1.11 (1.01, 1.22) | 1.30 (1.18, 1.43) | 0.28 (0.17, 0.41) | <0.0001^***^ | 0.33 |

^a^ Hazard ratios (HRs) and their 95% confidence intervals (CIs) for mortality comparing the highest category with the referent (referent for sodium: the second lowest category, and referent for potassium and sodium-potassium: the lowest category). All categories, including the second category as the referent group for sodium, were set based on the data from cubic-restricted splines. Multivariable analyses were adjusted for age at baseline, BMI, alcohol consumption, smoking status (never, former, current or missing), physical activity, race or ethnic group, education, marital status, diabetes (yes vs. no), health status, vitamin supplement use, and total energy intake. For sodium intake, models were additionally adjusted for Healthy Eating Index 2015 (HEI-2015) score excluding the sodium component; for potassium intake and the sodium-potassium ratio, models were additionally adjusted for HEI-2015 components for sodium (potassium model only), seafood and plant protein, saturated fat, fatty acids and refined grains. For women, the risk estimates were additionally adjusted for postmenopausal hormone therapy (yes vs. no). P for trend was calculated based on statistical significance of the coefficient of the category variable (median value assigned for each category).

^b^ Adjusted absolute risk differences (ARD) were estimated based on hazard ratios of overall and cause-specific mortality for a difference between highest categories and referent categories of sodium, potassium and sodium-potassium ratio intakes during the follow-up of 16 years. The 95% CIs were computed based on 100 bootstrap samples.

^c^ ^*^p < 0.05, ^**^p < 0.01, ^***^p < 0.00093 (the Bonferroni corrected threshold).

**Table S5.** Risk of Overall and Cause-Specific Mortality Associated with per 500 mg or per 1-SD Sodium Intake Among 237,036 Men and 179,068 Women ^a^

| **Sex, Intake and Causes** | **Per 500 mg** | **P value** | **Per 1-s.d.** | **P value**  **(Linear)** | **P value**  **(Non-linear)** |
| --- | --- | --- | --- | --- | --- |
|  | **HR (95% CI)** |  | **HR (95% CI)** |  |  |
| **Men** |  |  |  |  |  |
| Overall mortality | 1.02 (1.01, 1.023) | 0.0003 | 1.04 (1.02, 1.06) | 0.0003 | <0.0001 |
| CVD | 1.02 (1.01, 1.03) | 0.0099 | 1.05 (1.01, 1.09) | 0.0099 | 0.0002 |
| Heart disease | 1.02 (1.01, 1.04) | 0.0071 | 1.06 (1.02, 1.10) | 0.0071 | 0.0002 |
| Stroke | 0.99 (0.95, 1.03) | 0.59 | 0.97 (0.88, 1.08) | 0.59 | 0.46 |
| Cancer | 1.02 (1.01, 1.03) | 0.0066 | 1.05 (1.01, 1.09) | 0.0066 | 0.31 |
| Respiratory disease | 1.07 (1.04, 1.10) | <0.0001 | 1.20 (1.11, 1.29) | <0.0001 | 0.65 |
| Infectious disease | 0.95 (0.92, 0.99) | 0.025 | 0.88 (0.79, 0.99) | 0.025 | 0.37 |
| Injury and accidents | 1.00 (0.96, 1.04) | 1.00 | 1.00 (0.90, 1.11) | 1.00 | 0.01 |
| Other causes | 1.00 (0.98, 1.02) | 0.90 | 1.00 (0.96, 1.05) | 0.90 | 0.0002 |
| **Women** |  |  |  |  |  |
| Overall mortality | 1.02 (1.01, 1.04) | 0.0008 | 1.05 (1.02, 1.08) | 0.0008 | <0.0001 |
| CVD | 1.05 (1.02, 1.07) | 0.0002 | 1.10 (1.05, 1.16) | 0.0002 | 0.01 |
| Heart disease | 1.06 (1.03, 1.09) | <0.0001 | 1.13 (1.07, 1.20) | <0.0001 | 0.03 |
| Stroke | 1.00 (0.95, 1.06) | 0.87 | 1.01 (0.90, 1.13) | 0.87 | 0.25 |
| Cancer | 1.01 (0.98, 1.03) | 0.60 | 1.01 (0.97, 1.06) | 0.60 | 0.12 |
| Respiratory disease | 1.07 (1.03, 1.12) | 0.0008 | 1.16 (1.06, 1.26) | 0.0008 | 0.39 |
| Infectious disease | 0.98 (0.92, 1.05) | 0.62 | 0.97 (0.85, 1.11) | 0.62 | 0.14 |
| Injury and accidents | 1.00 (0.92, 1.08) | 0.94 | 0.99 (0.84, 1.18) | 0.94 | 0.30 |
| Other causes | 1.00 (0.98, 1.03) | 0.71 | 1.01 (0.96, 1.07) | 0.71 | 0.001 |

^a^ Multivariable analyses were adjusted for age at baseline, BMI, alcohol consumption, smoking status (never, former, current or missing), physical activity, race or ethnic group, education, marital status, diabetes (yes vs. no), health status, vitamin supplement use, and total energy intake. For sodium intake, models were additionally adjusted for Healthy Eating Index 2015 (HEI-2015) score excluding the sodium component; for potassium intake and the sodium-potassium ratio, models were additionally adjusted for HEI-2015 components for sodium (potassium model only), seafood and plant protein, saturated fat, fatty acids and refined grains. P for trend was calculated based on statistical significance of the coefficient of the category variable (median value assigned for each category).

**Table S6.** Risk of Overall and Cause-Specific Mortality Associated with per 500 mg or per 1-SD Potassium Intake Among 237,036 Men and 179,068 Women ^a^

| **Sex, Intake and Causes** | **Per 500 mg** | **P value**  **(Linear)** | **Per 1-s.d.** | **P value**  **(Linear)** | **P value**  **(Non-linear)** |
| --- | --- | --- | --- | --- | --- |
|  | **HR (95% CI)** |  | **HR (95% CI)** |  |  |
| **Men** |  |  |  |  |  |
| Overall mortality | 0.99 (0.98, 0.995) | 0.0008 | 0.97 (0.95, 0.99) | 0.0008 | <0.0001 |
| CVD | 1.00 (0.99, 1.01) | 0.70 | 0.99 (0.96, 1.03) | 0.70 | <0.0001 |
| Heart disease | 1.00 (0.99, 1.01) | 0.92 | 1.00 (0.96, 1.04) | 0.92 | <0.0001 |
| Stroke | 0.98 (0.95, 1.01) | 0.24 | 0.95 (0.87, 1.04) | 0.24 | 0.002 |
| Cancer | 0.99 (0.98, 1.00) | 0.026 | 0.97 (0.94, 1.00) | 0.026 | 0.06 |
| Respiratory disease | 0.98 (0.96, 1.01) | 0.12 | 0.95 (0.88, 1.02) | 0.12 | 0.09 |
| Infectious disease | 1.00 (0.97, 1.03) | 0.97 | 1.00 (0.91, 1.10) | 0.97 | 0.26 |
| Injury and accidents | 0.99 (0.96, 1.02) | 0.59 | 0.98 (0.89, 1.07) | 0.59 | 0.005 |
| Other causes | 0.98 (0.96, 0.99) | 0.0018 | 0.94 (0.90, 0.98) | 0.0018 | <0.0001 |
| **Women** |  |  |  |  |  |
| Overall mortality | 0.97 (0.96, 0.98) | <0.0001 | 0.93 (0.91, 0.96) | <0.0001 | <0.0001 |
| CVD | 0.97 (0.95, 0.98) | 0.0002 | 0.92 (0.88, 0.96) | 0.0002 | <0.0001 |
| Heart disease | 0.97 (0.95, 0.99) | 0.0012 | 0.92 (0.87, 0.97) | 0.0012 | 0.0006 |
| Stroke | 0.97 (0.93, 1.01) | 0.14 | 0.93 (0.84, 1.03) | 0.14 | 0.09 |
| Cancer | 0.99 (0.98, 1.01) | 0.36 | 0.98 (0.94, 1.02) | 0.36 | 0.10 |
| Respiratory disease | 0.93 (0.90, 0.95) | <0.0001 | 0.82 (0.75, 0.88) | <0.0001 | 0.07 |
| Infectious disease | 0.94 (0.90, 0.98) | 0.0054 | 0.85 (0.75, 0.95) | 0.0054 | 0.35 |
| Injury and accidents | 0.97 (0.91, 1.02) | 0.25 | 0.92 (0.79, 1.06) | 0.25 | 0.27 |
| Other causes | 0.98 (0.96, 1.00) | 0.020 | 0.94 (0.89, 0.99) | 0.020 | <0.0001 |

^a^ Hazard ratios (HRs) and their 95% confidence intervals (CIs) for mortality comparing the highest category with the referent (referent for sodium: the second lowest category, and referent for potassium and sodium-potassium: the lowest category). Multivariable analyses were adjusted for age at baseline, BMI, alcohol consumption, smoking status (never, former, current or missing), physical activity, race or ethnic group, education, marital status, diabetes (yes vs. no), health status, vitamin supplement use, and total energy intake. For sodium intake, models were additionally adjusted for Healthy Eating Index 2015 (HEI-2015) score excluding the sodium component; for potassium intake and the sodium-potassium ratio, models were additionally adjusted for HEI-2015 components for sodium (potassium model only), seafood and plant protein, saturated fat, fatty acids and refined grains. P for trend was calculated based on statistical significance of the coefficient of the category variable (median value assigned for each category).

**Table S7.** Risk of Overall and Cause-Specific Mortality Associated with per 1-SD Sodium-Potassium Ratio Among 237,036 Men and 179,068 Women ^a^

| **Sex, Intake and Causes** | **Per 1-s.d.** | **P value**  **(Linear)** | **P value**  **(Non-linear)** |
| --- | --- | --- | --- |
|  | **HR (95% CI)** |  |  |
| **Men** |  |  |  |
| Overall mortality | 1.04 (1.03, 1.05) | <0.0001 | <0.0001 |
| CVD | 1.04 (1.02, 1.06) | <0.0001 | 0.0003 |
| Heart disease | 1.04 (1.02, 1.06) | 0.0007 | 0.002 |
| Stroke | 1.08 (1.02, 1.13) | 0.0063 | 0.13 |
| Cancer | 1.01 (1.00, 1.03) | 0.14 | 0.94 |
| Respiratory disease | 1.11 (1.06, 1.15) | <0.0001 | 0.09 |
| Infectious disease | 0.98 (0.92, 1.03) | 0.41 | 0.07 |
| Injury and accidents | 1.05 (0.99, 1.11) | 0.12 | 0.12 |
| Other causes | 1.06 (1.04, 1.09) | <0.0001 | 0.06 |
| **Women** |  |  |  |
| Overall mortality | 1.07 (1.06, 1.09) | <0.0001 | 0.04 |
| CVD | 1.11 (1.08, 1.14) | <0.0001 | 0.12 |
| Heart disease | 1.12 (1.08, 1.15) | <0.0001 | 0.16 |
| Stroke | 1.06 (1.00, 1.13) | 0.063 | 0.19 |
| Cancer | 1.02 (0.99, 1.04) | 0.19 | 0.44 |
| Respiratory disease | 1.14 (1.09, 1.19) | <0.0001 | 0.32 |
| Infectious disease | 1.09 (1.02, 1.17) | 0.014 | 0.61 |
| Injury and accidents | 1.07 (0.98, 1.18) | 0.12 | 0.61 |
| Other causes | 1.08 (1.05, 1.12) | <0.0001 | 0.33 |

^a^ Hazard ratios (HRs) and their 95% confidence intervals (CIs) for mortality comparing the highest category with the referent (referent for sodium: the second lowest category, and referent for potassium and sodium-potassium: the lowest category). Multivariable analyses were adjusted for age at baseline, BMI, alcohol consumption, smoking status (never, former, current or missing), physical activity, race or ethnic group, education, marital status, diabetes (yes vs. no), health status, vitamin supplement use, and total energy intake. For sodium intake, models were additionally adjusted for Healthy Eating Index 2015 (HEI-2015) score excluding the sodium component; for potassium intake and the sodium-potassium ratio, models were additionally adjusted for HEI-2015 components for sodium (potassium model only), seafood and plant protein, saturated fat, fatty acids and refined grains. P for trend was calculated based on statistical significance of the coefficient of the category variable (median value assigned for each category).

**Table S8.** Search Strategy for Systematic Review *

| Pubmed | | | Embase | | |
| --- | --- | --- | --- | --- | --- |
| No. | Query | Result | No. | Query | Result |
| #1 | Case-cohort OR Case-control OR Cohort OR Follow-up OR Longitudinal OR Nested OR Prospective | 4,214,441 | #1 | ('prospective study' OR 'cohort study' OR 'case control study' OR 'longitudinal study' OR 'follow up' OR 'case-cohort' OR 'case-control') AND [embase]/lim | 3,642,348 |
| #2 | “Sodium, Dietary”[Mesh] OR “Sodium Chloride, Dietary”[Mesh] OR “urinary sodium excretion” | 19,940 | #2 | ('sodium intake'/exp OR 'dietary sodium' OR 'intake, sodium' OR 'natrium intake' OR 'sodium intake' OR 'sodium, dietary' OR 'sodium urine level'/exp OR 'diuresis, saline' OR 'diuresis, sodium' OR 'renal sodium excretion' OR 'sodium diuresis' OR 'sodium excretion, urinary' OR 'sodium loss' OR 'sodium urine level' OR 'urinary salt' OR 'urinary sodium' OR 'urinary sodium excretion' OR 'urine salt' OR 'urine sodium' OR 'salt intake'/exp OR 'dietary salt' OR 'dietary sodium chloride' OR 'intake, salt' OR 'salt intake' OR 'sodium chloride intake' OR 'sodium chloride, dietary') | 44,091 |
| #3 | “Cardiovascular Diseases” [Mesh] OR Cardiovascular OR CHD OR Coronary Heart Disease OR Myocardial Infarction OR CVD OR Haemorrhagic Stroke OR Hemorrhagic Stroke OR Heart Failure OR Ischemic Heart Disease OR Ischaemic Heart Disease OR Ischaemic stroke OR Ischemic Stroke OR “Stroke” [Mesh] | 3,842,935 | #3 | ('cardiovascular disease'/exp OR cardiovascular.ab. OR 'ischemic heart disease.ab.' OR 'coronary artery disease.ab.' OR 'cerebrovascular accident' OR 'chd.ab.' OR 'cvd.ab.' OR 'coronary heart disease.ab.' OR 'haemorrhagic stroke.ab.' OR 'hemorrhagic stroke.ab.' OR 'heart failure.ab.' OR 'ischaemic heart disease.ab.' OR 'ischaemic stroke.ab.' OR 'ischemic stroke.ab.' OR 'myocardial infarction' OR 'stroke.ab.') AND [embase]/lim | 4,578,581 |
| #4 | human | 22,141,925 | #4 | 'human' AND [embase]/lim | 20,172,074 |
| #7 | #1 AND #2 AND #3 AND #4 | 923 | #7 | (#1 AND #2 AND #3 AND #4) | 2,227 |
| Web of Science | | |  |  |  |
| No. | Query | Result |  |  |  |
| #1 | (TS=((Case-cohort or Case-control or Cohort or Follow-up or Longitudinal or Nested or Prospective) AND ("urinary sodium" or " sodium intake") AND (Cardiovascular or CHD or Coronary Heart Disease or CVD or Heart Failure or Ischaemic Heart Disease or Ischemic Heart Disease or Myocardial Infarction or Stroke))) AND DOCUMENT TYPES: (Article) | 991 |  |  |  |

* Search date: 2023-06-08.

**Table S9.** Studies included in the meta-analysis

| Study | PMID |
| --- | --- |
| Hu et al., 1992, stroke | PMID: 1519277 |
| He et al., 1999, Nonoverweight, CVD | PMID: 10591385 |
| Nagata et al., 2004, Men, stroke | PMID: 15143292 |
| Cohen et al., 2006, CVD death | PMID: 16490476 |
| Larsson et al., 2008, Cerebral infarction | PMID: 18332289 |
| Takachi et al., 2010, CVD | PMID: 20016010 |
| O'Donnell et al., 2011, CVD | PMID: 22110105 |
| Stolarz-Skrzypek et al., 2011, CVD | PMID: 21540421 |
| Yang et al., 2011, Men, CVD death | PMID: 21747015 |
| Gardener et al., 2012, CVD death | PMID: 22499576 |
| lkehara etal., 2012, Men, CVD death | PMID: 22057056 |
| Horikawa et al., 2014, CVD | PMID: 25050990 |
| Joosten et al., 2014, CHD | PMID: 24425751 |
| Pfister et al., 2014, Men HF | PMID: 24464931 |
| Kalogeropoulos et al., Men, 2015,CVD | PMID: 25599120 |
| Singer et al, 2015, CVD death | PMID: 25159082 |
| Mills et al., 2016, CVD | PMID: 27218629 |
| Olde-Enaberink et al., 2017, CVD | PMID: 28655835 |
| Kieneker et al., 2018, stroke | PMID: 30244812 |
| Liu et al., 2018, CVD | PMID: 30127349 |
| Kondo et al., 2019, CVD death | PMID: 31006729 |
| O'Donnell et al., 2019, CVD | PMID: 30867146 |
| Sadanaga et al., 2019, CVD | PMID: 30518986 |
| Elliott et al., 2020, CVD | PMID: 32623924 |
| Vuori et el., 2020, CVD | PMID: 32602794 |
| Nohara-Shitama et al., 2021, CVD death | PMID: 34039467 |
| Pickering et al., 2021, CVD | PMID: 33477824 |
| Wang et al. 2021, CVD | PMID: 34039459 |
| Wuopio et al., 2021, Men, AF | PMID: 33210391 |
| Ma et al. 2022, CVD | PMID: 34767706 |
| Wang et al., 2022, CVD | PMID: 35268096 |
| Kwon et al., 2022, CVD death | PMID: 36438773 |
| Mosallanezhad et al., 2023, CVD | PMID: 37072769 |

**Table S10.** Study Characteristics in the Meta-Analysis.

| **Cardiovascular disease** | | | | | | | | | | | | | | | | | | | | | | |
| --- | --- | --- | --- | --- | --- | --- | --- | --- | --- | --- | --- | --- | --- | --- | --- | --- | --- | --- | --- | --- | --- | --- |
| **Author, year** | **Study Population** | | **Country** | | **Follow-up (y)** | | **Sample Size** | | **Age Range** | | **Sodium Assessment** | | **Ascertainment of Outcomes** | | **Endpoints (No. of cases)** | | **Categories of Exposure** | | **Relative Risks (95% CI)*** | | | **Covariates in Multivariable Model** |
| Hu et al., 1992 no dose | Cohort of Taiwan | | China | | 4 yrs | | 8562 men and women | | >35 yrs | | Household survey questionnaire; | | The medical records | | Stroke (104) | | no salty food | | 1.00 (reference) | | | Age |
|  |  |  |  |  |  |  |  |  |  |  |  |  |  |  |  |  | yes salty food | | 1.79 (1.18, 2.71) | | |  |
| He et al., 1999 | NHANES I | | USA | | 19 yrs | | Nonoverweight: 6797 men and women | | 25-75 ys | | 24-hour dietary recall; | | Linking death records from National Death Index | | CVD death (566) | | 50.5 mmol/d | | 1.00 (reference) | | | Age, sex, race, BMI, PA, education, drinking, smoking, energy intake, SBP, TC, history of DM, diuretic use |
|  |  |  |  |  |  |  |  |  |  |  |  |  |  |  |  |  | 76.9 mmol/d | | 1.28 (0.99, 1.67) | | |  |
|  |  |  |  |  |  |  |  |  |  |  |  |  |  |  |  |  | 99.1 mmol/d | | 1.04 (0.83, 1.31) | | |  |
|  |  |  |  |  |  |  |  |  |  |  |  |  |  |  |  |  | 142.5 mmol/d | | 1.18 (0.92, 1.51) | | |  |
|  |  |  |  |  |  |  |  |  |  |  |  |  |  |  | CHD (1080) | | 50.5 mmol/d | | 1.00 (reference) | | |  |
|  |  |  |  |  |  |  |  |  |  |  |  |  |  |  |  |  | 76.9 mmol/d | | 1.34 (1.15, 1.57) | | |  |
|  |  |  |  |  |  |  |  |  |  |  |  |  |  |  |  |  | 99.1 mmol/d | | 1.05 (0.89, 1.24) | | |  |
|  |  |  |  |  |  |  |  |  |  |  |  |  |  |  |  |  | 142.5 mmol/d | | 1.06 (0.90, 1.25) | | |  |
|  |  |  |  |  |  |  |  |  |  |  |  |  |  |  | Stroke (430) | | 50.5 mmol/d | | 1.00 (reference) | | |  |
|  |  |  |  |  |  |  |  |  |  |  |  |  |  |  |  |  | 76.9 mmol/d | | 1.05 (0.80, 1.38) | | |  |
|  |  |  |  |  |  |  |  |  |  |  |  |  |  |  |  |  | 99.1 mmol/d | | 0.93 (0.70, 1.22) | | |  |
|  |  |  |  |  |  |  |  |  |  |  |  |  |  |  |  |  | 142.5 mmol/d | | 0.95 (0.75, 1.22) | | |  |
|  |  |  |  |  |  |  | Overweight: 2688 men and women | | 25-75 ys | | 24-hour dietary recall; | | Linking death records from National Death Index | | CVD death (329) | | 45.5 mmol/d | | 1.00 (reference) | | |  |
|  |  |  |  |  |  |  |  |  |  |  |  |  |  |  |  |  | 69.4 mmol/d | | 1.01 (0.68, 1.49) | | |  |
|  |  |  |  |  |  |  |  |  |  |  |  |  |  |  |  |  | 88.7 mmol/d | | 1.28 (0.89, 1.85) | | |  |
|  |  |  |  |  |  |  |  |  |  |  |  |  |  |  |  |  | 129.7 mmol/d | | 1.63 (1.16, 2.27) | | |  |
|  |  |  |  |  |  |  |  |  |  |  |  |  |  |  | CHD (647) | | 45.5 mmol/d | | 1.00 (reference) | | |  |
|  |  |  |  |  |  |  |  |  |  |  |  |  |  |  |  |  | 69.4 mmol/d | | 0.96 (0.78, 1.19) | | |  |
|  |  |  |  |  |  |  |  |  |  |  |  |  |  |  |  |  | 88.7 mmol/d | | 1.00 (0.80, 1.25) | | |  |
|  |  |  |  |  |  |  |  |  |  |  |  |  |  |  |  |  | 129.7 mmol/d | | 0.97 (0.77, 1.21) | | |  |
|  |  |  |  |  |  |  |  |  |  |  |  |  |  |  | stroke (250) | | 45.5 mmol/d | | 1.00 (reference) | | |  |
|  |  |  |  |  |  |  |  |  |  |  |  |  |  |  |  |  | 69.4 mmol/d | | 1.28 (0.86, 1.89) | | |  |
|  |  |  |  |  |  |  |  |  |  |  |  |  |  |  |  |  | 88.7 mmol/d | | 1.64 (1.07, 2.52) | | |  |
|  |  |  |  |  |  |  |  |  |  |  |  |  |  |  |  |  | 129.7 mmol/d | | 1.51 (1.06, 2.14) | | |  |
| Nagata et al., 2004 | Takayama study | | Japan | | 7 yrs | | 13355 men | | 51.0 yrs | | Food frequency questionnaire; | | Confirmed with data from National Vital Statistics. | | Total stroke (137) | | 4070 mg/d | | 1.00 (reference) | | | Age, energy intake, marital status, education, BMI, smoking, drinking, PA, histories of HTN and DM, protein, K and vitamin E |
|  |  |  |  |  |  |  |  |  | 53.2 yrs | |  |  |  |  |  |  | 5209 mg/d | | 1.60 (0.92, 2.80) | | |  |
|  |  |  |  |  |  |  |  |  | 57.7 yrs | |  |  |  |  |  |  | 6613 mg/d | | 2.33 (1.23, 4.45) | | |  |
|  |  |  |  |  |  |  | 15724 women | | 53.3 yrs | | Food frequency questionnaire; | | Confirmed with data from National Vital Statistics. | | Total stroke (132) | | 3799 mg/d | | 1.00 (reference) | | |  |
|  |  |  |  |  |  |  |  |  | 54.3 yrs | |  |  |  |  |  |  | 4801 mg/d | | 1.33 (0.80, 2.21) | | |  |
|  |  |  |  |  |  |  |  |  | 57.8 yrs | |  |  |  |  |  |  | 5930 mg/d | | 1.70 (0.96, 3.02) | | |  |
| Cohen et al., 2006 | NHANES II | | USA | | 13.7 yrs | | 7154 men and women | | 30-74 yrs | | 24-hour dietary recall; | | Linking death records from National Death Index | | CVD death (541) | | <2300mg/d | | 1.37 (1.03, 1.81) | | | Age, sex, race, smoking, alcohol use, systolic blood pressure, anti-hypertensive treatment, body mass index, education <high school, physical activity, body mass index, dietary potassium, history of diabetes, serum cholesterol and calories |
|  |  |  |  |  |  |  |  |  |  |  |  |  |  |  |  |  | ≥2300 mg/d | | 1.00 (reference) | | |  |
| Larsson et al., 2008 | ATBC cohort | | Sweden | | 13.6 yrs | | 26556 men | | 50-69 yrs | | Food frequency questionnaire; | | Linking with the National Hospital Discharge Register and the National Register of Causes of Death | | Cerebral infarction (2702) | | 3909 mg/d | | 1.00 (reference) | | | Age, supplementation group, smoking, drinking, BMI, SBP, DBP, TC, HDL-C, histories of DM, CHD, PA, energy intake |
|  |  |  |  |  |  |  |  |  |  |  |  |  |  |  |  |  | 4438 mg/d | | 1.08 (0.96, 1.22) | | |  |
|  |  |  |  |  |  |  |  |  |  |  |  |  |  |  |  |  | 4810 mg/d | | 1.05 (0.93, 1.18) | | |  |
|  |  |  |  |  |  |  |  |  |  |  |  |  |  |  |  |  | 5212 mg/d | | 0.99 (0.87, 1.13) | | |  |
|  |  |  |  |  |  |  |  |  |  |  |  |  |  |  |  |  | 5848 mg/d | | 1.04 (0.92, 1.18) | | |  |
| Takachi et al., 2010 | Cohort I and cohort II of the Japan Public Health Center-based Prospective Study | | Japan | | 6-9 yrs | | 77500 men and women | | 45-74 yrs | | Food frequency questionnaire; | | Linking to the medical record | | CVD (2066) | | 3084 mg/d | | 1.00 (reference) | | | Age, sex, BMI, smoking, drinking, PA, energy, K and Ca intake |
|  |  |  |  |  |  |  |  |  |  |  |  |  |  |  |  |  | 4005 mg/d | | 1.11 (0.96, 1.29) | | |  |
|  |  |  |  |  |  |  |  |  |  |  |  |  |  |  |  |  | 4709 mg/d | | 1.02 (0.87, 1.19) | | |  |
|  |  |  |  |  |  |  |  |  |  |  |  |  |  |  |  |  | 5503 mg/d | | 1.10 (0.94, 1.29) | | |  |
|  |  |  |  |  |  |  |  |  |  |  |  |  |  |  |  |  | 6844 mg/d | | 1.19 (1.01, 1.40) | | |  |
| O'Donnell et al., 2011 | ONTARGET/TRANSCEND | | 40 countries | | 4.67 yrs | | 28880 men and women | | 66.5 yrs | | ﻿24-hour urinary sodium excretion; | | Using standardized criteria, by a blinded central committee | | Total CVD mortality † (4729) | | <2 g/d | | 1.21 (1.03, 1.43) | | | Age, sex, race/ethnicity (white vs nonwhite), prior history of stroke or myocardial infarction, creatinine, body mass index, comorbid vascular risk factors (hypertension, diabetes mellitus, atrial fibrillation, smoking, low-density lipoprotein, and high-density lipoprotein), treatment allocation (ramipril, telmisartan, or both, and treatment with statins, beta-blockers, diuretic therapy, calcium antagonist, and antithrombotic therapy), fruit and vegetable consumption, level of exercise, baseline blood pressure and change in systolic blood pressure from baseline to last follow-up, and urinary potassium. |
|  |  |  |  |  |  |  |  |  |  |  |  |  |  |  |  |  | 2-2.99 g/d | | 1.16 (1.04, 1.28) | | |  |
|  |  |  |  |  |  |  |  |  |  |  |  |  |  |  |  |  | 3-3.99 g/d | | 1.06 (0.98, 1.14) | | |  |
|  |  |  |  |  |  |  |  |  |  |  |  |  |  |  |  |  | 4-5.99 g/d | | 1.00 (reference) | | |  |
|  |  |  |  |  |  |  |  |  |  |  |  |  |  |  |  |  | 6-6.99 g/d | | 1.09 (0.99, 1.20) | | |  |
|  |  |  |  |  |  |  |  |  |  |  |  |  |  |  |  |  | 7-8 g/d | | 1.15 (1.00, 1.32) | | |  |
|  |  |  |  |  |  |  |  |  |  |  |  |  |  |  |  |  | >8 g/d | | 1.49 (1.28, 1.75) | | |  |
| Stolarz-Skrzypek et al., 2011 | FLEMENGHO/EPOGH | | Belgium | | 7.9 yrs | | 3681 men and women | | 39.7 yrs | | ﻿24-hour urinary sodium excretion; | | Linking to the medical records or the death certifications | | Total CVD (232) include fatal and nonfatal | | 172.7 mmol/d | | 1.13 (0.90, 1.42) | | | Study population, sex, and baseline variables: age, body mass index, systolic blood pressure, 24-hour urinary potassium excretion, antihypertensive drug treatment, smoking and drinking alcohol, diabetes, total cholesterol, and educational attainment |
|  |  |  |  |  |  |  |  |  |  |  |  |  |  |  |  |  | 174.2 mmol/d | | 1.11 (0.90, 1.36) | | |  |
|  |  |  |  |  |  |  |  |  |  |  |  |  |  |  |  |  | 178 mmol/d | | 0.90 (0.73, 1.11) | | |  |
| Yang et al., 2011 | NHANES III | | USA | | 14.8 yrs | | 5899 men | | 25-74 yrs | | 24-hour dietary recall; | | Linking death records from National Death Index | | CVD death (437) | | 2908 mg/d | | 1.00 (reference) | | | Sex, race, education, BMI, smoking, drinking, TC, HDL- C, PA, family history of CVD, energy intake |
|  |  |  |  |  |  |  |  |  |  |  |  |  |  |  |  |  | 3785 mg/d | | 1.15 (0.83, 1.59) | | |  |
|  |  |  |  |  |  |  |  |  |  |  |  |  |  |  |  |  | 4570 mg/d | | 1.30 (0.70, 2.42) | | |  |
|  |  |  |  |  |  |  |  |  |  |  |  |  |  |  |  |  | 5751 mg/d | | 1.58 (0.55, 4.54) | | |  |
|  |  |  |  |  |  |  | 6368 women | | 25-74 yrs | | 24-hour dietary recall; | | Linking death records from National Death Index | | CVD death (388) | | 1893 mg/d | | 1.00 (reference) | | |  |
|  |  |  |  |  |  |  |  |  |  |  |  |  |  |  |  |  | 2549 mg/d | | 0.79 (0.53, 1.16) | | |  |
|  |  |  |  |  |  |  |  |  |  |  |  |  |  |  |  |  | 3148 mg/d | | 0.63 (0.30, 1.32) | | |  |
|  |  |  |  |  |  |  |  |  |  |  |  |  |  |  |  |  | 4068 mg/d | | 0.45 (0.12, 1.62) | | |  |
|  |  |  |  |  |  |  | 12,267 men and women | | 25-74 yrs | | 24-hour dietary recall; | | Linking death records from National Death Index | | CVD death (825) | | 1538 mg/d | | 1.00 (reference) | | |  |
|  |  |  |  |  |  |  |  |  |  |  |  |  |  |  |  |  | 2397 mg/d | | 0.82 (0.61, 1.09) | | |  |
|  |  |  |  |  |  |  |  |  |  |  |  |  |  |  |  |  | 3166 mg/d | | 0.65 (0.43, 0.96) | | |  |
|  |  |  |  |  |  |  |  |  |  |  |  |  |  |  |  |  | 4202 mg/d | | 0.87 (0.56, 1.35) | | |  |
|  |  |  |  |  |  |  |  |  |  |  |  |  |  |  |  |  | 6189 mg/d | | 0.74 (0.44, 1.26) | | |  |
| Gardener et al., 2012 | the Northern Manhattan Study | | USA | | 10 yrs | | 2657 men and women | | 69 yrs | | Food frequency questionnaire; | | Linking to the medical record | | CVD death (615) | | ≤1500 mg/day | | 1.00 (reference) | | | Age, sex, race, education, smoking, drinking, PA, energy, protein, fat, saturated fat and carbohydrates intake, DM, hypercholesterolemia, HTN, history of CVD, BMI |
|  |  |  |  |  |  |  |  |  |  |  |  |  |  |  |  |  | 1501–2300 | | 1.35 (1.00, 1.82) | | |  |
|  |  |  |  |  |  |  |  |  |  |  |  |  |  |  |  |  | 2301–3999 | | 1.21 (0.87, 1.67) | | |  |
|  |  |  |  |  |  |  |  |  |  |  |  |  |  |  |  |  | 4000–10000 | | 1.68 (1.06, 2.67) | | |  |
| Ikehara et al., 2012 | the JACC study | | Japan | | 16.4yrs | | 35515 men | | 40-79 yrs | | Self-administered questionnaire; | | Linking death records from the public health center in the area of residency | | CVD death (2318) | | 1878 mg/d | | 1.00 (reference) | | | Age, BMI, history of DM, HTN, smoking, drinking, education, PA, walking, mental stress, fresh fish intake |
|  |  |  |  |  |  |  |  |  |  |  |  |  |  |  |  |  | 2083 mg/d | | 1.02 (0.90, 1.16) | | |  |
|  |  |  |  |  |  |  |  |  |  |  |  |  |  |  |  |  | 2151 mg/d | | 1.05 (0.92, 1.20) | | |  |
|  |  |  |  |  |  |  | 49275 women | | 40-79 yrs | | Self-administered questionnaire; | | Linking death records from the public health center in the area of residency | | CVD death (2099) | | 1725 mg/d | | 1.00 (reference) | | |  |
|  |  |  |  |  |  |  |  |  |  |  |  |  |  |  |  |  | 1950 mg/d | | 0.96 (0.86, 1.08) | | |  |
|  |  |  |  |  |  |  |  |  |  |  |  |  |  |  |  |  | 2022 mg/d | | 1.05 (0.92, 1.19) | | |  |
| Horikawa et al., 2014 | the JDCS Study | | Japan | | 8yrs | | 1588 men and women | | 58.7 yrs | | Food frequency questionnaire; | | Linking to the medical records | | CVD (132) | | 3.3 g/d | | 1.00 (reference) | | | Age, sex, BMI, HbA1c, DM duration, LDL-C, HDL-C, TG, insulin, lipid-lowering agents, smoking, drinking, energy intake, PA, SBP, antihypertensive agents |
|  |  |  |  |  |  |  |  |  |  |  |  |  |  |  |  |  | 3.8 g/d | | 1.73 (1.00, 3.00) | | |  |
|  |  |  |  |  |  |  |  |  |  |  |  |  |  |  |  |  | 4.4 g/d | | 1.58 (0.88, 2.84) | | |  |
|  |  |  |  |  |  |  |  |  |  |  |  |  |  |  |  |  | 4.6 g/d | | 2.17 (1.21, 3.90) | | |  |
| Joosten et al., 2014 | the PREVEND Study | | Netherlands | | 10.5yrs | | 7543 men and women | | 28-75 yrs | | ﻿24-hour urinary sodium excretion; | | Linking medical records from the Dutch national registry of hospital discharge diagnoses and death records from the Dutch Central Bureau of Statistics | | CHD (452) | | Men: <122 mmol/d; women: <95 mmol/d | | 1.00 (reference) | | | Age, sex, BMI, smoking, drinking, family history of CHD, DM, TC/HDL-C, urine Mg, K, Cr |
|  |  |  |  |  |  |  |  |  |  |  |  |  |  |  |  |  | Men: 122-154 mmol/d; women: 95-121 mmol/d | | 0.99 (0.76, 1.29) | | |  |
|  |  |  |  |  |  |  |  |  |  |  |  |  |  |  |  |  | Men: 155-190 mmol/d; women: 122-151 mmol/d | | 1.09 (0.83, 1.44) | | |  |
|  |  |  |  |  |  |  |  |  |  |  |  |  |  |  |  |  | Men: >190 mmol/d; women: >151 mmol/d | | 1.19 (0.88, 1.62) | | |  |
| Pfister et al., 2014 | the EPIC-Norfolk study | | United Kingdom | | 12.9yrs | | 9017 men | | 39-79 yrs | | Single spot urine sodium excretion; | | the death certificate data and hospital record | | HF (800) | | 107 mmol/d | | 1.23 (0.99, 1.52) | | | Age, BMI, DM, TC, social class, education, smoking, drinking, PA, BP |
|  |  |  |  |  |  |  |  |  |  |  |  |  |  |  |  |  | 138 mmol/d | | 1.00 (reference) | | |  |
|  |  |  |  |  |  |  |  |  |  |  |  |  |  |  |  |  | 158 mmol/d | | 0.98 (0.78, 1.23) | | |  |
|  |  |  |  |  |  |  |  |  |  |  |  |  |  |  |  |  | 178 mmol/d | | 0.95 (0.75, 1.20) | | |  |
|  |  |  |  |  |  |  |  |  |  |  |  |  |  |  |  |  | 217 mmol/d | | 1.05 (0.84, 1.32) | | |  |
|  |  |  |  |  |  |  | 10840 women | | 39-79 yrs | | Single spot urine sodium excretion; | | the death certificate data and hospital record | | HF (613) | | 107 mmol/d | | 1.03 (0.80, 1.32) | | |  |
|  |  |  |  |  |  |  |  |  |  |  |  |  |  |  |  |  | 138 mmol/d | | 1.00 (reference) | | |  |
|  |  |  |  |  |  |  |  |  |  |  |  |  |  |  |  |  | 158 mmol/d | | 0.82 (0.62, 1.07) | | |  |
|  |  |  |  |  |  |  |  |  |  |  |  |  |  |  |  |  | 178 mmol/d | | 0.94 (0.72, 1.21) | | |  |
|  |  |  |  |  |  |  |  |  |  |  |  |  |  |  |  |  | 217 mmol/d | | 1.17 (0.92, 1.50) | | |  |
| Kalogero pouloset al., 2015 | The Health ABC Study | | USA | | 10 yrs | | 900 men | | 71-80 yrs | | Food frequency questionnaire; | | Linking to hospital records, interviews, and death certificates | | CVD (312) | | <1500 mg/d | | 1.13 (0.72, 1.77) | | | Age, sex, race, HTN, BMI, smoking, PA, history of CVD, pulmonary disease, DM, depression, BP, HR, EKG, serum glucose, Alb, Cr, TC |
|  |  |  |  |  |  |  |  |  |  |  |  |  |  |  |  |  | 1500-2300 mg/d | | 1.00 (reference) | | |  |
|  |  |  |  |  |  |  |  |  |  |  |  |  |  |  |  |  | >2300 mg/d | | 0.91 (0.69, 1.18) | | |  |
|  |  |  |  |  |  |  | 1081 women | | 71-80 yrs | | Food frequency questionnaire; | | Linking to hospital records, interviews, and death certificates | | CVD women (260) | | <1500 mg/d | | 1.04 (0.71, 1.54) | | |  |
|  |  |  |  |  |  |  |  |  |  |  |  |  |  |  |  |  | 1500-2300 mg/d | | 1.00 (reference) | | |  |
|  |  |  |  |  |  |  |  |  |  |  |  |  |  |  |  |  | >2300 mg/d | | 1.12 (0.85, 1.49) | | |  |
| Singer et al., 2015 | A hypertension program in New York City | | USA | | 20 yrs | | 3505 men and women | | 52 yrs | | ﻿24-hour urinary sodium excretion; | | Linking death records from National Death Index Plus and the Social Security Administration Death Master File | | CVD death (399) | | 55 mmol/d | | 1.00 (0.71, 1.42) | | | Age, sex, race, BMI, SBP, eGFR, urine K, hematocrit, PRA, DM, smoking, history of baseline LVH |
|  |  |  |  |  |  |  |  |  |  |  |  |  |  |  |  |  | 102 mmol/d | | 0.96 (0.68, 1.36) | | |  |
|  |  |  |  |  |  |  |  |  |  |  |  |  |  |  |  |  | 143 mmol/d | | 1.06 (0.73, 1.55) | | |  |
|  |  |  |  |  |  |  |  |  |  |  |  |  |  |  |  |  | 221 mmol/d | | 1.00 (reference) | | |  |
| Mills et al., 2016 | the CRIC Study | | USA | | 6.8 yrs | | 3757 men and women | | 58 yrs | | ﻿24-hour urinary sodium excretion; | | Linking to the medical records | | CVD (804) | | <2894 mg/d | | 1.00 (reference) | | | Age, sex, race, clinic site, education, WC, lean BMI, BMI, smoking, drinking, PA; LDL-C, glucose, history of CVD, use of antidiabetic agents, lipid-lowering agents, diuretics, RAS blockades, antihypertensive agents, urinary Cr excretion, eGFR |
|  |  |  |  |  |  |  |  |  |  |  |  |  |  |  |  |  | 2894-3549 mg/d | | 0.87 (0.69, 1.10) | | |  |
|  |  |  |  |  |  |  |  |  |  |  |  |  |  |  |  |  | 3650-4547 mg/d | | 1.01 (0.81, 1.26) | | |  |
|  |  |  |  |  |  |  |  |  |  |  |  |  |  |  |  |  | ≥4548 mg/d | | 1.36 (1.09, 1.70) | | |  |
| Olde Engberink et al., 2017 | a cohort in Amsterdam | | Neatherlands | | 16.2 yrs | | 574 men and women | | 47 yrs | | ﻿24-hour urinary sodium excretion; | | Linking to the medical records | | CVD (406) | | <3.0 g/d | | 1.00 (reference) | | | Age, sex, race, eGFR, history of DM, kidney disease, CVD, HTN, smoking, 24-hour urine K, urine Cr, number of antihypertensive agents, the use of RAS blockades |
|  |  |  |  |  |  |  |  |  |  |  |  |  |  |  |  |  | 3-4.3 g/d | | 1.25 (0.72, 2.16) | | |  |
|  |  |  |  |  |  |  |  |  |  |  |  |  |  |  |  |  | >4.3 g/d | | 1.73 (1.00, 2.99) | | |  |
| Kieneker et al., 2018 | the PREVEND study | | Neatherlands | | 12.5 yrs | | 7330 men and women | | 49.2 yrs | | ﻿24-hour urinary sodium excretion; | | the municipal register and the Dutch national registry | | Stroke (183) | | 83 mmol/d | | 1.45 (0.92, 2.29) | | | Age, sex, height, weight, race, smoking, drinking, education, DM, TC/HDL-C, urine K, Mg, Cr, Alb, eGFR |
|  |  |  |  |  |  |  |  |  |  |  |  |  |  |  |  |  | 110 mmol/d | | 1.13 (0.71, 1.79) | | |  |
|  |  |  |  |  |  |  |  |  |  |  |  |  |  |  |  |  | 132 mmol/d | | 1.00 (reference) | | |  |
|  |  |  |  |  |  |  |  |  |  |  |  |  |  |  |  |  | 159 mmol/d | | 1.04 (0.64, 1.71) | | |  |
|  |  |  |  |  |  |  |  |  |  |  |  |  |  |  |  |  | 209 mmol/d | | 0.81 (0.46, 1.41) | | |  |
| Liu et al., 2018 | the PRC-USA Collaborative Study | | China | | 18.6yrs | | 954 men and women | | 46.4 yrs | | 8-hour urinary sodium excretion; | | Hospital records, autopsy results, and death certificates | | CVD (81) | | 35.1 mmol/8h | | 1.00 (reference) | | | Age, sex, area, work, smoking, drinking, TC, FBG, BMI, region, education, PA, percentage of energy from saturated fat, SBP, antihypertensive agents |
|  |  |  |  |  |  |  |  |  |  |  |  |  |  |  |  |  | 54.5 mmol/8h | | 1.66 (0.79, 3.47) | | |  |
|  |  |  |  |  |  |  |  |  |  |  |  |  |  |  |  |  | 84.1 mmol/8h | | 3.04 (1.46, 6.34) | | |  |
|  |  |  |  |  |  |  |  |  |  |  |  |  |  |  | CVD death (31) | | 35.1 mmol/8h | | 1.00 (reference) | | |  |
|  |  |  |  |  |  |  |  |  |  |  |  |  |  |  |  |  | 54.5 mmol/8h | | 1.08 (0.36, 3.30) | | |  |
|  |  |  |  |  |  |  |  |  |  |  |  |  |  |  |  |  | 84.1 mmol/8h | | 1.90 (0.64, 5.63) | | |  |
| Kondo et al., 2019 | NIPPON DATA | | Japan | | 29 yrs | | 9115 men and women | | 50 yrs | | 3-day dietary records; | | Linking to the National Vital Statistics database of Japan | | CVD death (1070) | | Low: men: <8 g/d, women<7 g/d | | 1.00 (reference) | | | Age, sex, smoking, drinking, energy intake |
|  |  |  |  |  |  |  |  |  |  |  |  |  |  |  |  |  | High: men≥8 g/d, women≥7 g/d | | 1.35 (1.02, 1.79) | | |  |
| O'Donnell et al., 2019 | The PURE Study | | Argentina, Bangladesh, Brazil, Canada, Chile, China, Colombia, India, Iran, Malaysia, Pakistan, Palestinian territories occupied by Israel, Poland, South Africa, Sweden, Turkey, United Arab Emirates and Zimbabwe | | 8.2 yrs | | 104570 men and women | | 51 yrs | | ﻿24-hour urinary sodium excretion; | | Death certificates, medical records, household interviews and other sources. | | Total CVD (7884) | | <3 g/d | | 1.16 (1.03, 1.31) | | | Age, sex, education, drinking, smoking, DM, BMI, PA, history of CVD, cancer, TB, CVD agents, cancer, HIV |
|  |  |  |  |  |  |  |  |  |  |  |  |  |  |  |  |  | 3-3.99 g/d | | 1.04 (0.94, 1.15) | | |  |
|  |  |  |  |  |  |  |  |  |  |  |  |  |  |  |  |  | 4-4.99 g/d | | 1.00 (reference) | | |  |
|  |  |  |  |  |  |  |  |  |  |  |  |  |  |  |  |  | 5-5.99 g/d | | 1.08 (0.98, 1.19) | | |  |
|  |  |  |  |  |  |  |  |  |  |  |  |  |  |  |  |  | 6-6.99 g/d | | 1.06 (0.95, 1.18) | | |  |
|  |  |  |  |  |  |  |  |  |  |  |  |  |  |  |  |  | ≥7 g/d | | 1.12 (0.99, 1.25) | | |  |
| Sadanaga et al., 2019 | the ESPRIT Study | | Japan | | 5.2 yrs | | 520 men and women | | 73 yrs | | ﻿24-hour urinary sodium excretion; | | Linking to the medical records | | CVD (105) include fatal and nonfatal | | <3 g/d | | 0.97 (0.55, 1.69) | | | Age, gender, and body weight |
|  |  |  |  |  |  |  |  |  |  |  |  |  |  |  |  |  | 3.0-3.49 g/d | | 1.00 (reference) | | |  |
|  |  |  |  |  |  |  |  |  |  |  |  |  |  |  |  |  | 3.5-3.99 g/d | | 1.05 (0.61, 1.79) | | |  |
|  |  |  |  |  |  |  |  |  |  |  |  |  |  |  |  |  | ≥ 4.0 g/d | | 1.79 (1.01, 3.15) | | |  |
| Elliott et al., 2020 | UK Biobank | | UK | | 5.9 yrs | | 398629 men and women | | 40-69 yrs | | ﻿24-hour urinary sodium excretion; | | Linking to the medical records and the Office for National Statistics | | CVD (11451) | | 2.51 g/d | | 0.99 (0.93, 1.05) | | | Potassium excretion, alcohol intake, smoking (current, past, never), sedentary lifestyle (hours sitting watching television, use of computer or sitting), Townsend deprivation index, body mass index and blood pressure medications |
|  |  |  |  |  |  |  |  |  |  |  |  |  |  |  |  |  | 3.21 g/d | | 1.00 (reference) | | |  |
|  |  |  |  |  |  |  |  |  |  |  |  |  |  |  |  |  | 4.21 g/d | | 1.04 (0.98, 1.10) | | |  |
| Vuoro et al., 2020 | the North Karelia Salt Project in 1979, WHO FINMONICAstudiesin1982, 1987 and the National FINRISK Study in 2002 | | Finland | | 14 yrs | | 4630 men and women | | 25-64 yrs | | ﻿24-hour urinary sodium excretion; | | Linking to the National Causes of Death Register, the Finnish Hospital Discharge Register and the Drug Reimbursement Register | | CVD (424) | | 97.1 mmol/d | | 0.70 (0.51, 0.95) | | | Age, BMI, cholesterol, prevalent diabetes and stratified by sex and cohort |
|  |  |  |  |  |  |  |  |  |  |  |  |  |  |  |  |  | 149.0 mmol/d | | 0.70 (0.53, 0.93) | | |  |
|  |  |  |  |  |  |  |  |  |  |  |  |  |  |  |  |  | 196.6 mmol/d | | 0.73 (0.57, 0.94) | | |  |
|  |  |  |  |  |  |  |  |  |  |  |  |  |  |  |  |  | 289.5 mmol/d | | 1.00 (reference) | | |  |
| Nohara-Shitama et al., 2021 | The Tanushimaru survey | | Japan | | 27.5 yrs | | 1291 men and women | | 21-85 yrs | | ﻿24-hour urinary sodium excretion; | | Confirmed based on a review of obituaries, medical records, death certificates, hospital charts, and interviews with primary care physicians, families of the deceased and other witnesses | | CVD death (142) | | 0.32-4.24 g/d | | 1.00 (reference) | | | Age, sex, body mass index, FPG, triglycerides, serum albumin, and systolic BP |
|  |  |  |  |  |  |  |  |  |  |  |  |  |  |  |  |  | 4.25-5.55 g/d | | 0.72 (0.47, 1.12) | | |  |
|  |  |  |  |  |  |  |  |  |  |  |  |  |  |  |  |  | 5.56-7.04 g/d | | 0.61 (0.37, 1.00) | | |  |
|  |  |  |  |  |  |  |  |  |  |  |  |  |  |  |  |  | 7.05-18.2 g/d | | 0.46 (0.25, 0.85) | | |  |
| Pickering et al., 2021 | the Framingham Offspring Study | | USA | | 19.7 yrs | | 2362 men and womem | | 30-64 yrs | | 3-day dietary records; | | Confirmed by three investigators | | CVD (404) | | <2500 mg/d | | 1.00 (reference) | | | Age, sex, BMI, prevalent hypertension and dietary fiber |
|  |  |  |  |  |  |  |  |  |  |  |  |  |  |  |  |  | 2500-3500 mg/d | | 1.12 (0.88, 1.43) | | |  |
|  |  |  |  |  |  |  |  |  |  |  |  |  |  |  |  |  | ≥3500 mg/d | | 1.06 (0.80, 1.39) | | |  |
| Wang et al., 2021 | the Chin-Shan Community Cardiovascular Cohort Study | | China | | 14.1 yrs | | 2112 men and women | | 54.5 yrs | | ﻿24-hour urinary sodium excretion; | | Confirmed by reviewing the questionnaires, medical records, death certificates and laboratory data | | CVD (279) | | <2 g/d | | 1.00 (reference) | | | Age groups (35–44, 45–54, 55–64, 65–74 and ≥75 years of age) , sex, BMI (<18, 18–20·9, 21–22·9, 23–24·9 or ≥25 kg/m2), smoking (yes/no or abstinence), current alcohol drinking (regular/no), marital status (single, married and living with a spouse, or divorced and separated), regular exercise habits (yes/no), education level (<9 years, at least 9 years) , occupation (no work, labour, official or business), LDL (mg/dl), glomerular filtration rate (<60, ≥60 ml/min per 1·73 m2) and diabetes status (yes/no) |
|  |  |  |  |  |  |  |  |  |  |  |  |  |  |  |  |  | 2.0-2.9 g/d | | 1.03 (0.73, 1.47) | | |  |
|  |  |  |  |  |  |  |  |  |  |  |  |  |  |  |  |  | 2.9-4.2 g/d | | 0.92 (0.64, 1.32) | | |  |
|  |  |  |  |  |  |  |  |  |  |  |  |  |  |  |  |  | >4.2 g/d | | 1.43 (1.02, 1.99) | | |  |
| Wuopio et al., 2021 | UK biobank | | UK | | 8.2 yrs | | 215535 men | | 40-69 yrs | | ﻿24-hour urinary sodium excretion; | | Linking to the medical records and the Office for National Statistics | | AF (3751) | | 2.8 g/d | | 1.20 (1.08, 1.32) | | | Age, ethnicity, hypertension, smoking, BMI, diabetes mellitus, ongoing alcohol abuse, total cholesterol and eGFR-creatinine |
|  |  |  |  |  |  |  |  |  |  |  |  |  |  |  |  |  | 3.78 g/d | | 1.08 (0.98, 1.20) | | |  |
|  |  |  |  |  |  |  |  |  |  |  |  |  |  |  |  |  | 4.4 g/d | | 1.00 (reference) | | |  |
|  |  |  |  |  |  |  |  |  |  |  |  |  |  |  |  |  | 5.05 g/d | | 1.09 (0.98, 1.21) | | |  |
|  |  |  |  |  |  |  |  |  |  |  |  |  |  |  |  |  | 6.26 g/d | | 1.15 (1.03, 1.27) | | |  |
|  |  |  |  |  |  |  | 257545 women | | 40-69 yrs | | ﻿24-hour urinary sodium excretion; | | Linking to the medical records and the Office for National Statistics | | AF (2221) | | 2.4 g/d | | 1.05 (0.92, 1.19) | | |  |
|  |  |  |  |  |  |  |  |  |  |  |  |  |  |  |  |  | 3.2 g/d | | 0.93 (0.81, 1.07) | | |  |
|  |  |  |  |  |  |  |  |  |  |  |  |  |  |  |  |  | 3.8 g/d | | 1.00 (reference) | | |  |
|  |  |  |  |  |  |  |  |  |  |  |  |  |  |  |  |  | 4.3 g/d | | 1.03 (0.90, 1.18) | | |  |
|  |  |  |  |  |  |  |  |  |  |  |  |  |  |  |  |  | 5.4 g/d | | 1.02 (0.89, 1.16) | | |  |
| Ma et al., 2022 | the HPFS, the NHS, the NHS II, the PREVEND study, and the TOHP I and TOHP II Studies | | USA | | 8.8 yrs | | 10709 men and women | | 51.5 yrs | | ﻿24-hour urinary sodium excretion; | | Linking to the medical records and death records from National Death Index | | CVD (571) | | 2212 mg/d | | 1.00 (reference) | | | Age, sex, race, educational level, height, body-mass index, smoking status, alcohol consumption, physical activity, history of diabetes and elevated cholesterol status, family history of cardiovascular disease, the 24-hour urinary potassium excretion (for sodium as the exposure) and 24-hour sodium excretion (for potassium as the exposure), and total energy intake and the modified DASH (Dietary Approaches to Stop Hypertension) diet quality score for the HPFS, NHS, and NHS II |
|  |  |  |  |  |  |  |  |  |  |  |  |  |  |  |  |  | 2942 mg/d | | 1.25 (0.96, 1.63) | | |  |
|  |  |  |  |  |  |  |  |  |  |  |  |  |  |  |  |  | 3588 mg/d | | 1.44 (0.93, 2.23) | | |  |
|  |  |  |  |  |  |  |  |  |  |  |  |  |  |  |  |  | 4692 mg/d | | 1.60 (1.19, 2.14) | | |  |
| Wang et al., 2022 | the MVP | | USA | | 8 yrs | | 180156 men | | 19-107 yrs | | Food frequency questionnaire; | | Linking to the VA EHR, Centers for Medicaid & Medicare Services database, and National Death Index database | | CVD (4090) | | 898 mg/d | | 1.00 (reference) | | | Age (continuous), sex (male or female) adjusted relative risk, race/ethnicity (European Americans or other), education level (≤high school or GED, some colleague, or college or above), income level (<$30,000, $30,000–$59,000 or ≥$60,000) and marital status (currently married or not), smoking status (current, former or never smoking), frequency of alcohol consumption (never, <1 times/week or ≥1 times/week), frequency of exercise vigorously (never/rarely, 1–4 times/month, 2–4 times/week, or ≥5 times/week), total energy intake (in quintiles), body mass index (continuous), the presences of diabetes, high cholesterol, and hypertension at baseline (each Yes vs. No) |
|  |  |  |  |  |  |  |  |  |  |  |  |  |  |  |  |  | 1095 mg/d | | 0.96 (0.86, 1.06) | | |  |
|  |  |  |  |  |  |  |  |  |  |  |  |  |  |  |  |  | 1231 mg/d | | 0.97 (0.88, 1.08) | | |  |
|  |  |  |  |  |  |  |  |  |  |  |  |  |  |  |  |  | 1373 mg/d | | 1.04 (0.94, 1.16) | | |  |
|  |  |  |  |  |  |  |  |  |  |  |  |  |  |  |  |  | 1605 mg/d | | 1.09 (0.99, 1.21) | | |  |
| Kwon et al., 2022 | | the KOGES study | | Korea | | 10 yrs | | 143,050 men and women | | 53.5 yrs | | Food frequency questionnaire | | Linked to national data sources (Korea National Statistical Office) | | CVD death (985) | | 19.7-1363.9 mg/day | | | 1.00 (reference) | Age, sex, BMI, alcohol intake, smoking, regular exercise, total calorie intake, dyslipidemia, hypertension, diabetes, CKD, and potassium intake |
|  |  |  |  |  |  |  |  |  |  |  |  |  |  |  |  |  |  | 1363.9-1989.0 mg/day | | | 0.79 (0.65, 0.98) |  |
|  |  |  |  |  |  |  |  |  |  |  |  |  |  |  |  |  |  | 1989.0-2602.2 mg/day | | | 1.01 (0.82, 1.24) |  |
|  |  |  |  |  |  |  |  |  |  |  |  |  |  |  |  |  |  | 2602.2-3422.7 mg/day | | | 1.03 (0.82, 1.30) |  |
|  |  |  |  |  |  |  |  |  |  |  |  |  |  |  |  |  |  | 3422.7-18190.2 mg/day | | | 0.92 (0.70, 1.21) |  |
| Mosallanezhad et al., 2023 | | the participants of the Tehran Lipid and Glucose Study | | Iran | | 10.6 yrs | | 2050 men and women | | 46.28 yrs | | Food frequency questionnaire | | Linking to the medical records and death certificates, or follow up via annually phone call | | CVD (208) | | 2.46 g day | | 1.00 (reference) | | CVD-risk score, physical activity, energy intakes (kcal/d), dietary intakes of total fats (g/d), fiber (g/d), eGFR and menopause status (yes/no). |
|  |  |  |  |  |  |  |  |  |  |  |  |  |  |  |  |  |  | 3.72 g day | | 0.92 (0.46, 1.85) | |  |
|  |  |  |  |  |  |  |  |  |  |  |  |  |  |  |  |  |  | 6.43 g/day | | 1.99 (1.06, 3.74) | |  |

***** Relative risks were employed as the common measure to quantify exposure-outcome association across studies, and hazard ratios were assumed equivalent to relative risks

† CVD included combined vascular events (CVD mortality, MI, stroke, and hospitalization for CHF).

Abbreviations: AF = atrial fibrillation; BMI = body mass index; CHD = coronary heart disease; CHF = congestive heart failure; CI = confidence interval; CRIC = Chronic Renal Insufficiency Cohort; CVD = cardiovascular disease; FFQ = food frequency questionnaire; HDL-C = high-density lipoprotein cholesterol; HF = heart failure; HPFS = Health Professionals’ Follow-up Study; JACC = Japan Collaborative Cohort; JDCS = Japan Diabetes Complications Study; LDL-C = low-density lipoprotein cholesterol; MI = myocardial infarction; MVP = Million Veteran Program; NHANES = National Health and Nutrition Examination Survey; NHS = Nurses’ Health Study; PREVEND = Prevention of Renal and Vascular End-stage Disease; PURE = Prospective Urban Rural Epidemiology; SBP = systolic blood pressure; TG = triglyceride; TOHP = Trials of Hypertension Prevention Collaborative Research Group

**Table S11.** Assessment of Risk of Study Bias Based on the Newcastle-Ottawa Scale

| Study | Selection | | | | Comparability | | Outcome | | | Total score |
| --- | --- | --- | --- | --- | --- | --- | --- | --- | --- | --- |
|  | a | b | c | d | e | f | g | h | i |  |
| Hu et al., 1992 | 1 | 1 | 0 | 1 | 0 | 0 | 1 | 0 | 1 | 5 |
| He et al., 1999 | 1 | 1 | 0 | 1 | 1 | 1 | 1 | 1 | 1 | 8 |
| Nagata et al., 2004 | 1 | 1 | 1 | 1 | 1 | 1 | 1 | 0 | 1 | 8 |
| Cohen et al., 2006 | 1 | 1 | 0 | 1 | 1 | 1 | 1 | 1 | 1 | 8 |
| Larsson et al., 2008 | 1 | 1 | 0 | 1 | 1 | 0 | 1 | 1 | 1 | 7 |
| Takachi et al., 2010 | 1 | 1 | 0 | 1 | 1 | 0 | 1 | 0 | 1 | 6 |
| O'Donnell et al., 2011 | 1 | 1 | 0 | 0 | 1 | 0 | 1 | 0 | 1 | 5 |
| Stolarz-Skrzypek et al., 2011 | 1 | 1 | 0 | 1 | 0 | 0 | 1 | 0 | 1 | 5 |
| Yang et al., 2011 | 1 | 1 | 0 | 1 | 0 | 0 | 1 | 1 | 1 | 6 |
| Gardener et al., 2012 | 1 | 1 | 0 | 1 | 1 | 1 | 1 | 1 | 1 | 8 |
| Ikehara et al., 2012 | 1 | 1 | 0 | 1 | 1 | 0 | 1 | 1 | 1 | 7 |
| Horikawa et al., 2014 | 1 | 1 | 1 | 1 | 1 | 1 | 1 | 0 | 1 | 8 |
| Joosten et al., 2014 | 1 | 1 | 1 | 1 | 0 | 0 | 1 | 1 | 1 | 7 |
| Pfister et al., 2014 | 1 | 1 | 1 | 1 | 1 | 0 | 1 | 1 | 1 | 8 |
| Kalogeropouloset al., 2015 | 1 | 1 | 0 | 1 | 0 | 0 | 1 | 1 | 1 | 6 |
| Singer et al., 2015 | 1 | 1 | 0 | 0 | 0 | 0 | 1 | 1 | 1 | 5 |
| Mills et al., 2016 | 1 | 1 | 1 | 0 | 1 | 0 | 1 | 0 | 1 | 6 |
| Olde-Engberink et al., 2017 | 1 | 1 | 1 | 0 | 0 | 0 | 1 | 1 | 1 | 6 |
| Kieneker et al., 2018 | 1 | 1 | 1 | 1 | 0 | 0 | 1 | 1 | 1 | 7 |
| Liu et al., 2018 | 1 | 1 | 1 | 1 | 1 | 0 | 1 | 1 | 1 | 8 |
| Kondo et al., 2019 | 1 | 1 | 0 | 1 | 0 | 0 | 1 | 1 | 1 | 6 |
| O'Donnell et al., 2019 | 1 | 1 | 0 | 0 | 1 | 0 | 1 | 0 | 1 | 5 |
| Sadanaga et al., 2019 | 1 | 1 | 1 | 0 | 0 | 0 | 1 | 0 | 1 | 5 |
| Elliott et al., 2020 | 1 | 1 | 0 | 1 | 1 | 0 | 1 | 0 | 1 | 6 |
| Vuoro et al., 2020 | 1 | 1 | 0 | 1 | 0 | 0 | 1 | 1 | 1 | 6 |
| Nohara-Shitama et al., 2021 | 1 | 1 | 0 | 1 | 0 | 0 | 1 | 1 | 1 | 6 |
| Pickering et al., 2021 | 1 | 1 | 1 | 1 | 0 | 0 | 1 | 1 | 1 | 7 |
| Wang et al., 2021 | 1 | 1 | 0 | 1 | 1 | 0 | 1 | 1 | 1 | 7 |
| Wuopio et al., 2021 | 1 | 1 | 0 | 1 | 0 | 0 | 1 | 0 | 1 | 5 |
| Ma et al., 2022 | 1 | 1 | 0 | 1 | 1 | 0 | 1 | 0 | 1 | 6 |
| Wang et al., 2022 | 1 | 1 | 0 | 1 | 1 | 0 | 1 | 0 | 1 | 6 |
| Kwon et al., 2022 | 1 | 1 | 0 | 0 | 1 | 1 | 1 | 1 | 1 | 8 |
| Mosallanezhad et al., 2023 | 1 | 1 | 0 | 1 | 0 | 1 | 1 | 1 | 1 | 8 |
| Current, 2022 | 1 | 1 | 0 | 1 | 1 | 0 | 1 | 1 | 1 | 7 |

a. Representativeness of the exposed cohort: 1 point awarded if community-based population;

b. Selection of the non-exposed cohort: 1 point awarded if drawn from the same community as the exposed cohort;

c. Ascertainment of exposure: 1 point awarded if diet assessed at baseline and at least one time during follow-up period;

d. Outcome of interest not present at start of the study: 1 point awarded if individuals with prevalent CVD at baseline were excluded;

e. Control for primary confounders: 1 point awarded if models were adjusted for age, sex, BMI, smoking status, alcohol intake, and physical activity;

f. Control for secondary confounders: 1 point awarded if models were adjusted for blood pressure-lowering drugs/hypertension status, history of diabetes, and total energy intake;

g. Assessment of outcome: 1 point awarded if non-fatal cases were confirmed by physician’s diagnosis, and fatal cases confirmed by death certificates;

h. Duration of follow-up: 1 point awarded if follow-up ≥ 10 years;

i. Adequacy of follow-up: 1 point awarded if loss to follow-up <20% or lost subjects unlikely to introduce bias.

**Table S12.** Prespecified Subgroup Meta-Analysis Using Random-Effects Models for the Association Between Sodium Intake and Risk of Cardiovascular Disease, with the Comparison of Highest Versus Lowest Categories of Sodium Intake.

| Stratification and categories | No. of risk estimate | No. of participants | No. of events | Pooled RR (95% CI) | I^2^ (%) | *P* for interaction |
| --- | --- | --- | --- | --- | --- | --- |
| Sex |  |  |  |  |  |  |
| Both | 26 | 842,034 | 37,597 | 1.19 (1.09, 1.31) | 79.0 | 0.22 |
| Men | 9 | 723,969 | 29,194 | 1.01 (0.93, 1.10) | 52.7 |  |
| Women | 7 | 519,901 | 13,294 | 1.07 (0.98, 1.18) | 24.8 |  |
| Number of participants |  |  |  |  |  |  |
| <10000 | 25 | 101,961 | 12,108 | 1.14 (1.03, 1.34) | 79.0 | 0.59 |
| ≥10000 | 17 | 1,983,943 | 67,977 | 1.08 (1.03, 1.14) | 53.3 |  |
| Duration years of follow-up |  |  |  |  |  |  |
| <10 years | 15 | 1,320,537 | 38,409 | 1.20 (1.09, 1.33) | 83.2 | 0.14 |
| ≥10 years | 27 | 765,367 | 41,676 | 1.08 (1.00, 1.17) | 60.9 |  |
| Number of events |  |  |  |  |  |  |
| <1000 | 27 | 277,039 | 10,174 | 1.21 (1.04, 1.41) | 79.3 | 0.26 |
| ≥1000 | 15 | 1,808,865 | 69,911 | 1.08 (1.03, 1.12) | 37.2 |  |
| Geographical location |  |  |  |  |  |  |
| US | 15 | 650,137 | 34,351 | 1.10 (1.00, 1.20) | 59.7 | 0.49 |
| Non-US | 25 | 1,157,217 | 31,928 | 1.16 (1.06, 1.27) | 77.7 |  |
| Risk of study bias |  |  |  |  |  |  |
| Low (≥7) | 21 | 762,497 | 38,239 | 1.12 (1.03, 1.22) | 63.5 | 0.91 |
| Moderate (≤6) | 21 | 1,323,407 | 41,846 | 1.13 (1.03, 1.24) | 79.2 |  |
| Times of exposure assessment |  |  |  |  |  |  |
| Baseline only | 30 | 2,012,514 | 75,805 | 1.08 (1.02, 1.14) | 64.5 | 0.027 |
| Repeated | 12 | 73,390 | 4,280 | 1.36 (1.09, 1.69) | 79.1 |  |
| Approach of exposure assessment |  |  |  |  |  |  |
| Dietary questionnaire | 23 | 1,014,282 | 44,530 | 1.11 (1.04, 1.20) | 57.7 | 0.63 |
| Spot urine | 8 | 1,025,536 | 31,554 | 1.09 (0.97, 1.23) | 85.6 |  |
| Timed urine | 11 | 46,086 | 4,001 | 1.17 (0.93, 1.47) | 79.5 |  |
| Publication year |  |  |  |  |  |  |
| Before 2014 | 19 | 319,425 | 21,799 | 1.12 (1.02, 1.22) | 67.5 | 0.97 |
| After 2014 | 23 | 1,766,479 | 58,286 | 1.13 (1.04, 1.24) | 77.0 |  |
| Times and approach of exposure assessment |  |  |  |  |  |  |
| Repeated measurements and 24-hour urine sodium | 5 | 20,158 | 1,957 | 1.30 (0.91, 1.87) | 72.3 | 0.32 |
| Other measurements | 37 | 2,065,746 | 78,128 | 1.11 (1.05, 1.18) | 75.6 |  |

I^2^ refers to proportion of heterogeneity among studies.

Abbreviations: CI = confidence interval; RR = relative risk

**Fig. S1** Sex Stratified Associations Between Sodium Intake and Overall and Cause-specific Mortality in Minimally Adjusted Cubic Spline Regression Models.

**
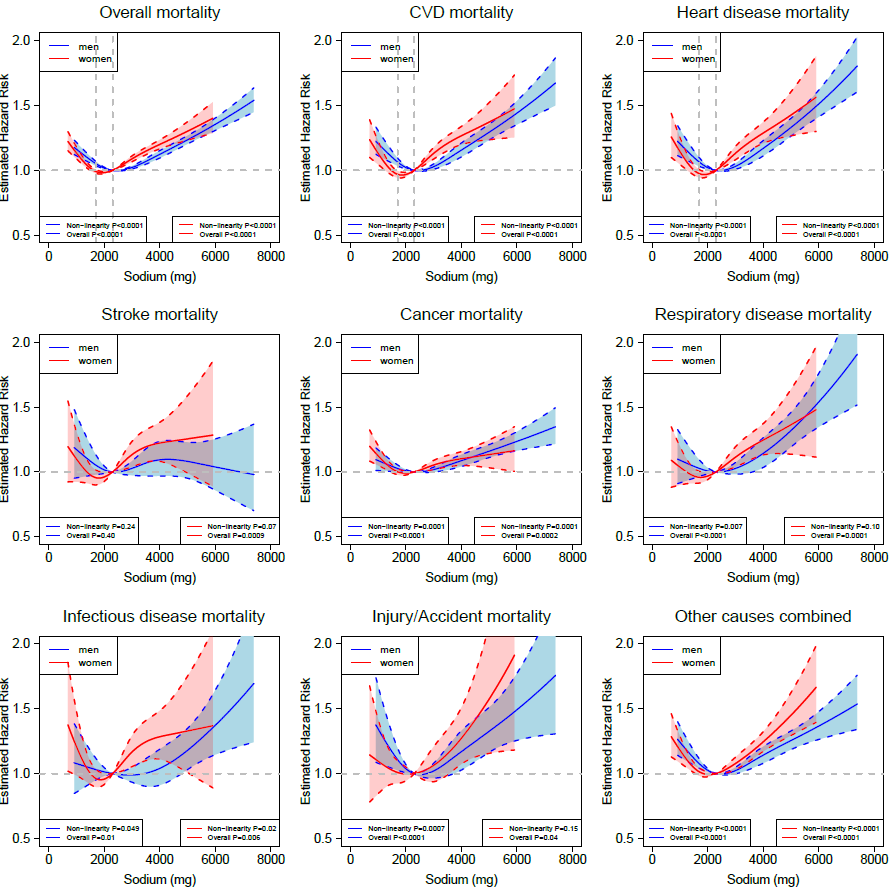
**

Analyses were adjusted for age at baseline and race or ethnic group. The solid line denotes the HR of overall mortality according to sodium intake with a four-knot cubic spline selected at the 5th, 25th, 75th, and 95th percentiles of intake, dashed lines and shaded areas represent the 95% confidence intervals, blue indicates men and red indicates women.

**Fig. S2** Sex Stratified Associations Between Potassium Intake and Overall and Cause-specific Mortality in Minimally Adjusted Cubic Spline Regression Models.

**
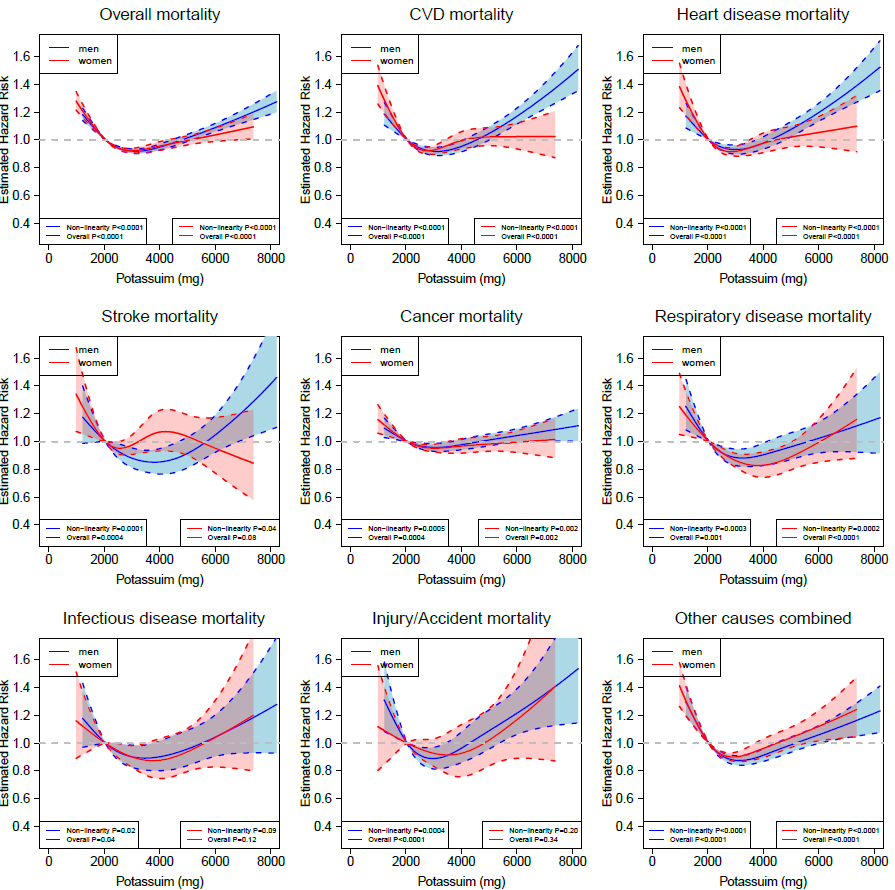
**

Analyses were adjusted for age at baseline and race or ethnic group. The solid line denotes the HR of overall mortality according to potassium intake with a four-knot cubic spline selected at the 5th, 25th, 75th, and 95th percentiles of intake, dashed lines and shaded areas represent the 95% confidence intervals, blue indicates men and red indicates women.

**Fig. S3** Sex Stratified Associations Between Intake of Sodium-Potassium Ratio and Overall and Cause-specific Mortality in Minimally Adjusted Cubic Spline Regression Models.

**
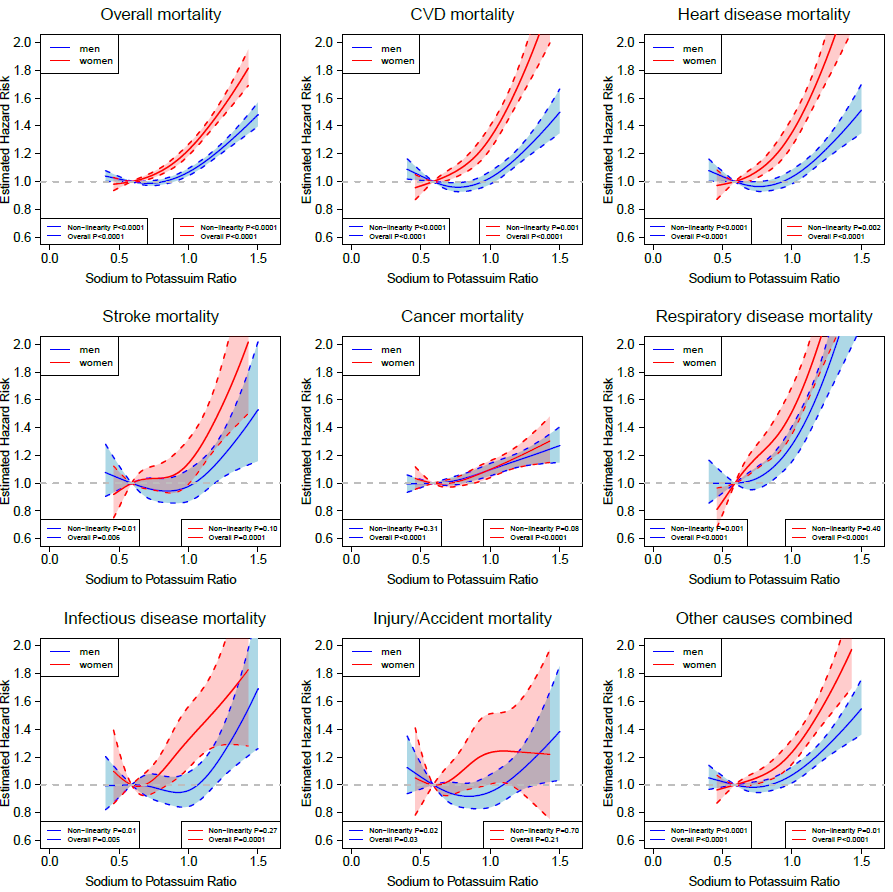
**

Analyses were adjusted for age at baseline and race or ethnic group. The solid line denotes the HR of overall mortality according to sodium-potassium ratio with a four-knot cubic spline selected at the 5th, 25th, 75th, and 95th percentiles of intake, dashed lines and shaded areas represent the 95% confidence intervals, blue indicates men and red indicates women.

**Fig. S4** Sex Stratified Associations Between Sodium Intake and Overall and Cause-specific Mortality in Parsimoniously Adjusted Cubic Spline Regression Models.


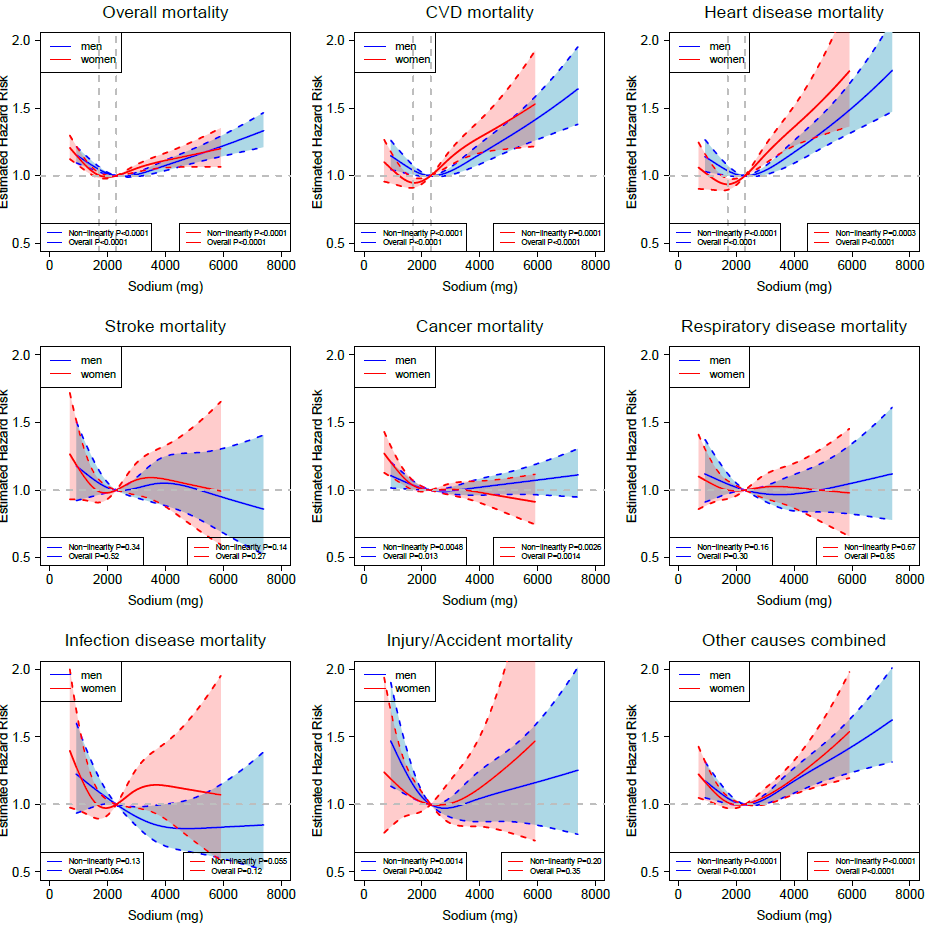


Analyses were parsimoniously adjusted for age at baseline, BMI, alcohol consumption, smoking status, physical activity, race or ethnic group, education, health status, and total energy intake. The solid line denotes the HR of overall mortality according to sodium intake with a four-knot cubic spline selected at the 5th, 25th, 75th, and 95th percentiles of intake, dashed lines and shaded areas represent the 95% confidence intervals, blue indicates men and red indicates women.

**Fig. S5** Sex Stratified Associations Between Potassium Intake and Overall and Cause-specific Mortality in Parsimoniously Adjusted Cubic Spline Regression Models.


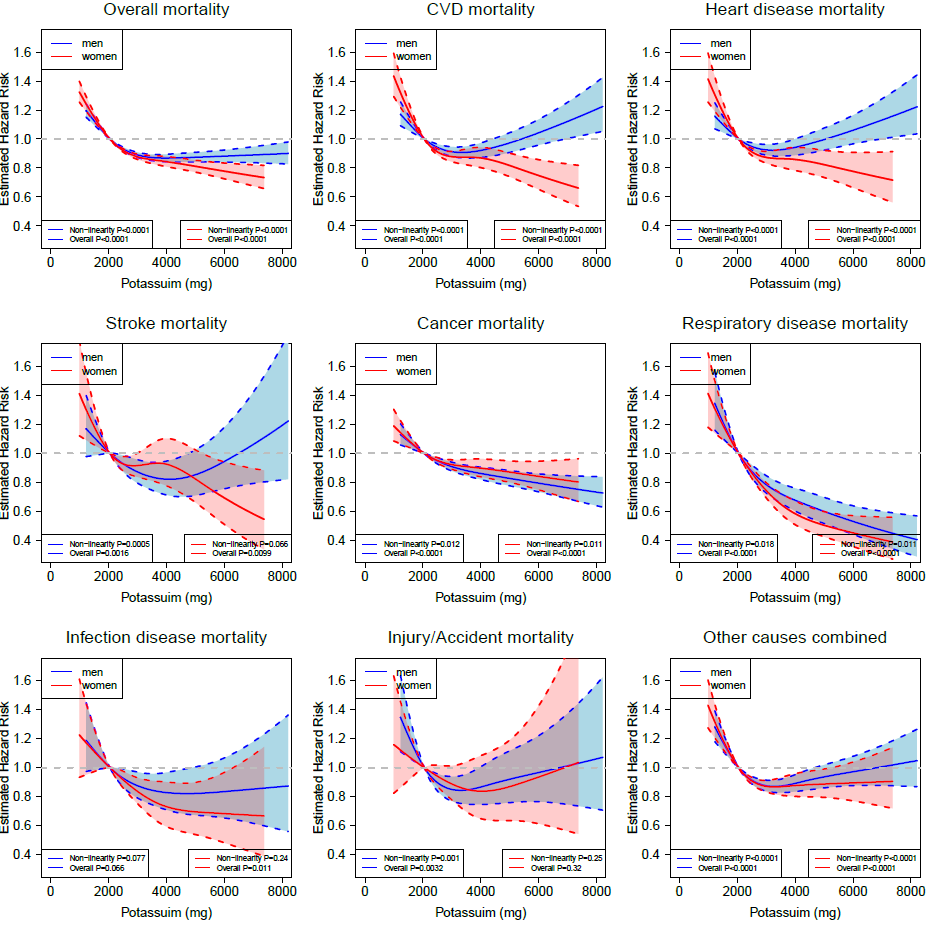


Analyses were parsimoniously adjusted for age at baseline, BMI, alcohol consumption, smoking status, physical activity, race or ethnic group, education, health status, and total energy intake. The solid line denotes the HR of overall mortality according to sodium intake with a four-knot cubic spline selected at the 5th, 25th, 75th, and 95th percentiles of intake, dashed lines and shaded areas represent the 95% confidence intervals, blue indicates men and red indicates women.

**Fig. S6** Sex Stratified Associations Between Intake of Sodium-Potassium Ratio and Overall and Cause-specific Mortality in Parsimoniously Adjusted Cubic Spline Regression Models.


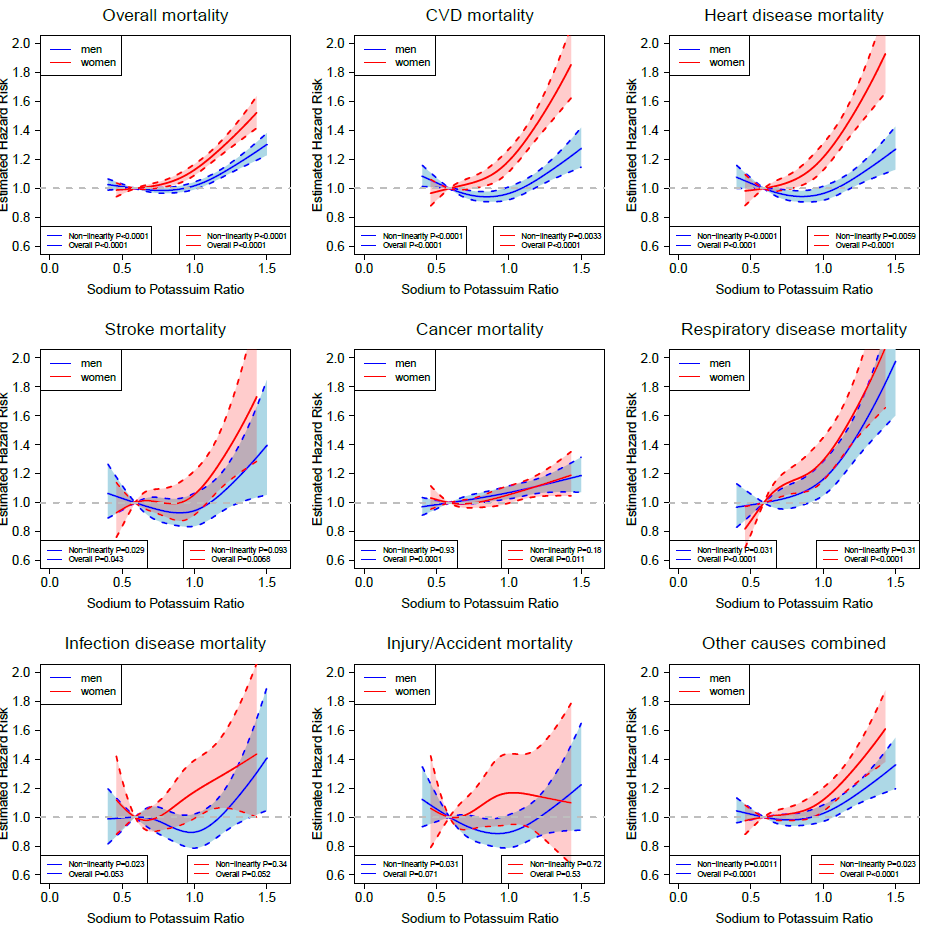


Analyses were parsimoniously adjusted for age at baseline, BMI, alcohol consumption, smoking status, physical activity, race or ethnic group, education, health status, and total energy intake. The solid line denotes the HR of overall mortality according to sodium intake with a four-knot cubic spline selected at the 5th, 25th, 75th, and 95th percentiles of intake, dashed lines and shaded areas represent the 95% confidence intervals, blue indicates men and red indicates women.

**Fig. S7** Sex Stratified Associations Between Sodium Intake and Overall and Cause-specific Mortality in Multivariable-Adjusted Cubic Spline Regression Models, Excluding the Initial 5 Years of Follow-up.

**
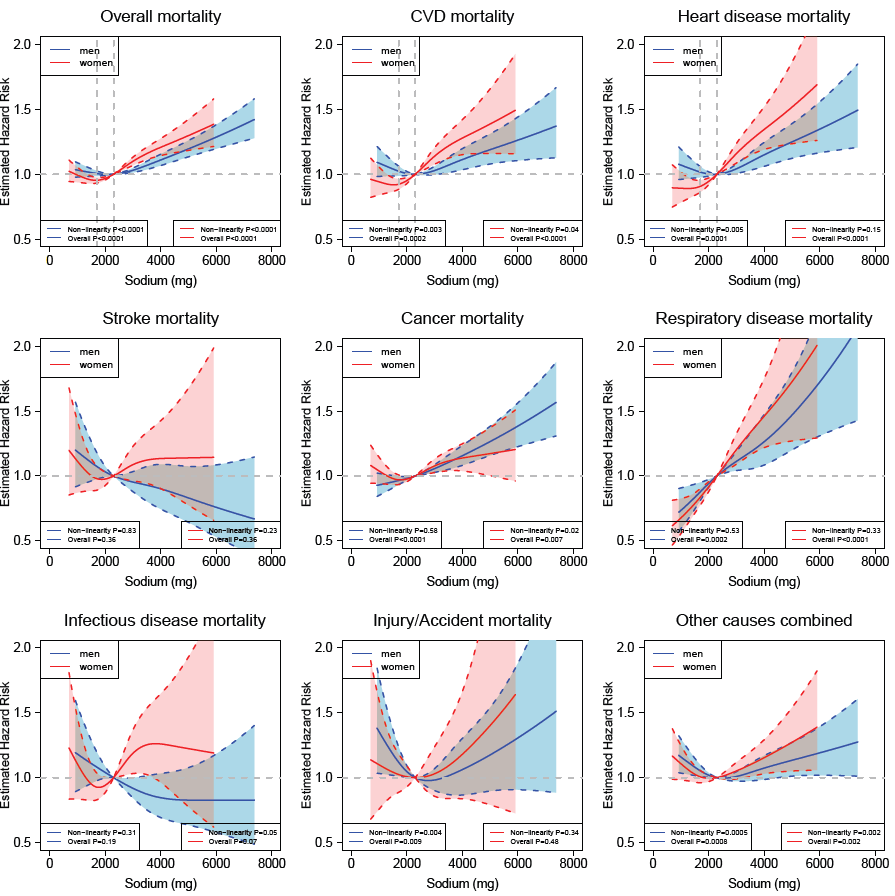
**

Analyses were adjusted for age at baseline, BMI, alcohol consumption, smoking status (never, former, current or missing), physical activity, race or ethnic group, education, marital status, diabetes (yes vs. no), health status, vitamin supplement use, total energy intake, and the Healthy Eating Index 2015 (HEI-2015) score excluding the sodium component. For women, the risk estimates were additionally adjusted for postmenopausal hormone therapy (yes vs. no). The solid line denotes the HR of overall mortality according to dietary sodium intake with a four-knot cubic spline selected at the 5th, 25th, 75th, and 95th percentiles of intake, dashed lines and shaded areas represent the 95% confidence intervals, blue indicates men and red indicates women.

**Fig. S8** Sex Stratified Associations Between Potassium Intake and Overall and Cause-specific Mortality in Multivariable-Adjusted Cubic Spline Regression Models, Excluding the Initial 5 Years of Follow-up.

**
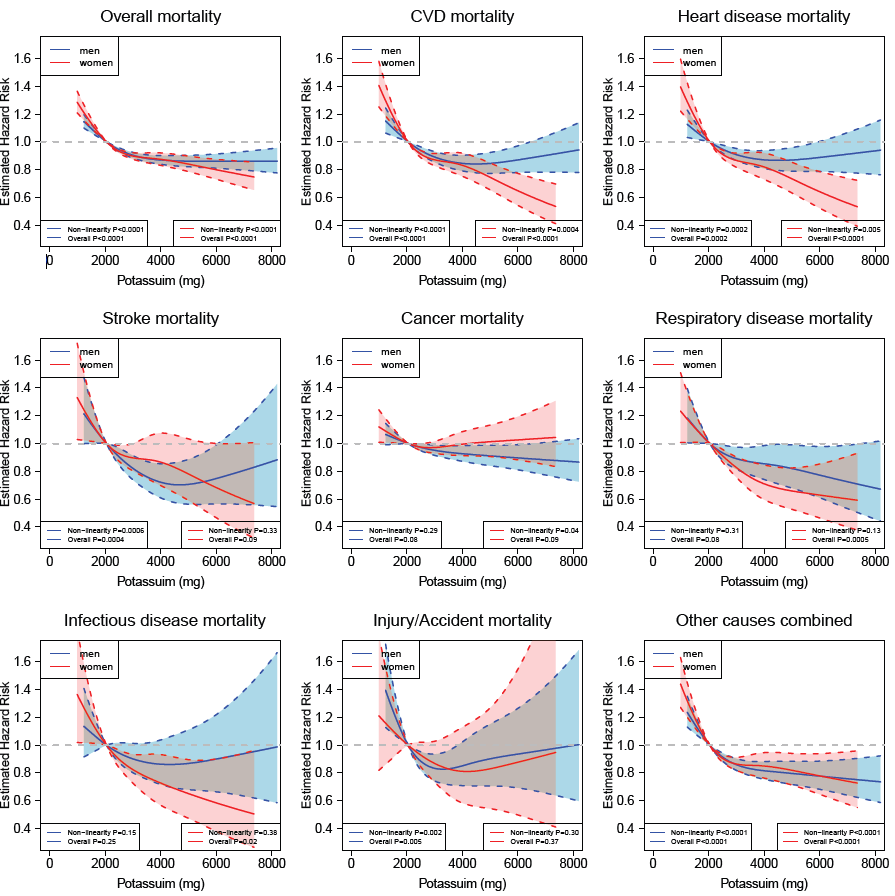
**

Analyses were adjusted for age at baseline, BMI, alcohol consumption, smoking status (never, former, current or missing), physical activity, race or ethnic group, education, marital status, diabetes (yes vs. no), health status, vitamin supplement use, total energy intake, and the Healthy Eating Index 2015 (HEI-2015) score components of sodium, seafood and plant protein, saturated fat, fatty acids and refined grains. For women, the risk estimates were additionally adjusted for postmenopausal hormone therapy (yes vs. no). The solid line denotes the HR of overall mortality according to dietary potassium intake with a four-knot cubic spline selected at the 5th, 25th, 75th, and 95th percentiles of intake, dashed lines and shaded areas represent the 95% confidence intervals, blue indicates men and red indicates women.

**Fig. S9** Sex Stratified Associations Between Sodium-Potassium Ratio and Overall and Cause-specific Mortality in Multivariable-Adjusted Cubic Spline Regression Models, Excluding the Initial 5 Years of Follow-up.

**
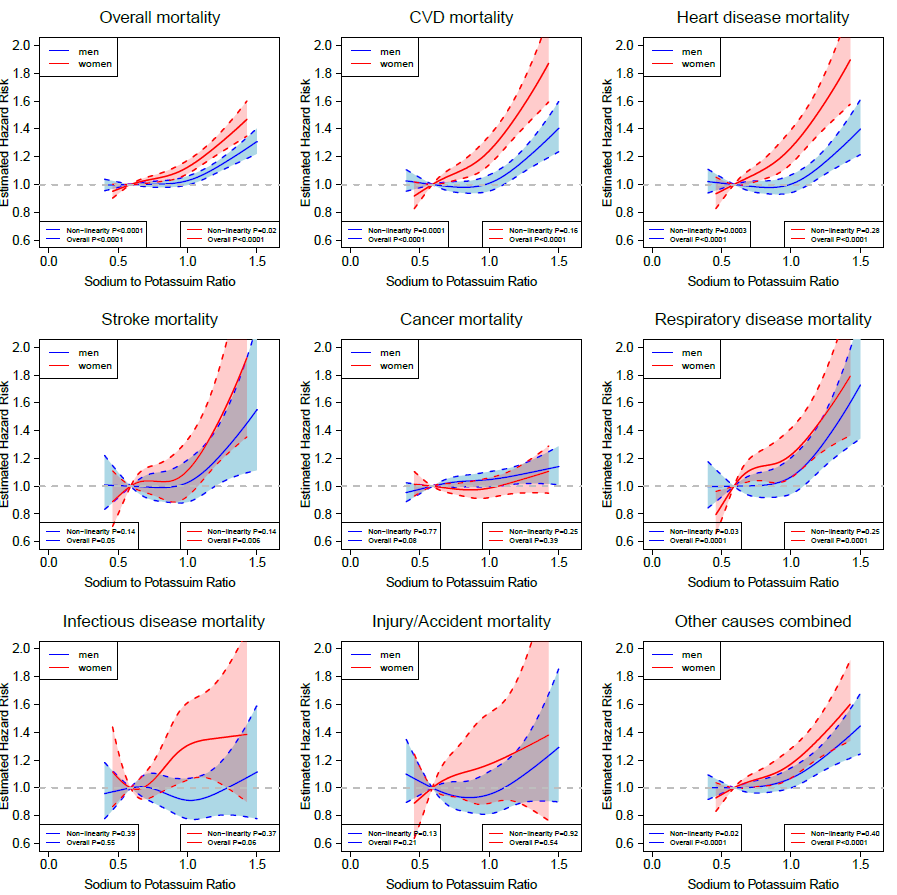
**

Analyses were adjusted for age at baseline, BMI, alcohol consumption, smoking status (never, former, current or missing), physical activity, race or ethnic group, education, marital status, diabetes (yes vs. no), health status, vitamin supplement use, total energy intake, and the Healthy Eating Index 2015 (HEI-2015) score components of seafood and plant protein, saturated fat, fatty acids and refined grains. For women, the risk estimates were additionally adjusted for postmenopausal hormone therapy (yes vs. no). The solid line denotes the HR of overall mortality according to sodium-potassium ratio with a four-knot cubic spline selected at the 5th, 25th, 75th, and 95th percentiles of intake, dashed lines and shaded areas represent the 95% confidence intervals, blue indicates men and red indicates women.

**Fig. S10** Sex Stratified Associations Between Sodium Intake and Overall and Cause-specific Mortality in Multivariable-Adjusted Cubic Spline Regression Models, Excluding the Participants Reporting a History of Diabetes at Baseline.

**
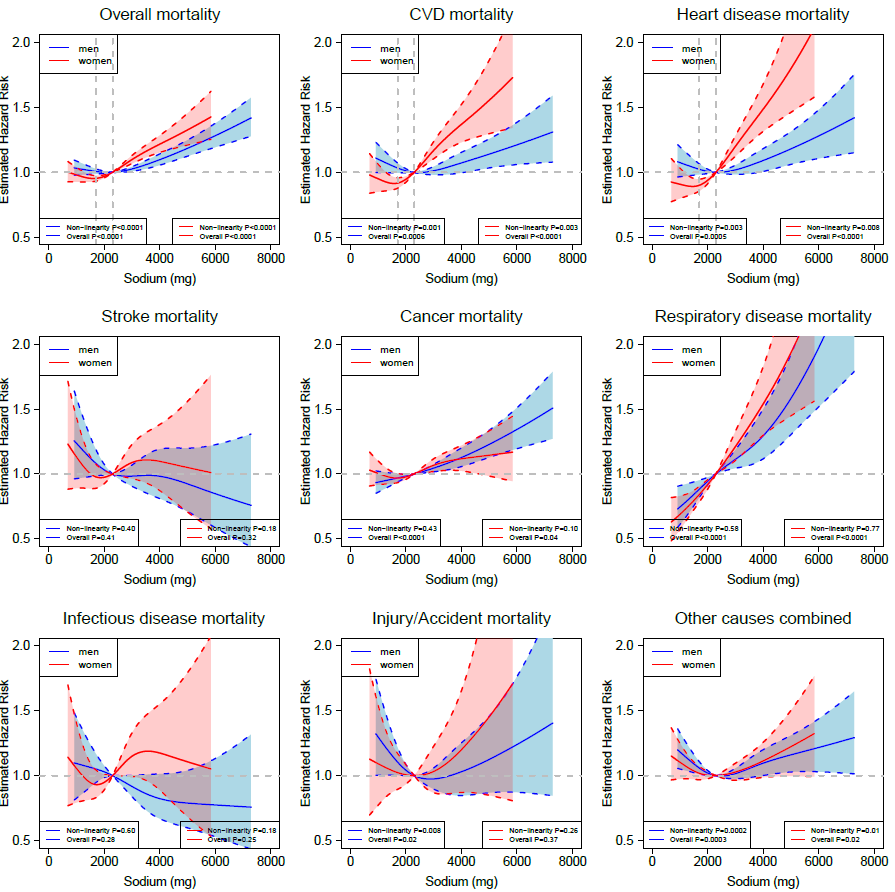
**

Analyses were adjusted for age at baseline, BMI, alcohol consumption, smoking status (never, former, current or missing), physical activity, race or ethnic group, education, marital status, health status, vitamin supplement use, total energy intake, and the Healthy Eating Index 2015 (HEI-2015) score excluding the sodium component. For women, the risk estimates were additionally adjusted for postmenopausal hormone therapy (yes vs. no). The solid line denotes the HR of overall mortality according to dietary sodium intake with a four-knot cubic spline selected at the 5th, 25th, 75th, and 95th percentiles of intake, dashed lines and shaded areas represent the 95% confidence intervals, blue indicates men and red indicates women.

**Fig. S11** Sex Stratified Associations Between Potassium Intake and Overall and Cause-specific Mortality in Multivariable-Adjusted Cubic Spline Regression Models, Excluding the Participants Reporting a History of Diabetes at Baseline.

**
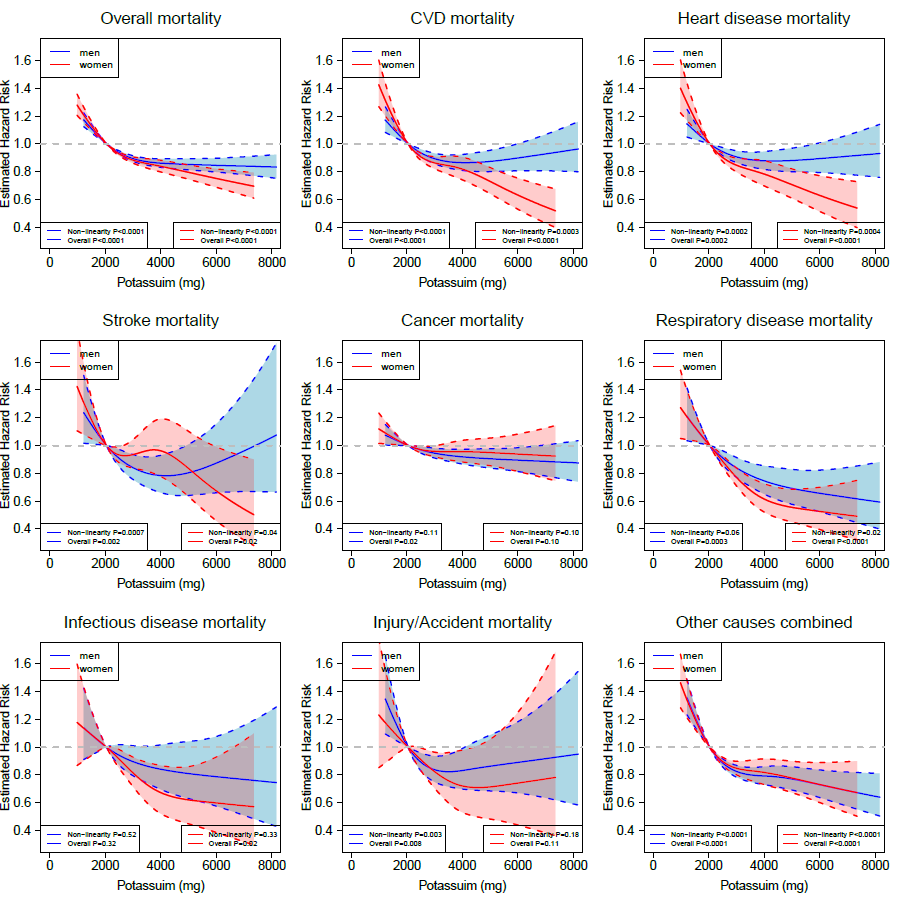
**

Analyses were adjusted for age at baseline, BMI, alcohol consumption, smoking status (never, former, current or missing), physical activity, race or ethnic group, education, marital status, health status, vitamin supplement use, total energy intake, and the Healthy Eating Index 2015 (HEI-2015) score components of sodium, seafood and plant protein, saturated fat, fatty acids and refined grains. For women, the risk estimates were additionally adjusted for postmenopausal hormone therapy (yes vs. no). The solid line denotes the HR of overall mortality according to dietary potassium intake with a four-knot cubic spline selected at the 5th, 25th, 75th, and 95th percentiles of intake, dashed lines and shaded areas represent the 95% confidence intervals, blue indicates men and red indicates women.

**Fig. S12** Sex Stratified Associations Between Sodium-Potassium Ratio and Overall and Cause-specific Mortality in Multivariable-Adjusted Cubic Spline Regression Models, Excluding the Participants Reporting a History of Diabetes at Baseline.

**
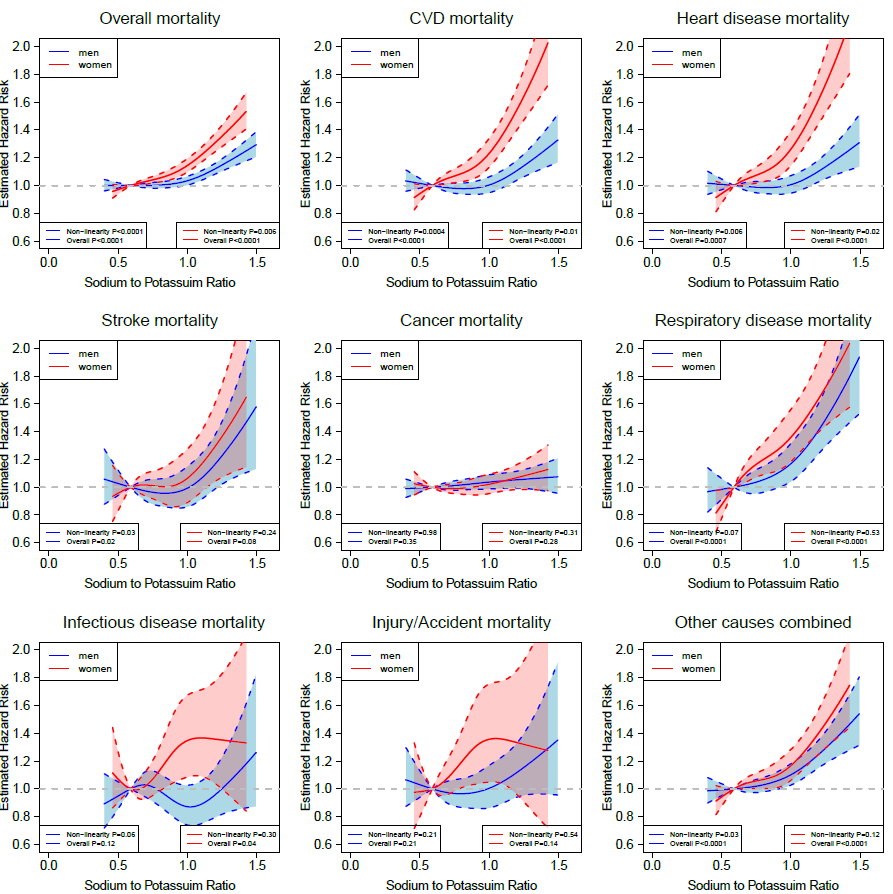
**

Analyses were adjusted for age at baseline, BMI, alcohol consumption, smoking status (never, former, current or missing), physical activity, race or ethnic group, education, marital status, health status, vitamin supplement use, total energy intake, and the Healthy Eating Index 2015 (HEI-2015) score components of seafood and plant protein, saturated fat, fatty acids and refined grains. For women, the risk estimates were additionally adjusted for postmenopausal hormone therapy (yes vs. no). The solid line denotes the HR of overall mortality according to sodium-potassium ratio with a four-knot cubic spline selected at the 5th, 25th, 75th, and 95th percentiles, dashed lines and shaded areas represent the 95% confidence intervals, blue indicates men and red indicates women.

**Fig. S13** Sex-Stratified Associations Between Sodium Intake and Overall and Cause-specific Mortality in Multivariable-Adjusted Cubic Spline Regression Models, Excluding Participants Reporting Poor to Fair Health Status or Unknown Health Status.


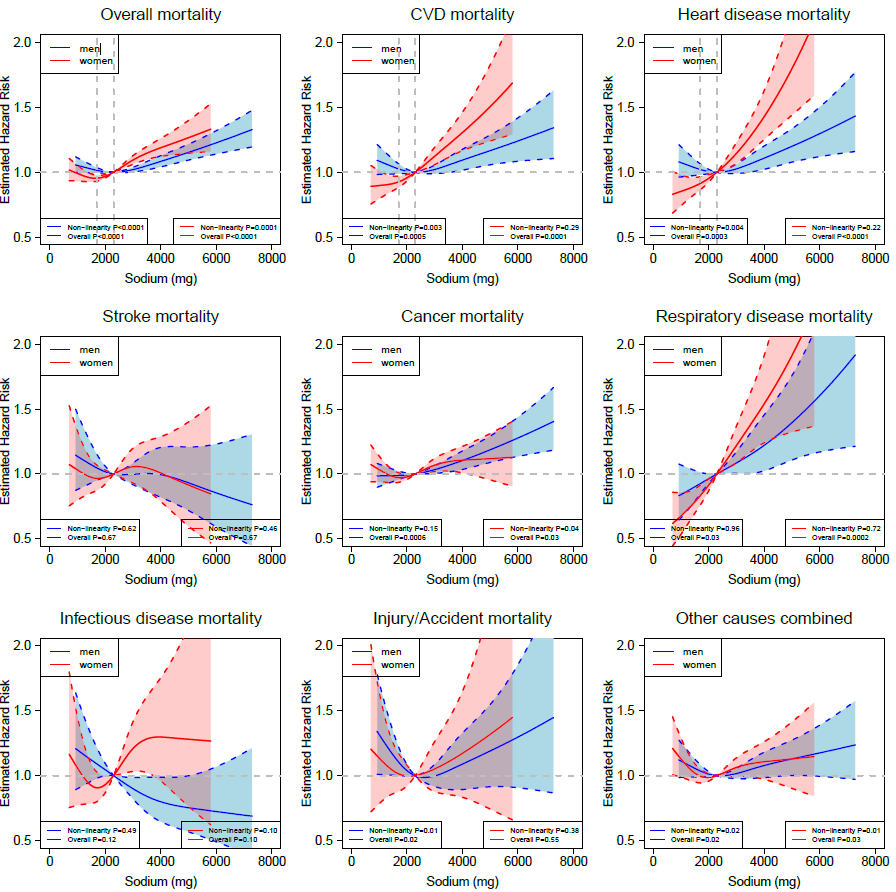


Analyses were adjusted for age, BMI, alcohol consumption, smoking status (never, former, current or missing), physical activity, race or ethnic group, education, marital status, diabetes, health status, vitamin supplement use, total energy intake, and the Healthy Eating Index 2015 (HEI-2015) score components of seafood and plant protein, saturated fat, fatty acids and refined grains. For women, the risk estimates were additionally adjusted for postmenopausal hormone therapy. The solid line denotes the HR of overall mortality according to dietary sodium intake with a four-knot cubic spline selected at the 5th, 25th, 75th, and 95th percentiles of intake, dashed lines and shaded areas represent the 95% confidence intervals, blue indicates men and red indicates women.

**Fig. S14** Sex-Stratified Associations Between Potassium Intake and Overall and Cause-specific Mortality in Multivariable-Adjusted Cubic Spline Regression Models, Excluding Participants Reporting Poor to Fair Health Status or Unknown Health Status.


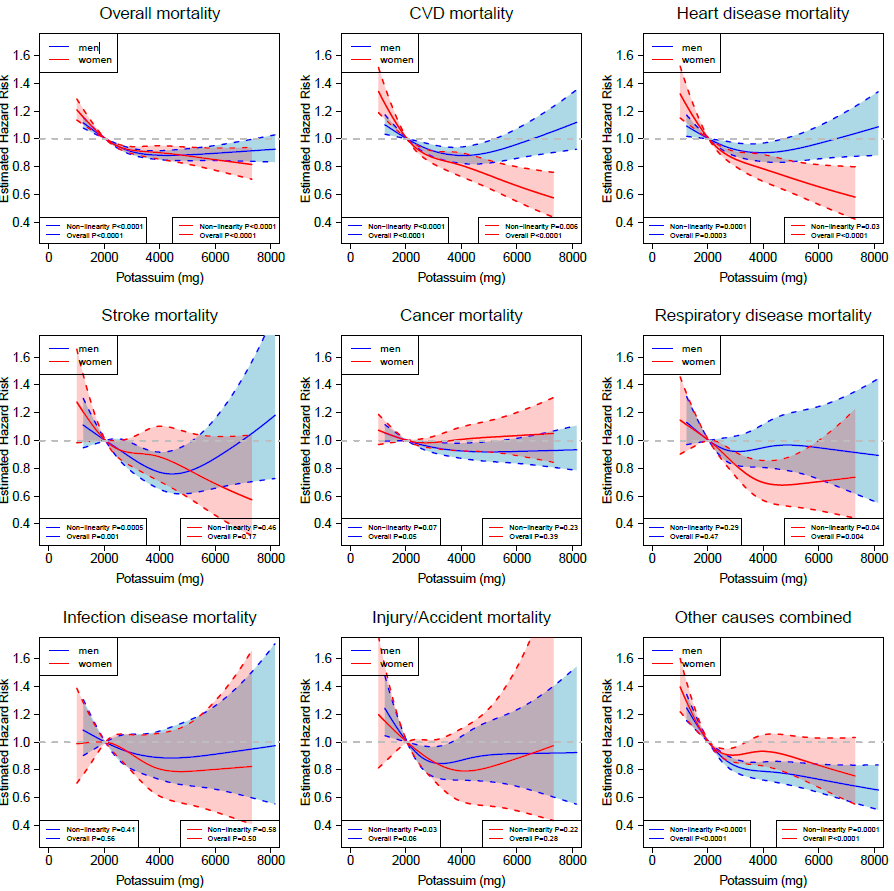


Analyses were adjusted for age at baseline, BMI, alcohol consumption, smoking status (never, former, current or missing), physical activity, race or ethnic group, education, marital status, diabetes (yes vs. no), health status, vitamin supplement use, total energy intake, and the Healthy Eating Index 2015 (HEI-2015) score components of sodium, seafood and plant protein, saturated fat, fatty acids and refined grains. For women, the risk estimates were additionally adjusted for postmenopausal hormone therapy (yes vs. no). The solid line denotes the HR of overall mortality according to dietary potassium intake with a four-knot cubic spline selected at the 5th, 25th, 75th, and 95th percentiles of intake, dashed lines and shaded areas represent the 95% confidence intervals, blue indicates men and red indicates women.

**Fig. S15** Sex-Stratified Associations Between Sodium-Potassium Ratio and Overall and Cause-specific Mortality in Multivariable-Adjusted Cubic Spline Regression Models, Excluding Participants Reporting Poor to Fair Health Status or Unknown Health Status.


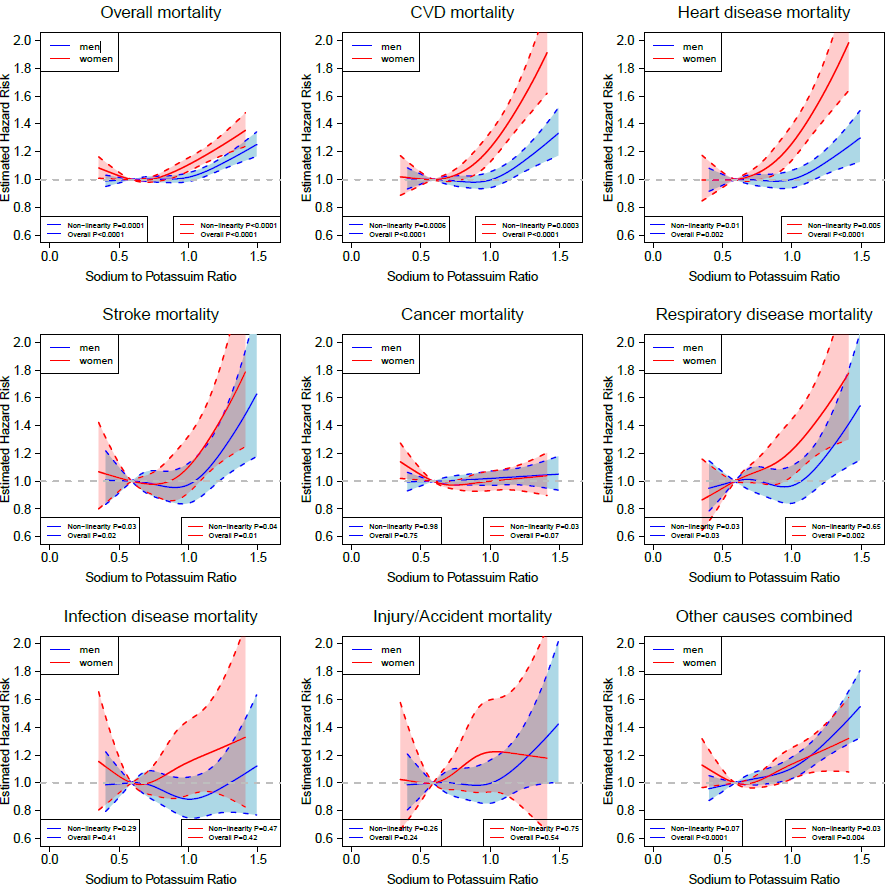


Analyses were adjusted for age at baseline, BMI, alcohol consumption, smoking status (never, former, current or missing), physical activity, race or ethnic group, education, marital status, diabetes (yes vs. no), health status, vitamin supplement use, total energy intake, and the Healthy Eating Index 2015 (HEI-2015) score components of seafood and plant protein, saturated fat, fatty acids and refined grains. For women, the risk estimates were additionally adjusted for postmenopausal hormone therapy (yes vs. no). The solid line denotes the HR of overall mortality according to sodium-potassium ratio with a four-knot cubic spline selected at the 5th, 25th, 75th, and 95th percentiles of intake, dashed lines and shaded areas represent the 95% confidence intervals, blue indicates men and red indicates women.

**Fig. S16** Sex-Stratified Associations Between Sodium Intake and Overall and Cause-specific Mortality in Multivariable-Adjusted Cubic Spline Regression Models, **Additionally Adjusted for Family Income.**


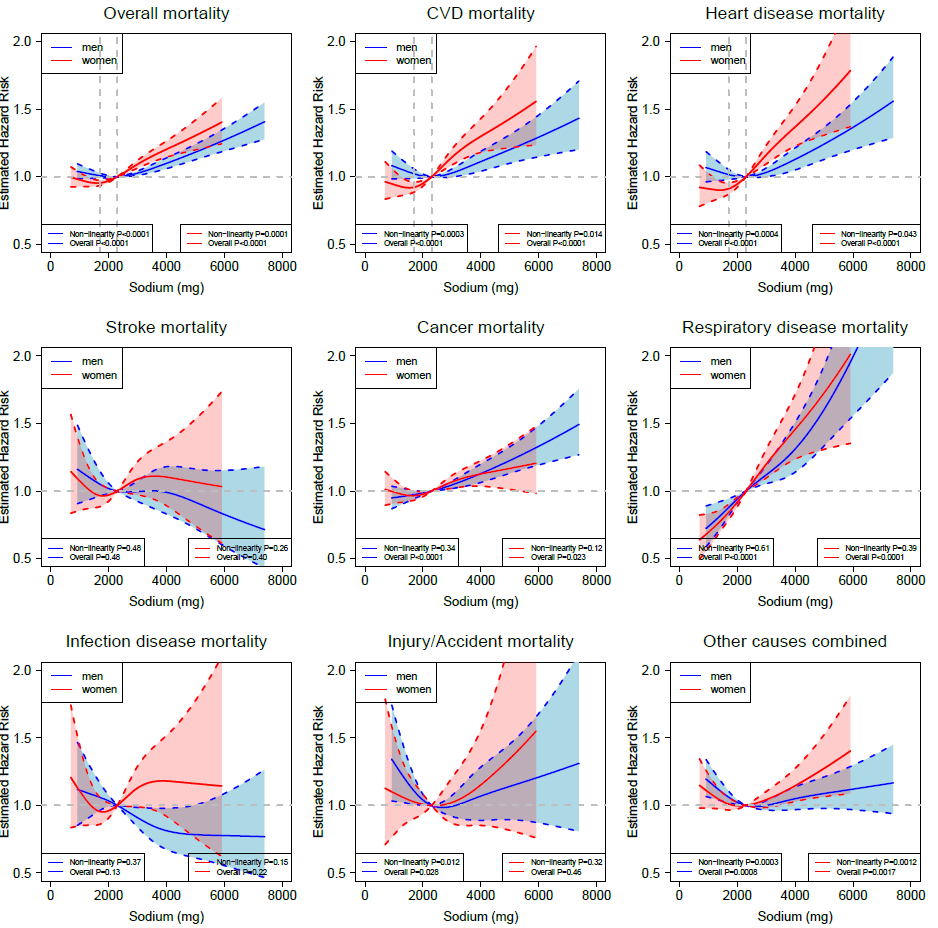


Analyses were adjusted for age at baseline, BMI, alcohol consumption, smoking status (never, former, current or missing), physical activity, race or ethnic group, education, marital status, diabetes (yes vs. no), health status, vitamin supplement use, family income, total energy intake, and the Healthy Eating Index 2015 (HEI-2015) score excluding the sodium component. For women, the risk estimates were additionally adjusted for postmenopausal hormone therapy (yes vs. no). The solid line denotes the HR of overall mortality according to dietary sodium intake with a four-knot cubic spline selected at the 5th, 25th, 75th, and 95th percentiles of intake, dashed lines and shaded areas represent the 95% confidence intervals, blue indicates men and red indicates women.

**Fig. S17** Sex Stratified Associations Between Potassium Intake and Overall and Cause-specific Mortality in Multivariable-Adjusted Cubic Spline Regression Models, **Additionally Adjusted for Family Income.**


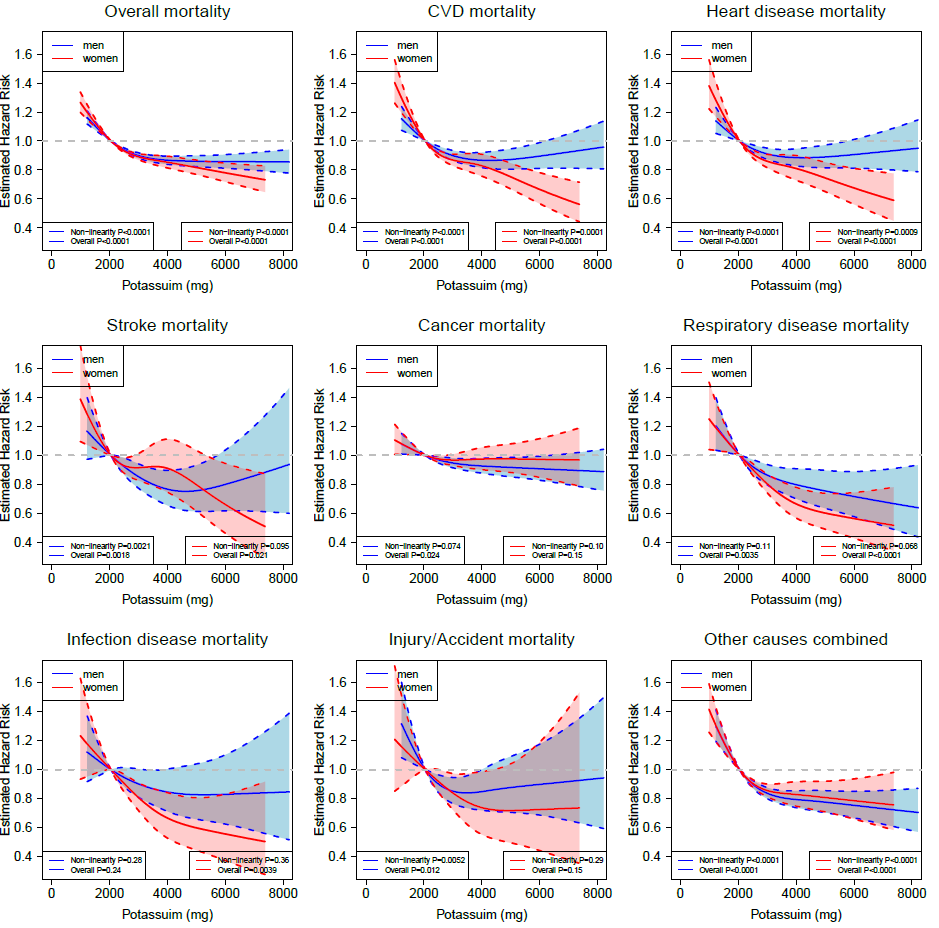


Analyses were adjusted for age at baseline, BMI, alcohol consumption, smoking status (never, former, current or missing), physical activity, race or ethnic group, education, marital status, diabetes (yes vs. no), health status, vitamin supplement use, family income, total energy intake, and the Healthy Eating Index 2015 (HEI-2015) score components of sodium, seafood and plant protein, saturated fat, fatty acids and refined grains. For women, the risk estimates were additionally adjusted for postmenopausal hormone therapy (yes vs. no). The solid line denotes the HR of overall mortality according to dietary potassium intake with a four-knot cubic spline selected at the 5th, 25th, 75th, and 95th percentiles of intake, dashed lines and shaded areas represent the 95% confidence intervals, blue indicates men and red indicates women.

**Fig. S18** Sex Stratified Associations Between Sodium-Potassium Ratio and Overall and Cause-specific Mortality in Multivariable-Adjusted Cubic Spline Regression Models, **Additionally Adjusted for Family Income.**


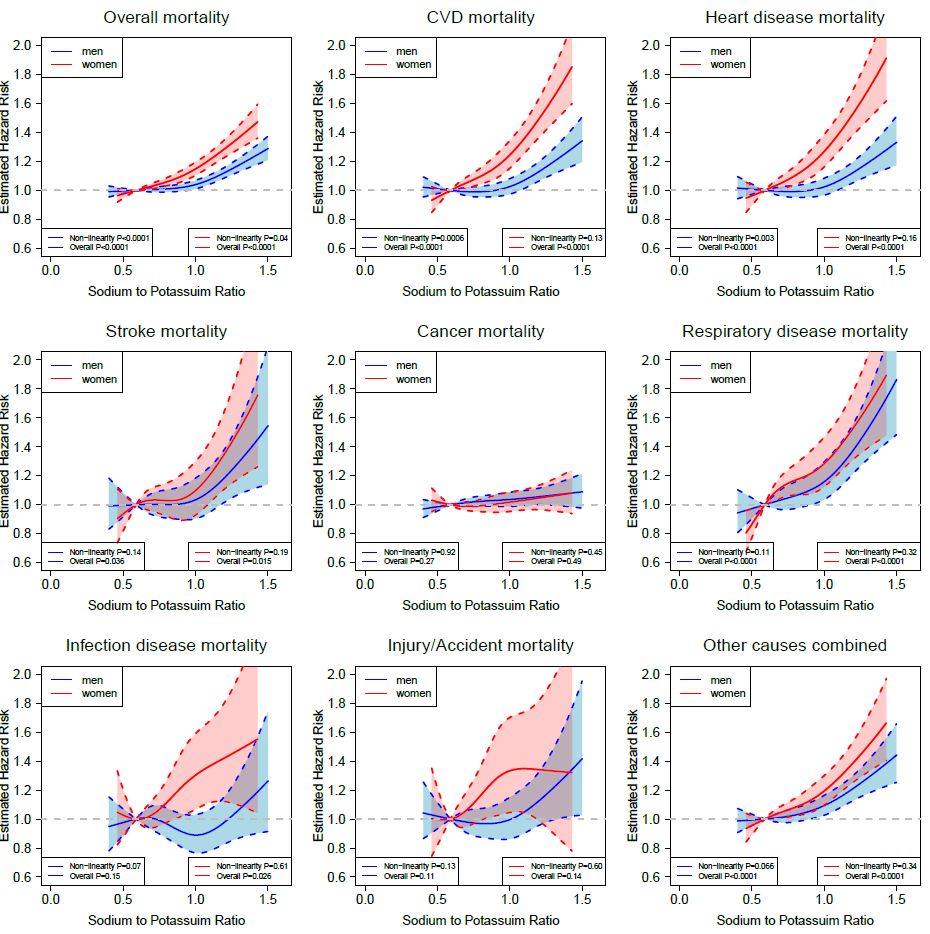


Analyses were adjusted for age at baseline, BMI, alcohol consumption, smoking status (never, former, current or missing), physical activity, race or ethnic group, education, marital status, diabetes (yes vs. no), health status, vitamin supplement use, family income, total energy intake, and the Healthy Eating Index 2015 (HEI-2015) score components of seafood and plant protein, saturated fat, fatty acids and refined grains. For women, the risk estimates were additionally adjusted for postmenopausal hormone therapy (yes vs. no). The solid line denotes the HR of overall mortality according to sodium-potassium ratio with a four-knot cubic spline selected at the 5th, 25th, 75th, and 95th percentiles of intake, dashed lines and shaded areas represent the 95% confidence intervals, blue indicates men and red indicates women.

**Fig. S19** Sex-Stratified Associations Between Sodium Intake and Overall and Cause-specific Mortality in Multivariable-Adjusted Cubic Spline Regression Models, **Additionally Adjusted for Dietary Potassium Intake.**


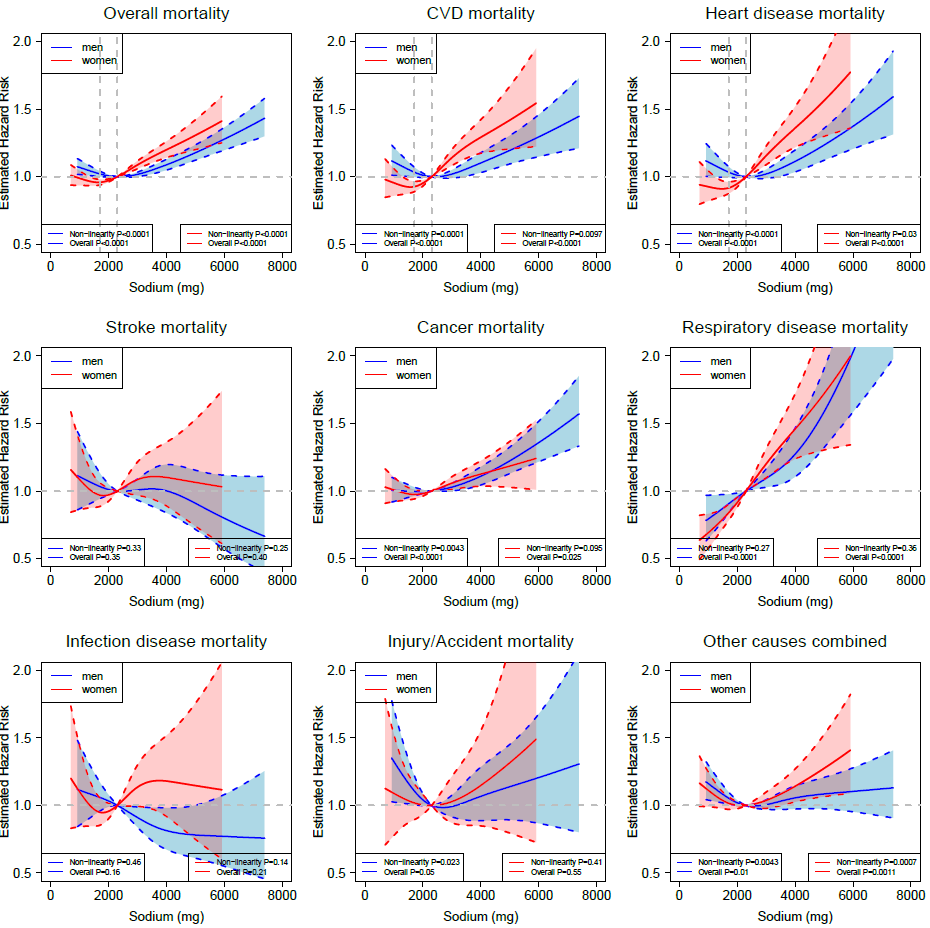


Analyses were adjusted for age at baseline, BMI, alcohol consumption, smoking status (never, former, current or missing), physical activity, race or ethnic group, education, marital status, diabetes (yes vs. no), health status, vitamin supplement use, dietary potassium intake, total energy intake, and the Healthy Eating Index 2015 (HEI-2015) score excluding the sodium component. For women, the risk estimates were additionally adjusted for postmenopausal hormone therapy (yes vs. no). The solid line denotes the HR of overall mortality according to dietary sodium intake with a four-knot cubic spline selected at the 5th, 25th, 75th, and 95th percentiles of intake, dashed lines and shaded areas represent the 95% confidence intervals, blue indicates men and red indicates women.

**Fig. S20** Flow Chat for Study Selection Strategy in the Meta-Analysis.

**
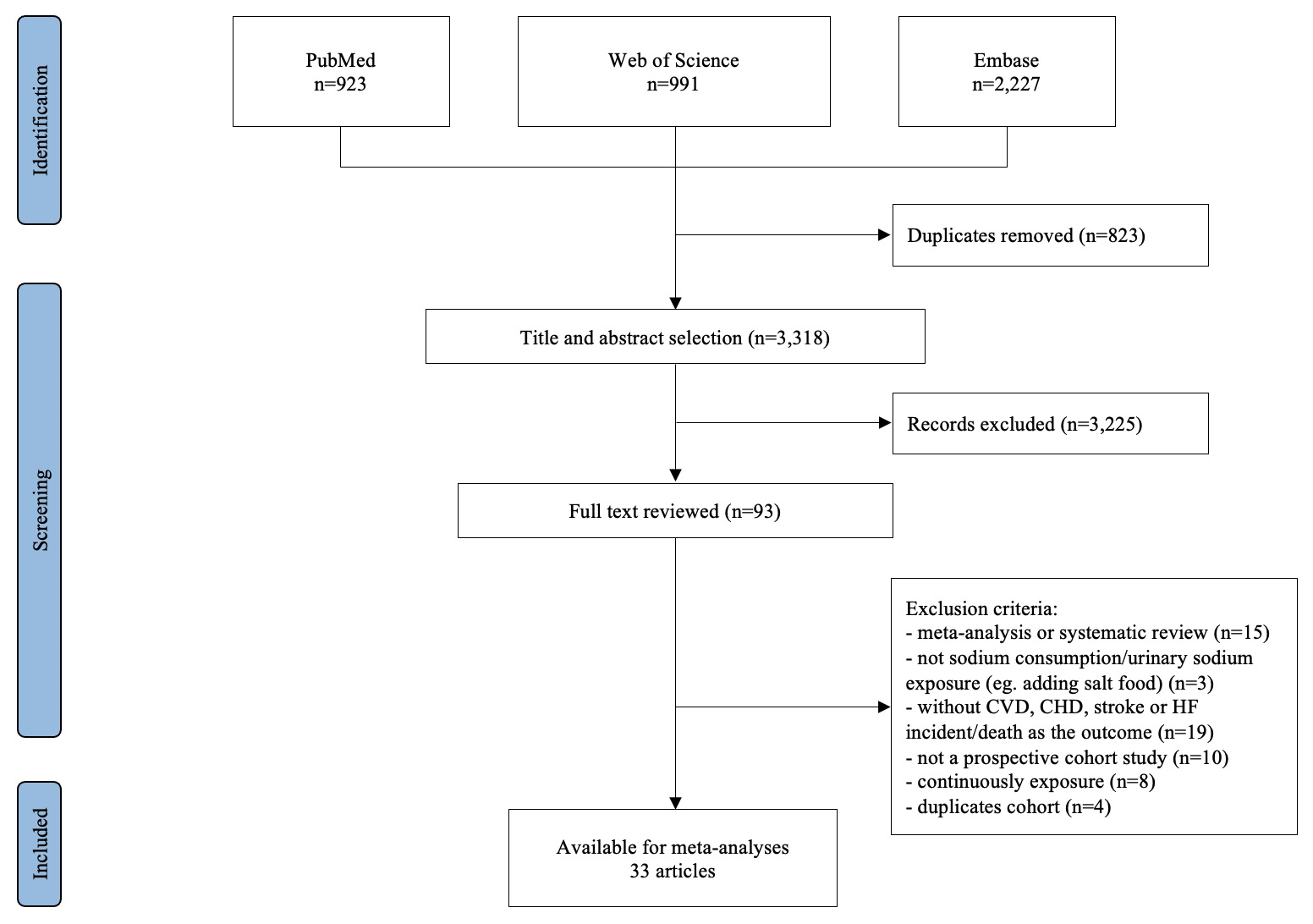
**

**Fig. S21** Funnel Plot for Assessment of Publication Bias for the Association Between Sodium Intake and Risk of Cardiovascular Disease (Including Death Due to Cardiovascular Disease).

*P* value for Egger’s test =0.10; *P* value for Begg’s test =0.069

**Fig. S22** Influence Analysis Using Forest Plot for the Meta-Analysis on Associations Between Sodium Intake and Risk of CVD (Including CVD Mortality).


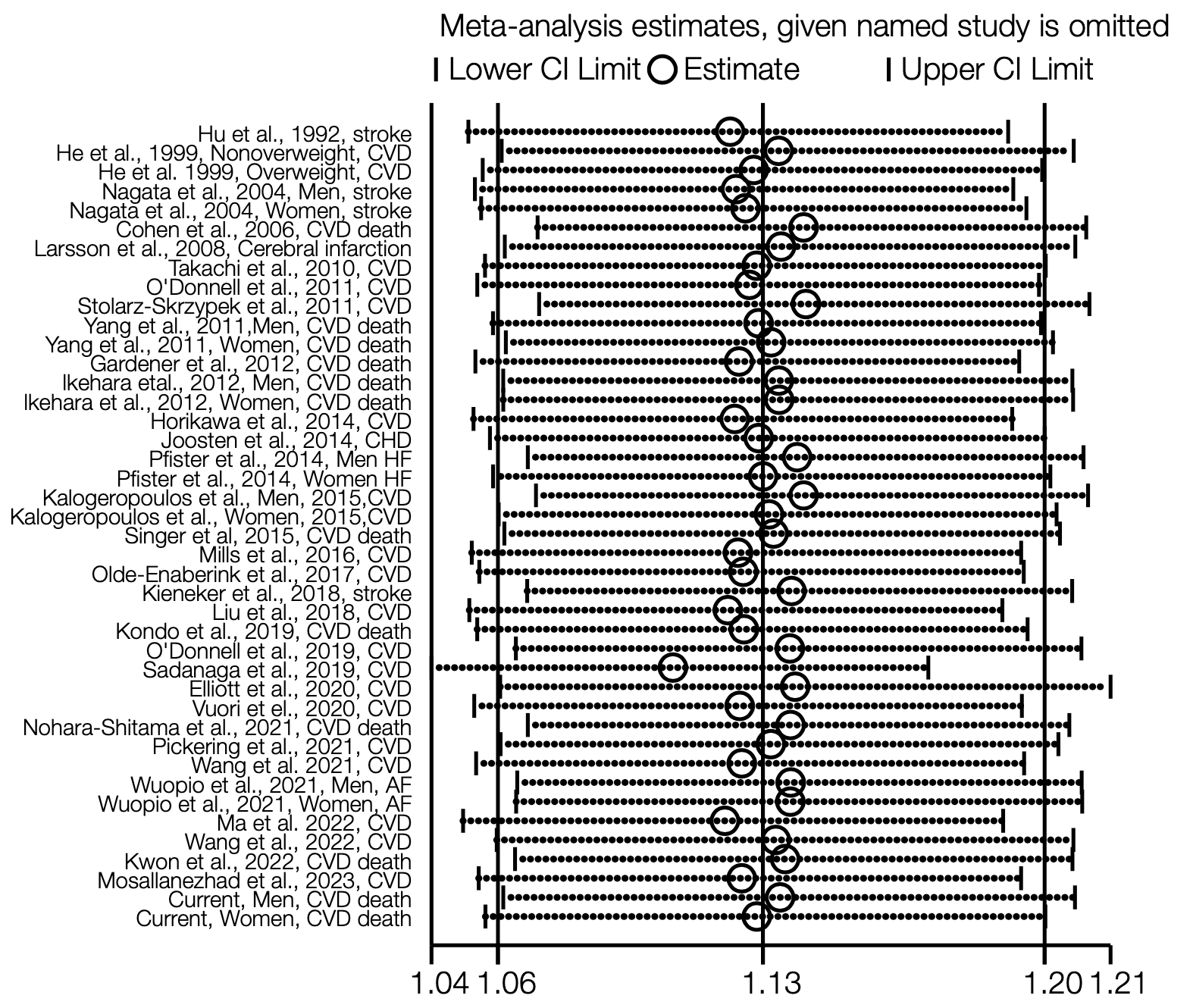


Each dot reflects the pooled RR (95% CI) following exclusion of the study listed on the left using random-effects meta-analysis.

Abbreviations: AF = atrial fibrillation; CHD = coronary heart disease; CI = confidence interval; CVD = cardiovascular disease; HF = heart failure; RR = relative risk
